# The Role of Age and Exposure to *Plasmodium falciparum* in the Rate of Acquisition of Naturally Acquired Immunity: A Randomized Controlled Trial

**DOI:** 10.1371/journal.pone.0032362

**Published:** 2012-03-07

**Authors:** Caterina Guinovart, Carlota Dobaño, Quique Bassat, Augusto Nhabomba, Llorenç Quintó, Maria Nélia Manaca, Ruth Aguilar, Mauricio H. Rodríguez, Arnoldo Barbosa, John J. Aponte, Alfredo G. Mayor, Montse Renom, Cinta Moraleda, David J. Roberts, Evelin Schwarzer, Peter N. Le Souëf, Louis Schofield, Chetan E. Chitnis, Denise L. Doolan, Pedro L. Alonso

**Affiliations:** 1 Centre de Recerca en Salut Internacional de Barcelona (CRESIB), Hospital Clínic - Universitat de Barcelona, Barcelona, Spain; 2 Centro de Investigação em Saúde de Manhiça (CISM), Maputo, Mozambique; 3 CIBER Epidemiología y Salud Pública (CIBERESP), Madrid, Spain; 4 Blood Research Laboratories, NHSBT–Oxford, John Radcliffe Hospital, Oxford, United Kingdom; 5 Nuffield Department of Clinical Laboratory Sciences, University of Oxford, Oxford, United Kingdom; 6 Department of Genetics, Biology, and Biochemistry, University of Torino Medical School, Torino, Italy; 7 Immunogenetics Research Group, University of Western Australia, Perth, Australia; 8 The Walter and Eliza Hall Institute of Medical Research, Parkville, Victoria, Australia; 9 International Centre for Genetic Engineering and Biotechnology, New Delhi, India; 10 Queensland Institute of Medical Research, Brisbane, Australia; Kenya Medical Research Institute - Wellcome Trust Research Programme, Kenya

## Abstract

**Background:**

The rate of acquisition of naturally acquired immunity (NAI) against malaria predominantly depends on transmission intensity and age, although disentangling the effects of these is difficult. We used chemoprophylaxis to selectively control exposure to *P. falciparum* during different periods in infancy and explore the effect of age in the build-up of NAI, measured as risk of clinical malaria.

**Methods and Findings:**

A three-arm double-blind randomized placebo-controlled trial was conducted in 349 infants born to Mozambican HIV-negative women. The late exposure group (LEG) received monthly Sulfadoxine-Pyrimethamine (SP) plus Artesunate (AS) from 2.5–4.5 months of age and monthly placebo from 5.5–9.5 months; the early exposure group (EEG) received placebo from 2.5–4.5 months and SP+AS from 5.5–9.5 months; and the control group (CG) received placebo from 2.5–9.5 months. Active and passive case detection (PCD) were conducted from birth to 10.5 and 24 months respectively. The primary endpoint was time to first or only episode of malaria in the second year detected by PCD. The incidence of malaria during the second year was of 0.50, 0.51 and 0.35 episodes/PYAR in the LEG, EEG and CG respectively (p = 0.379 for the adjusted comparison of the 3 groups). The hazard ratio of the adjusted comparison between the LEG and the CG was 1.38 (0.83–2.28, p = 0.642) and that between the EEG and the CG was 1.35 (0.81–2.24, p = 0.743).

**Conclusions:**

After considerably interfering with exposure during the first year of life, there was a trend towards a higher risk of malaria in the second year in children who had received chemoprophylaxis, but there was no significant rebound. No evidence was found that the age of first exposure to malaria affects the rate of acquisition of NAI. Thus, the timing of administration of antimalarial interventions like malaria vaccines during infancy does not appear to be a critical determinant.

**Trial Registration:**

ClinicalTrials.gov NCT00231452

## Introduction

In endemic areas, malaria affects primarily children younger than 5 years of age and pregnant women. Exposure to repetitive *Plasmodium falciparum* infections from birth leads to the development of naturally acquired immunity (NAI), which is rapidly acquired against the most severe forms of malaria, takes longer against milder forms and is never sterilizing [1]. Young children are at higher risk until they have developed this semi-immunity, and pregnant women temporarily lose the capacity to control the infection [1], [2]. Malaria control tools should ideally provide protection from early infancy and during the most vulnerable first years without impairing the development of NAI. The immune mechanisms involved in NAI are not well understood and immune correlates of protection against *P. falciparum* have not yet been identified in young children, the only surrogate of NAI being the risk of clinical malaria. We know the rate of acquisition of immunity predominantly depends on the intensity of malaria transmission and age of exposure, however it is difficult to disentangle the effects of these two factors and there is no clear evidence of the age interactions in the development of NAI [3].

Studies in malaria-naïve transmigrants in Indonesia [4]–[6] suggested that a more mature immune system may allow an adult to acquire immunity more rapidly than a child under the same conditions of exposure. We hypothesize that the phenomenon of age-dependent changes in the ability to acquire NAI may also apply on a reduced time scale during the first year of life.

Previous trials of malaria chemoprophylaxis and vaccines conducted in Ifakara, Tanzania, suggested that the age of first exposure to *P. falciparum* might play an important role in the development of NAI. Children who received chemoprophylaxis with Deltaprim® (Pyrimethamine plus Dapsone) from 8 to 48 weeks of age had a lower incidence of severe anaemia and clinical malaria during the first year of life compared to placebo recipients [7]. However, during the second year there was a rebound effect leading to an increased incidence of anaemia and clinical malaria suggesting that continuous chemoprophylaxis impaired the development of NAI. An extended follow-up showed that by the age of 4 years the cumulative rates of severe malaria and severe anaemia were lower among chemoprophylaxis recipients compared to controls [3]. Therefore, suppressing malaria parasites in infants during the first year by chemoprophylaxis led to an overall slightly increased cumulative rate of uncomplicated malaria by the fourth birthday but this was not associated with an increase in severity. In a separate trial, intermittent preventive treatment in infants (IPTi) with Sulfadoxine-Pyrimethamine (SP) given at ages 2, 3 and 9 months through the Expanded Program on Immunization (EPI) showed a protective efficacy for clinical malaria and severe anaemia during the first year of life compared to placebo recipients and there was no rebound effect in malaria or anaemia during the second year, indicating that development of NAI was not impaired [8]. Moreover, IPTi-SP produced a sustained reduction in the risk of clinical malaria during the second year suggesting that IPTi could actually enhance the development of NAI [9].

The efficacy provided by continuous and intermittent prophylaxis during the first year of life in these two trials was similar; what differed was the development of NAI, which was impaired in children receiving continuous chemoprophylaxis for most of the first year but was not affected in children receiving IPTi. Given that there is almost no clinical malaria in children younger than 2 months [10]–[12] and that each dose of IPTi with SP suppressed parasitaemia over an interval of approximately one month [13], the main difference between the two trials is that infants in the IPTi trial were exposed to *P. falciparum* from 4 to 9 months of age and infants in the continuous chemoprophylaxis trial were not. This difference in the age of exposure to the parasite led us to hypothesise that exposure to erythrocytic-stage antigens of *P. falciparum* during the first months of life did not contribute to the development of NAI, whereas exposure during the second half of infancy was crucial for the acquisition of NAI.

Two trials of the candidate malaria vaccine Spf66 in the same area in two different age groups further supported this hypothesis. Spf66 had an estimated vaccine efficacy (VE) of 31% (95% CI 0–52%; p = 0.046) in children aged 1 to 4 years [14], but a VE of 2% (95% CI −16–16%, p = 0.84) in infants immunized according to the EPI schedule at 1, 2 and 7 months [15]. Moreover, the breadth, intensity and longevity of antibody responses against SPf66 were significantly lower in infants as compared to older children or adults [16]. These results suggested that the immune response to the Spf66 vaccine in young infants was qualitatively different from that in older children. There is indeed evidence to suggest that immune responses in the newborn are suboptimal but that there is a potential for enhancement of these responses [17].

Taken together, data from these four trials posed a fundamental question: is there a critical time window in infancy during which exposure to *P. falciparum* enhances adequate development of NAI whereas exposure outside this window does not? If immune responses during the first months of life are impaired, would it be possible to enhance them? This has major implications for infant immunization with malaria vaccines, regarding both the timing and type of immunization, and the implementation of other malaria control tools to be administered during infancy. Accordingly we used monthly chemoprophylaxis to selectively control exposure to *P. falciparum* erythrocytic stage antigens during different periods in infancy and explored the effect of age of exposure in the build-up of NAI. The specific objective was to determine whether exposure to *P. falciparum* erythrocytic stage antigens between 0 to 5 months of age, or between 5 to 10 months of age, affected NAI, measured as the subsequent risk of clinical malaria between 12 and 24 months of age, as compared to infants with continuous exposure.

## Methods

The protocol for this trial and supporting CONSORT checklist are available as supporting information; see Checklist S1 and Protocol S1.

### Study area

The study was conducted at the Centro de Investigação em Saúde de Manhiça (CISM), located in Manhiça, Maputo Province, southern Mozambique. The area has been described in detail elsewhere [18]. Transmission of *P. falciparum* is perennial with marked seasonality and of moderate intensity, with an entomological inoculation rate of 38 infective bites/person/year [19].

CISM runs a demographic surveillance system in its study area and a morbidity surveillance system at Manhiça District Hospital (MDH) and other health posts in the area through which standardized information on all paediatric outpatient visits and admissions to hospital is collected. Recruitment and follow up of study participants were done at the Maragra Health Post (MHP), in the south of the study area, from September 2005 to March 2009.

### Ethics statement

The protocol was approved by the National Mozambican Ethics Review Committee and the Hospital Clinic of Barcelona Ethics Review Committee. The trial was conducted according to the ICH Good Clinical Practice guidelines and reviewed by a Local Safety Monitor (LSM) and a Data and Safety Monitoring Board (DSMB). The trial was registered in ClinicalTrials.gov (clinical trials identifier NCT00231452).

### Study design and procedures

#### Recruitment and randomization

**Figure 1:**
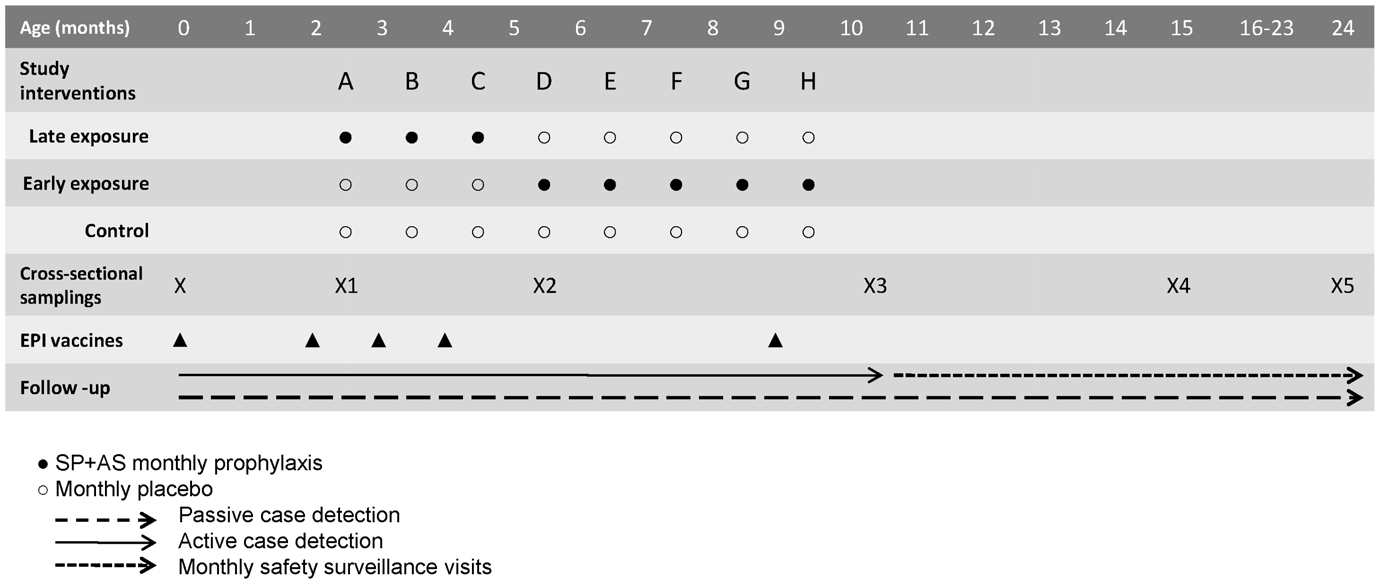
Study design.

The study was designed as a double-blind randomized placebo-controlled trial (Figure 1). HIV-negative pregnant women resident in the Manhiça study area were recruited during the third trimester of pregnancy at the antenatal clinic. Written informed consent was sought to enrol their newborn children in the study upon birth. After delivery, newborns were evaluated for eligibility; inclusion criteria included a birth weight ≥2 kg, lack of apparent health problems and having an alive mother. Exclusion criteria included birth weight <2 kg, same gender twins, congenital malformations or birth asphyxia. 349 eligible newborns were enrolled and received a sequential study number, which assigned them to one of 3 groups by block randomization (for every 6 newborns, 2 were randomized to each of the 3 groups), in order to adjust for seasonality of malaria infection. Study photo identification cards of the couple child-mother were issued to each study participant. Enrolment started in September 2005 and finished in March 2007. Investigators, clinicians involved in surveillance for endpoints, field workers, laboratory personnel and participants were blinded to the intervention group.

#### Chemoprophylaxis groups

Monthly chemoprophylaxis with SP (Fansidar® 500/25 mg) plus Artesunate (AS, Arsumax® 50 mg) or placebo (provided by Roche and Sanofi-Aventis) was administered during different periods of the first year of life according to the randomization group (Figure 1). Group 1 (Late exposure group) received monthly chemoprophylaxis with SP+AS from approximately 2.5 to 4.5 months of age (Interventions A, B and C) and monthly placebo from approximately 5.5 to 9.5 months of age (Interventions D, E, F, G and H). Group 2 (Early exposure group) received monthly placebo from approximately 2.5 to 4.5 months of age (Interventions A, B and C) and SP+AS monthly from approximately 5.5 to 9.5 months of age (Interventions D, E, F, G and H). Group 3 (Control group) received placebo monthly from approximately 2.5 to 9.5 months of age (Interventions A to H). Interventions were staggered with EPI vaccinations (given at 0, 2, 3, 4 and 9 months in Mozambique) leaving an interval of 2 weeks to avoid possible interactions with vaccine responses. Interventions were scheduled every 4 weeks, with intervention A starting at 11 weeks of age. Chemoprophylaxis was not started in children less than 11 weeks old because of the risk of kernicterus when administering SP to infants younger than 2 months and the negligible incidence of malaria in this age group in the study area [11]. Thus, given that the effect of SP+AS was assumed to last for at least one month, due to the relatively long half-life of SP [13], infants in the late exposure group were considered protected from malaria from birth until 5.5 months of age and exposed to *P. falciparum* erythrocytic stage antigens from 5.5 months onwards. In contrast, those in the early exposure group were considered exposed from 2.5 to 5.5 months and protected from 5.5 months to 10.5 months.

Drugs were administered by a field worker according to the following age-based dosing schedule: ½ tablet of SP or placebo and ½ tablet of AS or placebo on the first day and ½ tablet of AS or placebo on the second and third days. Tablets were crushed, mixed with water and administered to the child. If the child vomited, a replacement dose was administered.

During the study, Mozambique's national first line-treatment policy for malaria was SP plus Amodiaquine, which was replaced by SP plus AS in July 2006. Prior to the administration of study drugs the field worker asked the mother/guardian and checked the health card to determine whether the child had received antimalarials during the previous 2 weeks and if so, the study drugs were not administered to avoid repeating a treatment with SP.

#### Follow up

Study participants were followed up until age 24 months. Weekly active case detection (ACD) was conducted from birth to approximately age 10.5 months (4 weeks after intervention H) and monthly home visits from 10.5 to 24 months of age. ACD visits were performed at home by field workers, who measured the axillary temperature with a digital thermometer and asked the mother/guardian about history of fever. Children presenting fever (axillary temperature ≥37.5°C) or whose guardians reported history of fever in the preceding 24 h were taken to MHP, where they were examined and parasitaemia and haematocrit were determined. Antimalarials were administered according to procedures explained below if the slide reading was positive for asexual *Plasmodium* parasitaemia. During the monthly visits the mothers/guardians of the participants were asked about the occurrence of any serious adverse events (SAEs) since the previous visit and the residence status was confirmed.

Additionally, passive case detection (PCD) was carried out at the MHP and MDH through the morbidity surveillance system to monitor attendances to the outpatient clinics and admissions to hospital. Parents/guardians of study participants were asked to take their children to the MHP or MDH should they have any health problem at any time, where they were managed and treated according to Mozambican national guidelines by the attending medical personnel. Standard procedures included identification of the child, measurement of axillary temperature, completion of a standard morbidity questionnaire and physical examination. If the temperature was ≥37.5°C or there was a reported fever in the preceding 24 hours, haematocrit and malaria parasitaemia were assessed. Antimalarials were administered only if the blood slide was positive. As first line antimalarial treatment included SP, study participants who presented with malaria and had received a study intervention within the previous 2 weeks received oral quinine (10 mg/kg/8 h for a minimum of 5 days) for the treatment of their episode. Children seen at the MHP presenting severity criteria were transferred to MDH, where they were reassessed and admitted if necessary. Severe malaria in admitted patients was treated with parenteral quinine for a minimum of 6 doses, followed by SP or SP+AS when the first line treatment changed or continued up to 21 doses if given as monotherapy. Children presenting a haemoglobin (Hb) <10 g/dL on the full blood counts performed during the study cross-sectional samplings, were treated with oral iron for one month.

Safety surveillance of SAEs, including a detailed evaluation of any skin reactions potentially related to SP, was done through the PCD and during study visits. When a child presented with a skin reaction, a specific questionnaire was filled out and pictures of the lesion were taken and assessed by a study physician. SAEs were followed up by a study physician and the LSM and the DSMB reviewed all skin reaction and SAE data. Axillary temperature, weight and length were measured during five cross-sectional surveys at approximately 2.5 months of age (cross-sectional 1, at the intervention A visit), 5.5 months (cross-sectional 2, at the intervention D visit), 10.5 months (cross-sectional 3, 4 weeks after intervention H), 15 months (cross-sectional 4) and 24 months (cross-sectional 5).

#### Blood sampling

Blood samples were collected at delivery, during each cross-sectional survey, at the first clinical malaria episode and one month later. At delivery, the following samples were collected: 10 mL of peripheral venous blood (into an EDTA vacutainer), two blood slides and two drops of blood on filter paper from the mother, 8 mL of blood (into an EDTA vacutainer) and two drops of blood on filter paper from the cord, and a sample of placental tissue. When the delivery occurred outside the maternity, only the mother's sample was collected during the enrolment visit. During the cross-sectional visits, a 1 mL blood sample was collected into EDTA microtainers by finger-prick, blood spots were also collected on filter paper at cross-sectional visits 1 and 5 to determine parasitaemia by PCR and two blood smears were collected at visit 5.

### Laboratory methods

Blood slides were read to quantify parasitaemia following standard quality-controlled procedures at the CISM laboratory. Blood films were air-dried, Giemsa-stained, and examined using a light microscope fitted with a 100× oil immersion lens and a 10× eyepiece. Parasite density was assessed by counting the number of asexual stage parasites until 500 leukocytes or parasites had been counted. Slides were declared negative only after 2000 leukocytes had been counted. Parasite numbers were converted to a count/µL by assuming a standard leukocyte count of 8000/µL. All sides were read by two independent microscopists and a third reading was performed if there was discrepancy in positivity or the ratio of densities from the two readings was more than 1.5 or the absolute difference was >10 parasites/µL. The final result was based on the definitive verdict for positivity or on the geometric mean of the positive densities for positive slides. Haematocrit was measured in heparinised microcapillary tubes with a Hawksley haematocrit reader (Hawksley & Sons Ltd, Lancing, UK) after centrifugation with a microhaematocrit centrifuge. Full blood counts were performed using a Sysmex KX-21N cell counter (Sysmex Corporation, Kobe, Japan). Tissue samples were collected from the maternal side of the placenta and placed into 10% neutral buffered formalin. Biopsies were processed and analyzed following standard procedures to evaluate presence of parasites or pigment in the placenta [20]. Real-time quantitative PCR to detect *P. falciparum* was conducted with filter paper samples as previously described [21].

### Sample size

Based on results from a previous chemoprophylaxis trial [7], it was estimated that using a two-tailed test with α = 5%, 78 children per group would be needed to have 80% power to detect a relative risk of 2 in the Early exposure group as compared to the Late exposure group for at least one clinical malaria episode in the second year of life, and to detect a relative risk of 2 in the Early exposure group as compared to the Control group, assuming an incidence of clinical malaria of 0.4 episodes per person-year at risk (PYAR). To account for a 20% loss of follow up 98 children per group were needed. Sample size calculation was done using Stata software 7 (College Station, TX, USA) according to previously described methods [22]. Nevertheless, the incidence of clinical malaria in study participants during the first year of the study was lower than expected and the sample size of the study was increased to 117 participants per group to increase the power of the study.

### Data management and statistical methods

Randomization was done at CISM using Stata software 7 (College Station, TX, USA) by a statistician who was not involved in other study procedures. The code was kept by the DSMB and released to the investigators once databases had been cleaned and locked after completion of follow-up. Questionnaires were double entered into databases using a program written in Fox Pro (Microsoft Corp., Seattle, WA, USA) at CISM.

The primary case definition of a clinical malaria episode was measured axillary temperature ≥37.5°C or history of fever within the prior 24 hours plus the presence of *P. falciparum* asexual stage parasites of any density. Secondary case definitions included different parasitaemia cut-offs. Moderate and severe anaemia was defined as Hb <8 g/dL or haematocrit <25% if Hb results were not available. An intervention was defined as completed if the first day and the second and/or third day of the intervention were completed without vomiting and the interval since the previous intervention was of 3–6 weeks. The intervention was also considered complete if the study intervention was not administered because the child had received documented antimalarials during the previous 2 weeks.

The according-to-protocol (ATP) cohort included children who completed > = 75% of the interventions (≥6 of 8 interventions completed), and should there be 2 missing interventions these should not be consecutive and should not be both in the first 3 months (interventions A to C). The Total cohort included children who had received at least one intervention and the intention-to-treat (ITT) cohort included all randomized children.

Analyses were performed according to a predefined analytical plan using Stata/SE 10.1 (College Station, TX, USA). The primary endpoint of the study was the time to first or only episode of clinical malaria (according to the primary case definition) in the second year of follow up detected by PCD in the ATP cohort. The global comparison between the 3 groups and pairwise comparisons of the 3 groups are presented. Secondary endpoints included different case definitions, including multiple episodes of malaria, time to first or only episode of anaemia, total hospital visits and prevalence of parasitaemia and anaemia at different time points. Analyses were also performed in the Total and ITT cohorts and exploratory endpoints included time to first or only episode of clinical malaria during the different intervention periods of the first year of life detected by ACD or PCD in the ATP cohort.

Time at risk was calculated as the number of PYAR from the date or expected date of intervention A until the end of follow-up, migration, death or withdrawal of consent, whichever occurred first. An arbitrary lag period of 28 days was applied after a case of clinical malaria, during which children did not contribute to the time at risk. In the ATP cohort, children who were absent from the study area for >3 months did not contribute to the time at risk after the migration date.

Cox regression was used to evaluate the effect of the study intervention on the risk of first or only clinical malaria or anaemia episode. Negative binomial regression was used to evaluate the effect of the intervention on the incidence of multiple malaria episodes and total hospital visits. Logistic regression was used to compare prevalences. In the ATP cohort, the intervention effect was adjusted for use of insecticide treated nets (ITNs), indoor residual spraying (IRS), exclusive breastfeeding at 5.5 months, birth weight (Kg) and gender. Missing values in continuous covariates were imputed using the prediction from the best available subset of present data (determined by best-subset regression) in order to avoid losing observations and to compare the crude and adjusted models [23], [24].

Continuous variables were compared by ANOVA and categorical variables were compared by Chi-squared test or Fisher's exact test, as appropriate. The global comparison of the interventions was evaluated using a likelihood ratio test and a global p-value for significance. To control for family-wise error, the Bonferroni correction for multiple testing was applied to the p-values from pairwise comparisons between intervention groups. Transformation of child anthropometric data to z-scores were performed using the LMS method [25] and the reference data available from the 2000 CDC Growth Reference in the U.S. [26].

## Results

**Figure 2:**
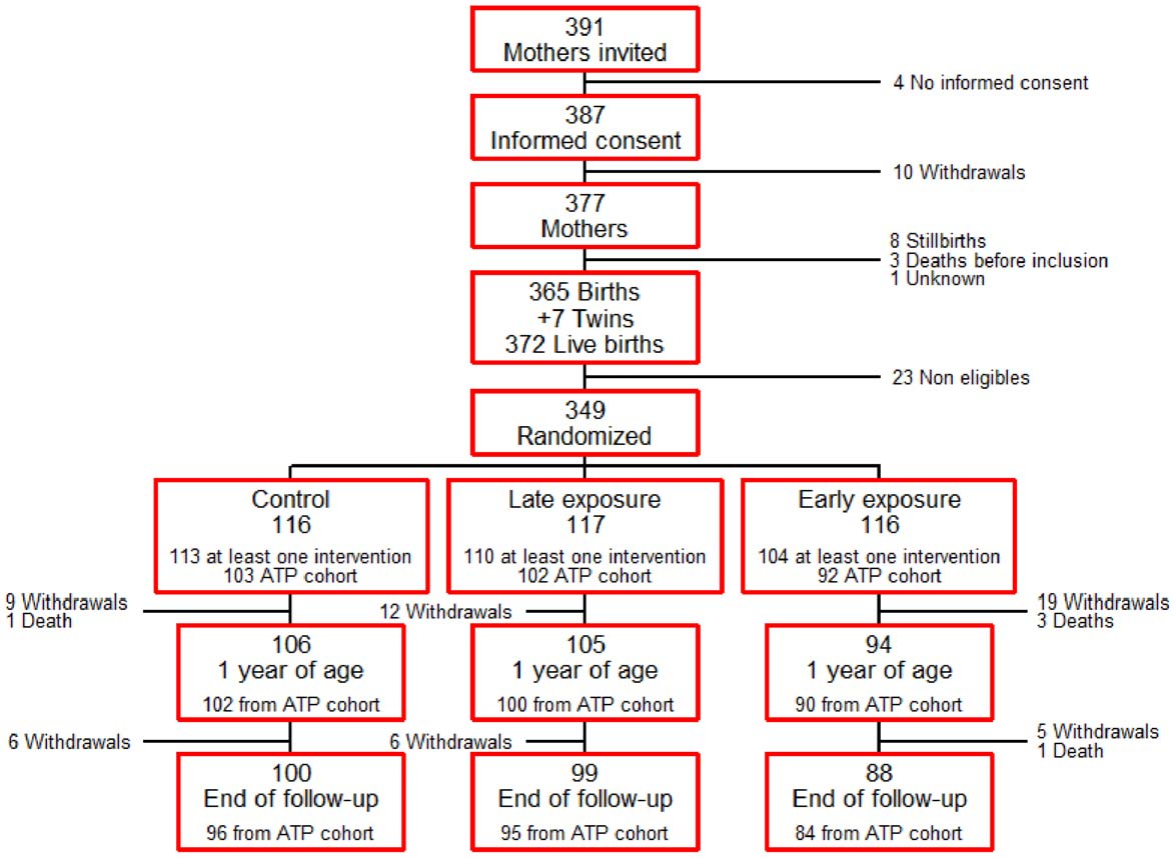
Trial profile.

Three hundred and forty-nine children were recruited and randomized into 3 groups (early exposure, late exposure and control). Figure 2 shows the trial profile. The groups were comparable for baseline characteristics of both mothers and children (Table 1 and Table 2), except for IRS of the household in the preceding year, that was higher for the late exposure group.

**Table 1:** Baseline characteristics of mothers at delivery of the three study groups in the ATP cohort.

Mother's variables		Intervention group			p-value
		Late exposure	Early exposure	Control	
Age (years)^1^		25.1 (7.1) [102]	24.3 (7.0) [92]	24.9 (6.4) [101]	0.714^2^
Parity^3^	Primigravidae	19 (18.6%)	23 (25.0%)	27 (26.2%)	0.726^6^
	Multigravidae	73 (71.6%)	62 (67.4%)	68 (66.0%)	
	Unknown	10 (9.8%)	7 (7.6%)	8 (7.8%)	
Haemoglobin (g/dL)^1^		11.4 (2.4) [80]	11.3 (2.4) [80]	11.1 (2.2) [86]	0.727^2^
Peripheral *P.falciparum* parasitemia by microscopy^3^	No	93 (91.2%)	81 (88.0%)	95 (94.0%)	0.221^4^
	Yes	7 (6.9%)	10 (10.9%)	3 (3.0%)	
	Unknown	2 (1.9%)	1 (1.1%)	3 (3.0%)	
Peripheral *P.falciparum* parasitemia by PCR^3^	No	73 (71.6%)	73 (79.3%)	77 (76.2%)	0.670^4^
	Yes	27 (26.5%)	18 (19.6%)	21 (20.8%)	
	Unknown	2 (1.9%)	1 (1.1%)	3 (3.0%)	
Parasite density (parasites/µL)^5^		7693.5 (20357.9) [7]	2473.9 (7149.5) [10]	1079.5 (2974.7) [3]	0.552^2^
Placental infection (histology)^3^	Not infected	58 (56.9%)	60 (65.2%)	72 (71.3%)	0.072^4^
	Past Infection	16 (15.7%)	15 (16.3%)	11 (10.9%)	
	Acute Infection	0 (0%)	3 (3.3%)	1 (1.0%)	
	Chronic Infection	0 (0%)	1 (1.1%)	1 (1.0%)	
	Unknown	28 (27.4%)	13 (14.1%)	16 (15.8%)	

1 : Arithmetic Mean (SD) [n].

2 : ANOVA.

3 : n (%).

4 : Fisher's exact test.

5 : Geometric Mean (SD).

6 : Chi-squared test.

**Table 2:** Baseline characteristics of children in the three study groups in the ATP cohort.

Children's variables		Intervention group			
		Late exposure (n = 102)	Early exposure (n = 92)	Control (n = 103)	p-value
Gender^1^	Male	53 (52.0%)	44 (47.8%)	51 (49.5%)	0.845^2^
	Female	49 (48.0%)	48 (52.2%)	52 (50.5%)	
Birth season	Dry	37 (36.3%)	30 (32.6%)	34 (33.0%)	0.836^2^
	Wet	65 (63.7%)	62 (67.4%)	69 (67.0%)	
Age at intervention A (months)^3^		2.6 (0.1)	2.6 (0.1)	2.6 (0.1)	0.658^4^
Birthweight (Kg)^3^		2.99 (0.42)	2.98 (0.46)	3.02 (0.38)	0.788^4^
Exclusive breastfeeding at cross-sectional 2 (5.5 months)^1^	No	53 (52.0%)	56 (60.9%)	64 (62.1%)	0.385^2^
	Yes	47 (46.1%)	32 (34.8%)	36 (35.0%)	
	unknown	2 (1.9%)	4 (4.3%)	3 (2.9%)	
Use of ITNs^1^	No	93 (91.2%)	79 (85.9%)	93 (90.3%)	0.449^2^
	Yes	9 (8.8%)	13 (14.1%)	10 (9.7%)	
Indoor residual spraying^1^	No	46 (45.1%)	49 (53.3%)	64 (62.1%)	0.050^2^
	Yes	56 (54.9%)	43 (46.7%)	39 (37.9%)	
Num. of clinical malaria episodes before intervention A^1^	No	102 (100%)	91 (98.9%)	101 (98.1%)	0.530^5^
	Yes	0 (0%)	1 (1.1%)	2 (1.9%)	
*P.falciparum* parasitaemia by PCR at cross-sectional 1 (2.5 months)	No	87 (86.1%)	77 (86.5%)	87 (85.3%)	0.969^6^
	Yes	14 (13.9%)	12 (13.5%)	15 (14.7%)	
Weight for length z-score at cross-sectional 1 (2.5 months)^3^		0.73 (1.34) [101]	0.52 (1.65) [87]	0.54 (1.31) [99]	0.527^4^
Weight for age z-score at cross- sectional 1 (2.5 months)^3^		0.30 (0.94) [101]	0.27 (1.26) [88]	0.44 (1.00) [102]	0.479^4^
Length for age z-score at cross- sectional 1 (2.5 months)^3^		−0.55 (1.15) [101]	−0.51 (1.16) [89]	−0.33 (1.17) [101]	0.332^4^

1 : n (%).

2 : Chi-squared test.

3 : Arithmetic Mean (SD) [n].

4 : ANOVA.

5 : Fisher's exact test.

6 : Log regression model using likelihood ratio test.

**Figure 3:**
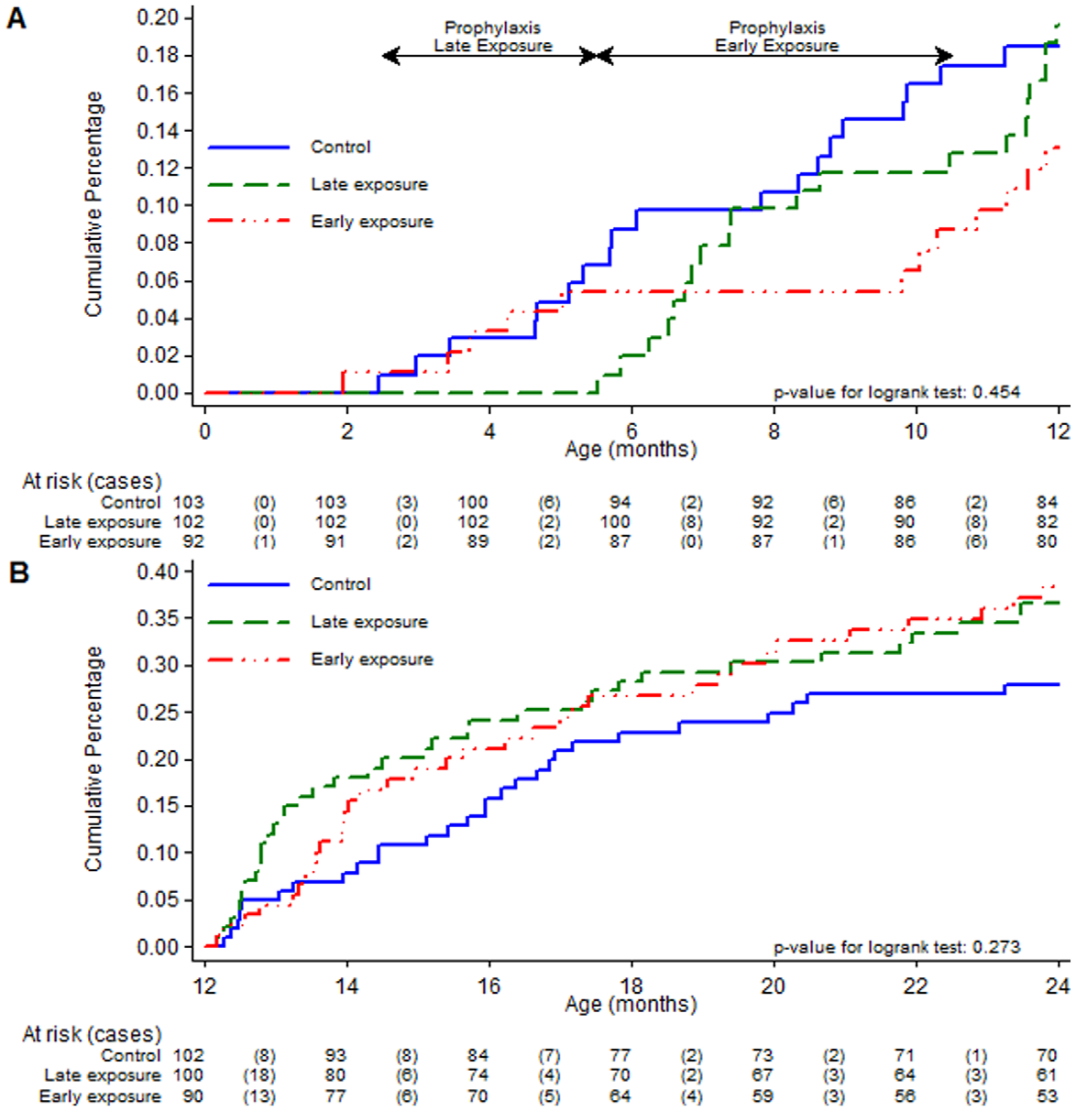
Kaplan Meier survival curves for cumulative proportion of children who had at least one episode of malaria (primary case definition) by group in the ATP cohort A) during the first year and B) during the second year of follow up.

Controlling the age of first exposure was successfully achieved with highly efficacious SP+AS chemoprophylaxis, as shown by the incidence of malaria during the intervention periods. The incidence of malaria in the late exposure group during the first intervention period (from intervention A to one month after intervention C) was 0 (0 episodes/23.97 PYAR) and in the early exposure group during the second intervention period (from intervention D to one month after intervention H) was 0.06 (2 episodes/35.72 PYAR). Figure 3A shows the Kaplan-Meier curve for the cumulative proportion of children who had at least one episode of malaria (primary case definition) by group during the first year in the ATP cohort.

There were 98 first episodes of clinical malaria meeting the primary case definition during the second year of life in the ATP cohort (36, 34 and 28 in the late exposure, early exposure and control groups respectively), yielding incidences of 0.50, 0.51 and 0.35 episodes per PYAR respectively, which were not significantly different between the 3 groups (p = 0.261 for the crude overall comparison of the 3 groups and p = 0.379 for the adjusted comparison; Table 3). The hazard ratio of the adjusted comparison between the late exposure and the control group was 1.38 (95% CI 0.83–2.28, p = 0.642) and that between the early exposure and the control group was 1.35 (95% CI 0.81–2.24, p = 0.743; Table 4), but showed no significance. Figure 3B shows the Kaplan-Meier curve for the cumulative proportion of children who had at least one episode of malaria (primary case definition) by group during the second year of follow up in the ATP cohort.

**Table 3:** Incidences of the main outcomes during the second year of follow up in the ATP cohort (adjusted analysis).

	Late exposure group (n = 100)			Early exposure group (n = 90)			Control group (n = 102)			p-value$
Outcomes	Episodes	PYAR	Incidence	Episodes	PYAR	Incidence	Episodes	PYAR	Incidence	
**First episode of clinical malaria (fever or history of fever) with >0 parasites/µL**	36	72.66	0.50	34	66.58	0.51	28	80.53	0.35	0.379*
**All episodes of clinical malaria with >0 parasites/µL**	88	90.05	0.98	91	80.12	1.14	79	92.59	0.85	0.833#
**First episode of clinical malaria with >500 parasites/µL**	27	79.18	0.34	32	68.27	0.47	23	84.38	0.27	0.231*
**First episode of clinical malaria with >2500 parasites/µL**	27	79.18	0.34	31	68.33	0.45	23	85.32	0.27	0.282*
**First episode of clinical malaria with >15000 parasites/µL**	20	83.92	0.24	27	71.22	0.38	21	86.38	0.24	0.244*
**First episode of moderate or severe anaemia**	13	89.10	0.15	10	81.50	0.12	13	92.01	0.14	0.848*
**Total outpatient visits**	365	69.73	5.23	334	62.40	5.35	338	74.34	4.55	0.335#
**Total hospital admissions**	18	95.21	0.19	17	85.42	0.20	18	97.00	0.19	0.910#

PYAR: person-years at risk.

$ Adjusted by use of ITNs, indoor residual spraying, exclusive breastfeeding at 5.5 months, birthweight (kg) and gender.

* p-value from Cox regression model using Likelihood Ratio Test.

# p-value from negative binomial regression model using Likelihood Ratio Test.

**Table 4:** Pairwise comparisons between groups of the main outcomes during the second year of follow up in the ATP cohort (adjusted analysis).

	Late exposure vs. control group			Early exposure vs. control group			Early vs. late exposure group		
Outcomes	HR or IRR$	95% CI	p-value&	HR or IRR$	95% CI	p-value&	HR or IRR$	95% CI	p-value&
**First episode of clinical malaria (fever or history of fever) with >0 parasites/µL**	1.38	0.83–2.28	0.642*	1.35	0.81–2.24	0.743*	0.98	0.61–1.59	1.000*
**All episodes of clinical malaria with >0 parasites/µL**	1.14	0.57–2.26	1.000#	1.23	0.62–2.44	1.000#	1.08	0.54–2.16	1.000#
**First episode of clinical malaria with >500 parasites/µL**	1.22	0.69–2.16	1.000*	1.60	0.93–2.75	0.273*	1.31	0.77–2.21	0.959*
**First episode of clinical malaria with >2500 parasites/µL**	1.23	0.70–2.17	1.000*	1.55	0.90–2.68	0.343*	1.26	0.74–2.15	1.000*
**First episode of clinical malaria with >15000 parasites/µL**	0.92	0.49–1.71	1.000*	1.47	0.82–2.62	0.581*	1.60	0.89–2.89	0.359*
**First episode of moderate or severe anaemia**	1.16	0.52–2.59	1.000*	0.91	0.40–2.11	1.000*	0.79	0.34–1.82	1.000*
**Total outpatient visits**	1.15	0.92–1.44	0.666#	1.16	0.93–1.46	0.549#	1.01	0.81–1.27	1.000#
**Total hospital admissions**	1.14	0.50–2.62	1.000#	1.19	0.52–2.69	1.000#	1.04	0.46–2.36	1.000#

95% CI: 95% confidence interval.

$ Hazard ratio (HR) or incidence rate ratio (IRR) adjusted by use of ITNs, indoor residual spraying, exclusive breastfeeding at 5.5 months, birthweight (kg) and gender.

& p-values corrected by Bonferroni.

* p-value from Cox regression model using Likelihood Ratio Test.

# p-value from negative binomial regression model using Likelihood Ratio Test.

Table 3 presents the incidences and global comparison between the 3 groups for secondary malaria case definitions, anaemia and total outpatient visits and hospital admissions, showing no significant difference for any of the comparisons. Table 4 presents the pairwise comparisons. For the more specific case definitions the incidence was highest in the early exposure group, but none of the comparisons were significant. The analysis of the ITT and Total cohort yielded very similar results (data not shown).

The prevalence of parasitaemia by microscopy at the last cross-sectional sampling (24 to 26 months of age) also showed no differences between the three groups (13.04%, 13.95% and 7.45% in the late exposure, early exposure and control groups respectively, p = 0.302; Table 5). Table 5 also presents the prevalence of anaemia, mean Hb, geometric mean parasitaemia and anthropometric measures at the last cross-sectional visit in the ATP cohort, for which there were no differences between the groups. Pairwise comparisons also showed no significant differences for these outcomes (data not shown).

**Table 5:** Prevalence of anaemia and parasitaemia, mean heamoglobin, geometric mean parasitaemia and anthropometric measures at the last cross-sectional visit (24 to 26 months) in the ATP cohort.

		Late exposure	Early exposure	Control	p-value
Haemoglobin (g/dL)^1^		11.4 (2.4) [80]	11.3 (2.4) [80]	11.0 (2.2) [88]	0.569
Moderate or severe anaemia^3^	No	86 (93.5%)	80 (93.0%)	83 (88.3%)	0.388^4^
	Yes	6 (6.5%)	6 (7.0%)	11 (11.7%)	
*P.falciparum* parasitaemia by microscopy^3^	No	80 (87.0%)	74 (86.1%)	87 (92.5%)	0.302^4^
	Yes	12 (13.0%)	12 (13.9%)	7 (7.5%)	
*P.falciparum* parasitaemia by PCR^3^	No	64 (69.6%)	55 (63.9%)	55 (58.5%)	0.290^4^
	Yes	28 (30.4%)	31 (36.1%)	39 (41.5%)	
Parasite density (parasites/µL)^5^		2139.4 (4425.1) [12]	1959.9 (4788.6) [12]	6594.8 (19096.2) [7]	0.535^2^
Weight for length z-score at cross-sectional 1^1^		−0.33 (1.18) [91]	−0.36 (1.23) [86]	−0.42 (1.23) [91]	0.888^2^
Weight for age z-score at cross-sectional 1^1^		−1.36 (1.14) [93]	−1.32 (1.17) [84]	−1.24 (1.33) [93]	0.804^2^
Length for age z-score at cross-sectional 1^1^		−1.40 (1.09) [91]	−1.46 (1.06) [86]	−1.19 (1.17) [91]	0.226^2^

1 : Arithmetic Mean (SD) [n].

2 : ANOVA.

3 : n (%).

4 : Log regression model using likelihood ratio test.

5 : Geometric Mean (SD).

There were no safety concerns during the trial. There were 62 skin reactions during the intervention period (up to one month from last dose) in the total cohort, but only one (urticaria with pruritus) was assessed to be possibly related to the intervention and all resolved without sequelae. The occurrence of SAEs was similarly distributed among the groups, and none was deemed related to study drugs. There were 5 deaths during the complete follow up, none considered related to the intervention and 3 occurring in children who had not yet received an intervention.

## Discussion

This study used monthly antimalarial chemoprophylaxis to control exposure to the erythrocytic-stage antigens of the *Plasmodium* parasite. By selectively administering chemoprophylaxis to children during different periods of their first year of life, we evaluated the importance of age of first exposure to malaria in the development of NAI during infancy. NAI was measured as incidence of clinical malaria during the second year of life, as there are no surrogate markers of protection for *P. falciparum*, and comparing clinical malaria between groups is the only way to evaluate acquisition of immunity. Our hypothesis was that exposure to malaria during the first months of life would not lead to acquisition of NAI, while exposure during the second half of infancy would lead to and play a crucial role in the development of NAI.

Compliance with study drugs among participants was high, and chemoprophylaxis was well tolerated and highly efficacious, as proven by the near zero incidence of malaria during the different time periods in which children received SP+AS. Therefore, from two to twelve months of age the time of exposure to the parasite was decreased by 50% in the early exposure group and by 30% in the late exposure group compared to the control group. However, we did not find any significant differences in the outcomes between the three different study groups. Indeed, there were no significant differences between the groups in the incidence of clinical malaria, anaemia, total outpatient visits or hospital admissions during the second year of life. Also, there were no differences in the prevalence of parasitaemia or anaemia at the end of follow up.

Although the incidence of malaria during the second year was higher by about 40% in the late exposure and early exposure groups when compared to the control group, and the risk of malaria was highest in the early exposure group for the more specific case definitions, none of these differences reached statistical significance. The sample size had been calculated to detect a relative risk of 2 between the groups, based on results from a previous continuous chemoprophylaxis trial [7]; however, the observed differences were lower and the study was underpowered to evaluate the significance of the observed estimate of 1.4. Hence, the differences seen between the groups might reflect a real difference that would only be significant with a much larger sample size or a higher transmission intensity.

Our hypothesis was based on results from previous malaria chemoprophylaxis and vaccine trials conducted in Tanzania in infants and young children. Data showed that infants receiving IPTi [8] but not those on continuous chemoprophylaxis up to 11 months of age [7] were able to develop protective immunity. However, the long-term follow-up of participants in the latter trial showed that, although there was a rebound effect in the second year of life, the cumulative rate of severe malaria or anaemia after four years was actually lower in children that received chemoprophylaxis during most of the first year [3]. Indeed, in the last few years there has been a debate around interference of the development of NAI due to interventions and rebound effect [3]. In our study, despite significantly decreasing exposure to the parasite during the first year of life, we did not see a significant impact on the incidence of malaria during the second year. Nevertheless, there was a trend towards rebound in the second year, but even if the hazard ratios of 1.4 observed between the late or early exposure and the control group were significant, the rebound effect would be much lower than that anticipated based on previous results [7]. Therefore, a massive reduction in exposure, such as that obtained with continuous chemoprophylaxis during most of the first year, seems to be necessary to see a rebound, suggesting that a few infections are enough to build NAI, although this rebound in the second year did not have an impact in an increased rate of severe disease in the long term [3].

Taking together the results of the three trials, data suggest that contact with the parasite is needed at some point during infancy to be able to develop NAI, but that the timing of the exposure does not seem to be a major contributing factor. Clearly, no differences in the incidence of malaria during the second year were seen between the early and late exposure groups, and young infants with relatively immature immune systems and low exposure were able to build NAI as well as older infants. With available data it is not possible to establish the amount of exposure that is needed for the development of NAI, although exposure in our trial and in the Tanzanian IPTi trial varied from 5 to 7 months (from 2 to 12 months of age). Infants in these two trials received comparable challenge during the first year (the incidence of multiple malaria episodes during the first year was 0.33 episodes per PYAR in the control group in the former (data not shown) and 0.43 in the latter [8]), but it is difficult to compare the amount of exposure in both studies. SP, that was used for IPTi, had a much lower efficacy than SP+AS, which was highly efficacious at the time of the study in Mozambique [27]. However, resistance to SP alone was also high in Mozambique at the time of the study, so it is possible that it was at some point breached by resistant parasites. Nevertheless, should this have happened, the duration of the parasitaemia would have been short, as the monthly administration of AS would have probably cleared the parasites. Thus children in our trial probably had a very low exposure to blood-stage malaria antigens while receiving chemoprophylaxis, whereas it has been argued that SP in the IPTi trial could have acted as a leaky vaccine, allowing exposure to low dose parasitaemias in study participants [9].

Overall, no evidence was found that the age of first exposure to the parasite during the first year of life affects the rate of acquisition of NAI against malaria. Thus the timing of administration of antimalarial interventions like malaria vaccines during infancy does not appear to be a critical determinant, as infants should be equally able to mount an adequate immune response both during the initial months of life and later. Indeed, recent results from phase I/IIb trials of the RTS,S/AS0 candidate malaria vaccine conducted in endemic areas of Africa support this. In the RTS,S/AS0 trials performed after our trial had started, VE was similar in three trials in which the vaccine was administered to infants at different ages: 10, 14 and 18 weeks of age, staggered with EPI vaccines (VE against infection 65.9, 95% CI 42.6–79.8; p<0.0001) [28]; at 8, 12 and 16 weeks of age together with EPI vaccines (VE against infection 65.2, 95% CI 20.7–84.7; p = 0.01) [29]; and at 5 to 17 months (VE against clinical malaria 53%, 95% CI 28–69, p<0.001) [30]. Vaccine efficacy in these infant trials was similar or higher to that in children aged 1 to 4 years (VE against infection 45.0, 95% CI 31.4–55.9; p<0.0001 and VE against clinical malaria 29.9%, 95% CI 11.0–44.8; p = 0.004) [19], [31]. Although the potent GSK Biological's AS0 adjuvants with which RTS,S was formulated in these trials could have played a role in overcoming possible suboptimal immune responses in young infants, it appears the vaccine can also be significantly protective in newborns with immature immune systems. This confirms that the administration of malaria vaccines through the EPI would be the best choice to maximize compliance and decrease logistic costs.

Secondary objectives of the study included assessment of type and quality of immune responses, oxidative stress markers and host genetic factors, aiming at describing these outcomes according to age of first exposure and correlating them with clinical malaria during the second year of life. Results of these analyses will be presented in other specific papers and will hopefully shed light on the age-dependency of the immune responses and how those correlate with protection or susceptibility against malaria. Any step towards better understanding the development of NAI in infancy will enhance our chances of improving the current preventive tools and strategies against this deadly parasite.

Therefore, from this study we can reasonably conclude that no evidence was found that the age at which the first encounter with the parasite occurs during infancy is a key determinant in the build up of immunity. Secondly, considerable reductions in exposure to parasite antigens in the first year of life do not result in large rebound in the incidence of malaria during the second year and massive reductions in exposure are needed to see a significant impact on the acquisition of NAI.

## Supporting Information

Checklist S1 **CONSORT Checklist.**
(DOC)

Protocol S1 **Trial protocol.**
(PDF)
