# Supplementary material for: The Role of Age and Exposure to Plasmodium falciparum in the Rate of Acquisition of Naturally Acquired Immunity: A Randomized Controlled Trial
Source: PLoS One. 2012 Mar 7;7(3):e32362. doi: 10.1371/journal.pone.0032362 (PMC3296698; doi:10.1371/journal.pone.0032362)
Supplement: Protocol S1 — Trial protocol. (PDF) [file pone.0032362.s002.pdf]

Age of exposure and immunity to malaria in infants

Date of final protocol: 2nd July 2004
Date of amendment 1: 15 October 2004
Date of amendment 2: 2 December 2004
Date of amendment 3: 11 July 2005
Date of amendment 4: 10 August 2005
Date of amendment 5: 12 September 2006

Principal Investigator: Carlota Dobaño[1,2]***, Pedro L. Alonso***[1,2,3]
Research team: ~~Pedro L. Alonso~~, Caterina Guinovart[1,2], ~~Artur Machava~~, John Aponte[1,2], Clara Menéndez[1,2], Diana Quelhas[1,2], Jahit Sacarlal[2,4], ***~~Fatima Abacassamo,~~ Pedro Aide***[2,3]***, Climent Casals***[1,2]***, Alfredo Mayor***[1,2]***, David Roberts***[5]***, Evelin Schwarzer***[6]***, Peter Le Souef***[7]***, Louis Schofield***[8]***, Chetan Chitnis***[9]***, Denise L Doolan***[10] **(Amended 15 October, 2004) (Amended 10 August, 2005)**

[1]Centre de Salut Internacional (CSI), Hospital Clínic, Barcelona, Spain
[2]Centro de Investigação em Saúde de Manhiça, Mozambique
[3]Ministerio de Saúde, Mozambique
[4]Faculdade de Medicina, Universidade Eduardo Mondlane, Mozambique
[5]Blood Research Laboratories, University of Oxford, UK
[6]Dept. Genetics, Biology and Biochemistry, Università di Torino, Italy
[7]Immunogenetics Research Group, Univ. Western Australia, Perth, Australia
[8]The Walter and Eliza Hall Institute of Medical Research, Parkville, Victoria, Australia
[9]International Center for Genetic Engineering and Biotechnology, Delhi, India
[10]Naval Medical Research Center, Silver Spring, USA
(Amended 10 August, 2005)

Title: Age of exposure and immunity to malaria in infants

RESEARCH PLAN (Sections A-D of the proposal)

SUMMARY

The role of age in the development of naturally acquired immunity ***(NAI)*** to malaria is not clear. In previous studies conducted by investigators from this group, continuous or intermittent malaria prophylaxis was used to independently affect the timing of first exposure and the duration of exposure to *Plasmodium falciparum* (Pf) parasites in cohorts of infants and young children. Specifically, those studies suggest that (i) exposure after approximately 5 months of age is critical to the development of protective immunity to malaria [1], while (ii) exposure to Pf prior to approximately 5 months of age does not contribute to the development of NAI [2], as measured by risk of clinical episodes of malaria during the second year of life. On the basis of those findings, we hypothesize that inherent age-related differences in the immune responses to initial exposure to malaria antigens influence the subsequent risk of clinical disease.The overall objective of the proposed study is to investigate the effect of exposure to Pf erythrocytic stage antigens during defined periods of infancy upon the development of naturally acquired immunity (NAI) to malaria. The study is designed as a double-blind randomized placebo-controlled trial in infants born in a malaria endemic rural region of southern Mozambique. Infants will be recruited at birth from HIV-negative mothers and will be randomly allocated to one of three cohorts, with ~~77~~ ***98*** infants each, and will be followed by active and passive case detection up to ***10 months of age and by passive case detection and monthly safety surveillance home visits from 10 months up to*** 25 months of age. Cohort 1 will receive monthly chemoprophylaxis with Sulphadoxine-Pyrimethamine (SP) + Artesunate (AS) from ~~2 to 5~~ ***2,5 to 5,5*** months of age and placebo from ~~5 to 10~~ ***5,5 to 10,5*** months of age, cohort 2 will receive placebo from ~~2 to 5~~ ***2,5 to 5,5*** months of age and SP+AS from ~~5 to 10~~ ***5,5 to 10,5*** months of age and cohort 3 will receive placebo from ~~2 to 10~~ ***2,5 to 10,5*** months of age. By selectively controlling exposure, using differential administration of chemoprophylaxis to infants, we will evaluate the effect of age on the development of protective immunity to malaria. The risk of clinical malaria and anaemia during the second year of life and the type and quality of immune responses will be compared between cohorts. The central hypothesis of this proposal is that exposure to Pf erythrocytic stage antigens in early infancy does not contribute to the development of naturally acquired immunity to malaria but exposure after 5 months of age does contribute to the development of naturally acquired immunity. **(Amended 15 October, 2004) (Amended 2 December, 2004) (Amended 10 August, 2005)**

A) BACKGROUND AND SIGNIFICANCE

RATIONALE: There has been long-term speculation as to the role of age in determining the rate of acquisition of protective immunity to malaria. The evidence to date is limited and difficult to interpret. However, gaining an insight into this issue is paramount to future intervention strategies for malaria and specifically, to vaccine applications. In general, the best way to establish a causal relation between two events is by carrying out an intervention study. For this reason, we plan to specifically manipulate the age at which infants born in an endemic area encounter malaria parasites. By selectively controlling exposure to malaria in young infants who subsequently have continual and long-term exposure to Pf parasites, we can investigate the effect of age on the acquisition of protective immune responses.

SUPPORTING DATA IN INFANTS: The hypothesis regarding age-related differences during infancy that affect the ability to develop NAI is supported by two sets of data derived from investigators

engaged in this proposal, the first derived from trials of the malaria vaccine SPf66, and the second from trials of malaria prophylaxis. In placebo-controlled, double-blind studies in Tanzania of SPf66, a peptide vaccine consisting primarily of Pf blood stage antigens, estimated vaccine efficacy was 31% (95% confidence interval [CI] 0--52%; p = 0.046) in children aged 1-4 years [3] but was 2% (95% CI –16--16%; p = 0.84) in 6-12 month olds infants immunized according to the EPI schedule [4]. These results suggest that the immune system of young infants is qualitatively different from that of older children.

Data from studies of continuous and intermittent anti-malarial treatment conducted in a malaria-hyperendemic area of Tanzania also support the concept that there is a critical time period during infancy when acquisition of immunity occurs [1, 2]. The first study [1], conducted in 832 infants, showed that malaria prophylaxis with Deltaprim® provided weekly from 8 to 48 weeks (2-11 months) was effective in reducing the frequency of episodes of severe anemia (0.45 vs. 1.04 episodes per person-year; protective efficacy 60.5% [48.2-69.9]) and of clinical malaria (0.53 vs. 1.34 episodes per person-year; protective efficacy 57.3% [43.0-67.9]) in the first year of life compared with placebo. However, during the second year of life, those children who had received prophylaxis as infants had higher rates of severe anemia and malaria than children who did not receive prophylaxis (relative risks 2.2 [1.3-3.7] and 1.8 [1.3-2.6] respectively).

These children were then followed up until their fourth birthday. The results (Alonso et al, submitted for publication) of this extended follow-up indicate that chemosuppressing infants during most of the first year of life leads to an overall slightly increased cumulative rate of uncomplicated malaria by the fourth birthday but that this is not associated with an increase in severe malaria or severe anemia. Indeed, when children were aged 4 years the cumulative rate of severe malaria episodes (rate difference -0.12, 95% CI -0.27-0.03) and that of severe anaemia (rate difference -0.19 (95% CI -0.40-0.01) was lower in the intervention cohort, reassuring us on the benefits of exposure reduction interventions.

These data suggest that malaria chemoprophylaxis administered consistently between 8 and 48 weeks (2 to 11 months), while providing protection against disease in the first year of life, impaired the development of NAI.

A follow-up study, performed at the same site in Ifakara, Tanzania, addressed the question of whether intermittent prophylaxis (IPTi), as opposed to continuous prophylaxis, might maintain the benefits during the first year of life without engendering a rebound effect during the second year [2]. This study, conducted in 701 infants, showed that intermittent prophylaxis with SP at 2, 3 and 9 months of age (with suppression of parasitemia presumably lasting for approximately one month after administration of each SP dose [5]) was also effective in reducing the frequency of episodes of severe anemia (0.06 vs. 0.11 episodes per person-year at risk; protective efficacy 50% [95% CI 8-73]) and of clinical malaria (0.15 vs. 0.36 episodes per person-years at risk; protective efficacy 59% [95% CI 41-71]) in the first year of life, compared with administration of placebo. However, in contrast to the first study, there was no evidence of a rebound effect with regard to clinical malaria or anemia during the second year of life in the group who received the intermittent prophylaxis as compared to those who received placebo.

During the extended follow-up of the participants in this trial (Schellenberg et al, submitted for publication), who were followed up to age 24 months, IPTi produced a sustained reduction in the risk of clinical malaria during the second year of life, with a protective efficacy of 35.7% (95% CI 12, 52). These results show there is a sustained reduction in the risk of clinical malaria in children receiving IPTi extending well beyond the duration of the pharmacological effects of SP.

Surveillance methods in both studies were similar, both drug regimens appeared highly effective in compliant study subjects, and levels of drug-resistance are low in Ifakara. Therefore, data suggest that infants receiving intermittent prophylaxis, but not those on continuous prophylaxis up to 48 weeks

of age were able to develop protective immunity, although the cumulative rate at age 4 of severe malaria or severe anaemia was not increased. It should be noted, however, that the overall malaria attack rate was lower in the second study as compared with the first, and this may have influenced the study outcomes. Nonetheless, data suggest that **exposure to Pf erythrocytic stage antigens between approximately 4 to 11 months of age may be critical for the development of NAI, while exposure prior to this age is not**.

Taken together, data from the vaccine and prophylaxis studies in infants pose a critically important question: when in infancy does exposure to Pf parasites enhance the development of NAI, and therefore should be preserved (at least to some degree) as part of an overall public health strategy to diminish morbidity and mortality, and when in infancy is exposure irrelevant and thus safely avoided through the use of chemoprophylaxis? The study proposed here is designed to address this question.

WHAT IS NAI? People living in malaria endemic areas of sub-Saharan Africa who are exposed to repeated Pf infections from infancy develop clinical immunity to the disease [6]. In areas with year-round, stable transmission, neither severe malaria nor malaria-associated deaths occur after the age of about 5 years, and incidence, prevalence and density of infection decrease with age [7]. In areas with the most intense transmission, the transition to this immunity against malaria occurs even earlier, perhaps during the second year of life. This form of immunity, referred to as "anti-disease immunity", develops as a result of heavy, uninterrupted exposure to infection and can lead to sustained periods without clinical symptoms in older children and adults, even though clinical protection is probably never complete. Immunity to disease is generally accompanied by a substantial decrease in the parasite density referred to as "anti-parasite immunity" that also develops with increasing age. The acquisition of protective anti-malarial immunity appears to be dependent on both age and duration of exposure, and to date it has been difficult to independently measure the contribution made by these two variables.

INFLUENCE OF HOST AGE ON NAI: Epidemiologic studies of malaria-naive Javanese transmigrants in Irian Jaya conducted by US Naval Medical Research Unit between 1987 and 1999 suggest that immunity to malaria develops differently in adults and children [8, 9] and that a more mature immune system may allow an adult to acquire immunity more rapidly than a child under the same conditions of exposure. Acquired immunity in the transmigrants was not the cumulative product of many years of heavy exposure, but was influenced by intrinsic features of the acquired immune response that change with age. Another finding was that age-dependent immunity occurs with both acute and chronic exposure to infection, with differences between children and adults. Under conditions of chronic exposure, adults more rapidly develop anti-parasite immunity compared with children, i.e., after about 18 months, they have fewer and lower density parasitemias with reduced clinical symptoms. Acute exposure showed an inverse pattern of relative susceptibility, i.e., higher risk of severe disease among adults with first infections [10]. This was evidenced during an epidemic of falciparum malaria [11] by the higher risk of emergency medical evacuations due to severe malaria among adult transmigrants compared to their children. Thus, adults apparently are better able to adapt to repeated exposure, whereas children do better with initial exposure [12]. Other investigators have proposed that intrinsic features of the immune system that change with age could be explained, for example, by non-protective antibody isotypes produced during childhood that might block the activity of "protective" isotypes [13]. The phenomenon of age-dependent changes in the ability to acquire NAI may also apply on a finer time scale during the first year of life; investigating this hypothesis is the aim of the proposal.

IMMUNOLOGIC CORRELATES OF NAI: Little is currently known about the underlying mechanisms of NAI. The only studies that have clearly documented in humans the importance of a specific arm of the immune system in protection against malaria have been the classical studies of passive transfer of purified immunoglobulin (Ig) in which transfer of pooled γ-globulin from West African immune adults into East African or Thai children acutely infected with *P. falciparum* caused a sharp drop in parasitemia [14, 15, 16, 17]. Despite years of studies of human immune response to defined *Plasmodium* sp. antigens, aiming to correlate a response with apparent clinical status and thus to identify a protective role for that response, it has not been possible to define with any certainty which antigens contribute to NAI, nor to identify any immune response which correlates with NAI. Given the complexity of the parasite and the multitude of immune responses which may be involved, it is unlikely that a single immune response directed against a single antigen expressed during one stage of the parasite's life cycle is responsible for protection. Most studies have only looked at responses against single antigens, including MSP-1 [18, 19, 20], LSA-1 [21], CSP [22], and MSP-3 [16]. Moreover, studies have been limited in that they have not identified and controlled for the many potential confounding factors, including exposure, and in many cases, age. Several studies conducted in the 1970's and 1980's in West Africa focused on longitudinal follow-up of serological responses in infants [23, 24, 25] and older children [26] receiving anti-malarial prophylaxis, but these studies did not control for age or exposure.

Anti-parasite antibody responses - Anti-PfEMP1 antibodies: In order to assess the development of NAI in infants, we propose to measure those parameters that have been shown to be appropriate surrogates of protective immunity against malaria, in particular antibodies agglutinating infected erythrocytes. In the Gambia, of various measurements of immunity to blood-stage antigens (including ELISA and IFAT for antibodies to whole schizonts or individual antigens, growth inhibition assay, plasma agglutination assay, and opsonization assay), the only predictive index for protection against clinical malaria as measured by reduced episodes of clinical disease was found to be the titer of agglutinating activity in the plasma at the onset of the transmission season [27]. Marsh & Howard suggested that susceptibility of the acutely infected children might be due, at least in part, to the fact that they had not previously experienced infection with the same "strain" [28]. Support for this hypothesis came from more recent studies in Kenya, where it was shown that variant surface antigens expressed during episodes of clinical malaria in children were less likely to be recognized by the preexisting antibodies in the same child than that by other children [29]. Another study in Sudan that measured the levels of IgG against variant antigens on the surface of Pf-infected erythrocytes by flow-cytometry found an association between presence of such antibodies and protection from novel parasite clones expressing homologous, but not heterologous antigens [30].
Consensus of opinion now favors the hypothesis that the variant surface antigen PfEMP-1 is an important target for naturally acquired immunity, and data suggest that the antibody-mediated agglutination assay [29] and the flow cytometry [30, 31] are appropriate methods to evaluate the development of anti-PfEMP1 antibodies that are thought to provide variant-specific protection against disease [28]. In The Gambia [28] and in Papua New Guinea (PNG) [32, 33], plasma from children in the convalescent stage of infection reacted with erythrocytes infected with autologous but not with heterologous malaria strains in the antibody-mediated agglutination assay. In contrast to plasma from children, adult immune plasma contained antibodies that recognized the majority or all of the isolates and reacted with the surface of infected cells from most children in The Gambia [28], PNG [32], and Venezuela [34]. These data are consistent with either the acquisition of immunity against a large number of antigenically diverse variant proteins with time and/or exposure, or else with the gradual development of strain or variant transcendent responses presumably recognizing conserved regions of parasite antigens. There is evidence suggesting that the human agglutinating antibody response is predominantly variant specific, since antibodies which cross-react between different serotypes are

rare and react only with a subset of infected erythrocyte surface antigens [35]. Two field studies support the idea that exposure to a wide range of variant types is essential for the developing of NAI. In Kenya, there was an apparent expansion of antibody repertoire against variant surface antigens between 1-5 years, which is the age range when disease immunity develops in that region [29]. In Ghana, the levels of IgG to variant antigens expressed on the surface of infected erythrocytes (as assessed by flow cytometry) correlated with protection from clinical malaria, whereas levels of IgG to conserved PfEMP-1 epitopes (as assessed by ELISA) did not correlate with protection [31]. Taken together, available data support the hypothesis that Pf antigens on the surface of PRBC are targets of NAI to malaria and that flow cytometry is suitable for measuring protective immune responses directed against these antigens.

(Amended 11 July, 2005)

Anti-disease Antibody responses - Anti-hemozoin and anti-GPI antibodies: The temporal correlation between schizont rupture and acute febrile episodes, with sharp increase in the concentration of circulating cytokines, is consistent with the view that parasite products released from infected erythrocytes at the time of schizogony may trigger the inflammatory cytokine cascade, leading to the onset of symptoms. Soluble parasite products or "toxins" can activate macrophages to release TNF-α, IL-1 and IL-6, and may directly activate the vascular endothelium and upregulate expression of adhesins such as intercellular adhesion molecule 1 (ICAM-1) and VCAM-1 [91], which can mediate parasite cytoadherence, thereby contributing to organ-specific and cerebral disease syndromes [97]. Furthermore, both cytokines and malarial toxins can each directly induce the expression of other pro-inflammatory loci such as that for inducible nitric oxide synthase (iNOS), thereby raising levels of nitric oxide(NO), which may be a regulator of pathogenesis. There is growing evidence sustaining the idea that antibodies to TNF-α-stimulating malaria toxins may explain the phenomenon of clinical tolerance in malaria, and it may be possible to develop an anti-disease vaccine based on modified forms of these toxins. We propose to measure acquired immune responses to hemozoin andGPI, two candidate malaria toxinsthat are thought to contribute to the development of disease and to be targets of clinical immunity [90-97]. The malarial pigment (haemozoin) may be a factor in the production of the malarial paroxysm, and may have a role in the pathogenesis of malaria anemia by decreasing red blood cell deformability through oxidative damage of infected erythrocytes, and by inhibiting erythropoiesis. Immunization against chemically synthetic parasite GPI confers protection against cerebral pathology, acidosis, and pulmonary edema in the best available small animal model of severe and fatal pathogenesis [97]. Lacking memory responses, anti-GPI antibodies may decay rapidly and thus be particularly exposure-dependent, and thus be especially sensitive to the interventions we propose. However, the relationship of anti-hemozoin and anti-GPI immunity to pathogenesis and clinical immunity in human malaria has not been adequately investigated. **(Amended 11 July, 2005)**

Cell-mediated responses - cytokines: There is less hard evidence on the importance of cell-mediated responses in the development of clinical immunity to malaria in humans under conditions of natural exposure. This may in part be due to technical difficulties associated with measuring responses to multiple parameters with very limited sample volumes. There have been some studies in humans looking at the relevance of cytokine responses in acquired immunity [36] and severity of malaria disease, in particular IFN-γ [37], TNF-α [38, 39] but also other cytokines including IL-4 [40] IL-10 [41, 42, 43], IL-12 [44], and TGF-β [45]. Due to the complexity of Pf infection and the network of immune responses in the host, it is likely that a combination of cytokine mediators is involved in protection. The availability of new methods that allow for the measurement of multiple immune mediators in small volumes of sample now makes it feasible to simultaneously measure multiple cytokines in infant populations.

Influence of host genetics on NAI: ***Host genetic factors are likely to make an important contribution to determining whether an individual infant becomes clinically infected with malaria. Since host genetics may contribute substantially to host defence, understanding the nature of the genetic variations that enhance resistance to malaria and the mechanisms by which this occurs would be helpful to improving the overall understanding of the natural history of malaria infection.***
Host genetic factors likely to be important in determining NAI towards malaria in early life can be categorized into three distinct groups:

(i) Known hematological genetic factors (including sickle cell (HgbS) [84], Hgb AF and α-thalassemia [85]);
(ii) Variations in candidate genes known to be important in inflammatory or immunological responses to infection with malaria (including: ICAM-1 [98]; CD36 [99, 100]; iNOS [101]; complement receptor 1 (CR1) [102]; mannose-binding protein (MBP) [103]; and TNFα [104]).
(iii) Genetic polymorphisms in Th1/Th2 immunological pathway genes. In early infancy, the immune system undergoes a period of rapid change from a predominantly Th2 to a more mature Th1 orientated pattern of cytokine responses [105]. The level of antibodies produced and the specific cellular responses generated at this time are highly dependent on the rate that this transition occurs within the host [105]. In addition, antigens initially provoke a Th2 pattern of responses before a more sustained Th1 response follows and this is likely to be true for the production of all new IgG antibodies in early life [106]. Cellular immunity to malaria related to Th1/Th2 responses is also likely to be important. Hence, investigation of host genetic factors should include a careful examination of significant polymorphisms in genes in the Th1/Th2 pathway. In isolated recent studies, variations in Th1 or Th2 genes such as IL-4 [107], IL-13 [108], IL-12B [109], IL-10 [110], or IFNγR [111], have been associated with the severity of infection with malaria.

The genetic makeup of an individual undoubtedly plays a part in determining their response to anti-malaria treatment and may also influence the age at which individuals respond. In addition, it may be adequate to evaluate the genetic factors of the mother, to assess the potential of maternal influences on the development of the immune system in offspring and other outcomes. **(Amended 10 August, 2005)**
Outcomes relevant to these host genetic studies include both intermediate (those of relevance to the disease) and clinical (the disease itself) phenotypes. Intermediate phenotypes include specific antibodies, plasma cytokine levels and in vitro cellular responses (to provocation with both specific antigens and non-specific agents) (see Section D, viii, 2 a,b) and clinical phenotypes including both primary and secondary clinical outcomes (see Section D, viii, 1 a,b). Incorporation of a comprehensive examination of host genetic factors into this study should provide new insight into mechanisms of NAI to malaria and this knowledge should assist in planning more efficacious future therapies for malaria.

Impact of oxidative stress in the development of NAI: ***During malaria, both host and parasite are under oxidative stress. The oxidative status of immune cells has been shown to influence the quality of the immune response. For example, the redox status of macrophages influences the pattern of cytokines released through the modulation of their glutathione (GSH) content, and the maturation of dendritic cells is inhibited by the pro-oxidant effects of haemozoin. In addition, malnutrition is very common in malaria endemic areas, and affects negatively the***

antioxidant potential in the host. The anti-oxidant/pro-oxidant balance, therefore, is likely to influence the quality and quantity of the immune responses against P. falciparum, but the role of these phenomena in the development of protective immunity has not been established in clinical studies.

(Amended 11 July, 2005)

WHY IS THE IMMUNOLOGICAL RESPONSE OF INFANTS IMPORTANT? In many areas of sub-Saharan Africa, the most significant morbidity and mortality due to malaria occur in African children between 6-12 months of age [46, 2]. Thus, infants less than 6 months of age are a key target population for any interventions against malaria, including vaccines and chemoprophylaxis. However, little is know about the acquisition of immune responses to Pf infection during the first two years of life, and it is not clear how these responses may interface with any malaria preventive intervention. There is evidence to suggest that immune responses in the newborn are suboptimal but that there is a potential for enhancement of these responses [47]. With regard to vaccine studies in infants, in the trials of SPf66 discussed above, where 31% efficacy was demonstrated in older children but 2% efficacy in infants [3, 4, 48], the breadth, intensity and longevity of antibody responses against SPf66 were significantly lower in young infants as compared to older children or adults [49]. As rationalized above (see section on Implications of Proposed Study), suboptimal responses to Pf antigens in the first few months of life will significantly impact infant immunization strategies, with regard to both timing of immunization as well as the type of immunization.

INNOVATIVE NATURE OF STUDY DESIGN: The study design proposed herein is novel in that, for the first time, the period of exposure to malaria is independently controlled during the first year of life by the use of continuous (as opposed to intermittent) chemoprophylaxis, allowing an assessment of the intrinsic effects of two age windows (0 to 5, 5 to 10 months) on the acquisition of NAI. At the core of this protocol is a close prospective follow-up of each infant for two years, utilizing a cross-over design that should provide the opportunity to address, with precision, questions that have not been possible to address with less controlled surveys.

<u>B) PRELIMINARY STUDIES conducted by the Centre de Salut Internacional and the CISM</u>

a) Anti-malarial prophylaxis studies in infant cohorts

Drs. Alonso and Menendez have previously established capabilities for conducting studies in which prophylaxis was administered to large cohorts of newborns in Tanzania [1, 2]. In the first study, conducted between 1995 and 1997, 832 infants born at one hospital in the town of Ifakara, Kilombero District, in south-eastern Tanzania were randomly assigned to receive either of four regimens which included either daily iron, weekly Deltaprim®, both medications, or no active drug; all administrations were placebo-controlled [1]. Daily iron was given from 8 to 24 weeks of age and weekly Deltaprim® was given from 8 to 48 weeks, ingestion of the anti-malarial drug was observed. Infants were successfully kept on these regimens up to 48 weeks of age and followed until 96 weeks of age by a combination of passive case detection and cross-sectional surveys for clinical malaria episodes. In the second prophylaxis study, an intermittent prophylactic treatment trial, carried out in the same site between 1999 and 2001, 701 infants were randomly assigned to receive a single dose of either SP or placebo at 2, 3, and 9 months of age, given according to the EPI schedule [2]. Infants were followed for clinical malaria episodes until 2 years of age by a combination of passive case detection and cross-sectional surveys conducted at 12 and 18 months of age.

Another ongoing large intermittent prophylactic treatment trial in infants started in September 2002 in Manhiça. The design is similar to the one conducted in Tanzania, where infants were randomly assigned to receive either SP or placebo through the EPI services and will be followed up to 18 months of age. In addition, this trial includes immunological studies to evaluate immunity to malaria. These three large-scale intervention studies demonstrate the capability of our proposed group of investigators to conduct studies in newborn and infant populations.

b) Vaccine studies in infants

Dr. Alonso was the senior investigator on the study of the Spf66 vaccine administered to infants alongside the EPI schedule in Tanzania. In this study, 1207 infants were recruited into a two-arm, double-blind, individually randomized placebo-controlled trial conducted within the framework of the EPI. In this first assessment of a malaria vaccine administered to infants, the vaccine was shown to be safe [50] but lacked any efficacy [4]. Immunogenicity studies were performed on a subset of the infants with samples collected at four time points [49]. Antibody responses to the standard EPI vaccines and to SPf66 $(NANP)_{50}$ were measured by ELISA, and antibodies to whole Pf parasite were assessed by IFAT, in collaboration with the Swiss Tropical Institute, Basel, Switzerland.
Dr Alonso was the principal investigator on the RTS,S phase I and phase IIb vaccine trials conducted in Manhiça in 2002-2004, and Dr Guinovart was the project manager of these studies. Immunogenicity studies were also performed at different time points during these trials, including IFAT for anti-parasite antibodies.
These studies establish the capacity of our investigators to conduct all the steps necessary for carrying out immunological studies from field samples: collection from a large cohort of infants, transport of immunological samples to the research site, proper sample storage, and the coordination and conduct of immunological assays.

c) Establishment of the Demographic Surveillance System (DSS) in the Manhiça study area

A continuous DSS has been in place in Manhiça since mid 1996 covering approximately 60 000 inhabitants. The DSS provides a demographic platform that supports the CISM research infrastructure, including description of the health profile of the inhabitants of the district; description of the epidemiology, morbidity, and mortality associated with malaria; and the creation of a platform to assist in the facilitation of new control strategies. Since it was established, with the foundation provided by the continuous DSS, the CISM has undertaken a large number of studies, primarily focused on malaria and acute respiratory infections. Studies related to malaria have been in the following areas: entomology, malaria and pregnancy, anti-malarial combination therapy and drug resistance (ie. chloroquine, SP and amodiaquine), clinical presentation of malaria infection in the pediatric population (< 10 year olds and infants), health seeking behavior, bednet use, intermittent preventive treatment in infants (IPTi) and RTS,S malaria vaccine trials in children aged 1 to 4 years [51, 52, 53, 54, 55]. The DSS has been extremely instrumental in the success of the field studies carried out by CISM and it is anticipated that the accumulated field experience and data collected thus far, particularly on age-specific malaria associated morbidity and mortality, will play a key role in the success of the proposed project.

d) Epidemiologic and clinical studies in infants

(1) Town of Manhiça: The pediatric cohort studies conducted in the Manhiça District since 1997 provide valuable epidemiological data and also establish the capacity of the CISM staff to conduct field work in this location. Between January 1997 and July 2000, an age-stratified study was conducted in Manhiça District, Mozambique (F. Saute, unpublished). A total of 1,071 children less than 1 year of age and 895 children between 1-10 years of age were enrolled and were followed by active case detection with a weekly home visit. At each visit, an axillary temperature was taken by

electronic digital thermometer and caretakers were asked whether there was any history of fever in the prior 24 hour period. In the case of a history of fever in the 24 hours prior to the visit or of a documented axillary temperature of 37.5 ºC, a blood sample was taken by finger prick and processed for two parasite smears. Upon detection of parasitemia, the children in this study were referred to seek medical care at the Manhiça Hospital. See Section D for details of results. We intend to use the same methods of active case detection in our proposed study.

(2) Ilha Josina~~, site of our proposed study~~: A cross-sectional study of 438 children aged 6 to 18 months has been carried out in Ilha Josina, located approximately 50 km from Manhiça. An initial cross sectional sampling during one week in the dry season (19/07/2001 to 27/07/2001) was performed to measure the prevalence of parasitemia and anemia. Samples included a blood slide, hemoglobin and ***Haematocrit (***Hct***)***, serum sample, and a malaria quick-test® (based on anti-histidine rich protein antibodies) for parasitemia, and axillary temperature and weight were determined. A questionnaire prior to the blood draw also recorded data regarding socio-economic and behavioral characteristics of the children and their families (see Section D for details of results).
Children in one of the two cohorts of the phase IIb RTS,S malaria vaccine trial conducted in 2003-2004 by CISM were recruited from Ilha Josina. These 416 children were followed by passive case detection at the Ilha Josina Health Post and by active case detection after they received dose 3 of the vaccine. Active case detection was carried out by fortnightly home visits during the first 2 and a half months and monthly visits during the following two months. During the visits a brief morbidity questionnaire was administered, the axillary temperature was measured and a finger-prick blood sample was taken to prepare a blood slide.
These cohort studies in infants and young children establish the capacity of the CISM investigators to coordinate and complete such studies ~~at the proposed study site~~. **(Amended 15 October, 2004) (Amended 2 December, 2004)**

e) Immunological studies
Laboratory facilities at the CISM comprise a basic parasitology and haematology lab including a coulter counter for full haematological analysis and equipment for dry biochemistry tests. There is a fully equipped immunology lab that includes water purification systems, liquid nitrogen containers, a 4-Color FACSCalibur flow cytometer, fluorescent microscope, ELISA capabilities, standard molecular biology and sterile cell culture facilities. A tissue paraffin processor for histological samples is also available. The geographical location of the CISM near South Africa allows for regular shipping and maintenance of equipment
Currently, there are 3 ongoing intervention trials:at the CISM a phase IIb RTS,S malaria vaccine trial, a trial of intermittent preventive treatment in infants (IPTi), and a trial of combination of intermittent preventive treatment and insecticide treated nets in pregnant women. As part of these studies, immunological studies are being conducted to evaluate the impact of malaria control strategies on the development of naturally acquired immune responses. This assessment includes a set of immunological and parasitological assays that are routinely performed at the CISM laboratories: culture of *P. falciparum* isolates, standard cell culture, intracellular cytokine staining, flow cytometry, ELISA, indirect immunofluorescence assay, and PCR genotyping of parasites. In addition, the CSI laboratory at the Hospital Clínic in Barcelona is equipped with a Luminex machine with capabilities for measuring cytokine and antibody responses in a multiplex manner with low volumes of sample. The experience in these studies and the facilities available at the CISM and CSI laboratories prove the capacity of the CISM investigators to undertake the immunological studies included in this proposal.

C) SPECIFIC AIMS

<u>Primary Aim:</u> Determine whether exposure to Pf erythrocytic stage antigens between 0 to 5 months of age, or between 5 to 10 months of age, affects the subsequent risk of clinical malaria between 12 and 24 months of age, as compared to infants with continuous exposure.

<u>Secondary Aims:</u>

1) **Determine whether exposure to Pf erythrocytic stage antigens between 0 to 5 months of age, or between 5 to 10 months of age, affects the type and quality of immune responses.**
2) ***Evaluate the role of oxidative stress measured as the antioxidant / pro-oxidant balance in the development of NAI in children exposed to Pf erythrocytic stage antigens between 0 to 5 months of age, or between 5 to 10 months of age, compared to infants with continuous exposure.***
3) ***To study the host genetic factors that influence the development of NAI to malaria in early life.***

(Amended 10 August, 2005)

POTENTIAL IMPLICATION OF THE RESEARCH

The most important implication of this work is with regard to vaccine administration or administration of other strategies to prevent malaria and the design of trials. If our results indicate that exposure prior to 5 months of age does not contribute to the development of protective immune responses against *P. falciparum*, it will be necessary to address the optimal time during infancy for immunizing with a vaccine designed to induce such protective immune responses. In many areas of sub-Saharan Africa, malaria immunization alongside the EPI schedule (i.e. 6, 10, and 14 weeks) has been viewed as the most desirable approach because of issues of compliance and funding, and in order to generate protective immune responses prior to 6 months of age, when morbidity and mortality drastically increase. The studies proposed here should provide critical information regarding whether vaccination according to the EPI schedule or implementation of of other malaria-control strategies could be expected to result in adequate induction of protective immunity in infants and children or whether immunization or implementation later in infancy may be more appropriate.

HOW THE PROPOSED STUDY DESIGN ADDRESSES THE AIMS

The proposed study is designed as a double-blind randomized placebo-controlled trial. Newborn infants born to HIV-negative women from ~~Ilha Josina~~ ***Maragra or Manhiça***, Mozambique, an area with stable malaria transmission, will be randomized to one of three cohorts (~~77~~ ***98*** infants per cohort). Cohort 1 will receive monthly chemoprophylaxis with sulfadoxine-pyrimethamine (SP) + Artesunate (AS) from ~~2 until 5~~ ***2,5 until 5,5*** months of age (doses administered at ~~2, 3, and 4~~ ***2,5; 3,5; 4,5*** months) and placebo from ~~5 to 10~~ ***5,5 to 10,5*** months of age; Cohort 2 will receive placebo from ~~2 until 5~~ ***2,5 until 5,5*** months of age and monthly chemoprophylaxis from ~~5-10~~ ***5,5 to 10,5*** months of age (doses administered at ~~5, 6, 7, 8, and 9~~ ***5,5; 6,5; 7,5; 8,5; 9,5*** months); and Cohort 3 will receive monthly placebo from ~~2 to 10~~ ***2,5 to 10,5*** months of age. We have chosen SP+AS because it was shown to have a 100% clinical efficacy at day 14 of follow up and a parasitological cure of 94% on day 7, without reappearing on day 14, during an antimalarial efficacy trial conducted recently in Manhiça [89]. The combination of SP+AS is not currently used in the study area, as the national first line treatment is SP+Amodiaquine (AQ), thus the efficacy of SP+AS is expected to be higher than that of SP+AQ, which has already been used for some time on a wide scale. We have chosen to start SP+AS at 2 months rather than at birth because the use of SP as an anti-malarial is relatively contraindicated in infants less than 2 months of age due to the risk of kernicterus. Furthermore, previous data from our study site indicates that there is essentially no malaria in the first two months

of life, presumably due to protection afforded by maternal antibodies. Following 2 months, the age-specific incidence of clinical episodes of malaria increases to levels near that of the second year of life (see Section D, Study Site). Thus cohort 1 should be essentially free of parasitemia during the first 5 months of age, cohort 2 will be exposed to Pf parasites until 5 months of age, and then not exposed until approximately 10 months and cohort 3 will have continuous exposure. Since SP and AS does not affect pre-erythrocytic stage exposure, all children will have pre-erythrocytic stage exposure continuously. Therefore, it is the degree of exposure to erythrocytic stage antigens that is the independent variable manipulated by the intervention. ***We have chosen to administer the chemoprohylaxis at 2,5 – 10,5 months and not at 2-10 months of age to avoid possible interactions between the SP+AS treatment and the responses to the EPI vaccines. The SP+AS treatment will be administered 15 days prior to the EPI vaccines (which are administered at 0, 2, 3, 4 and 9 months of age), a sufficient time lag to avoid such interactions.*** **(Amended 15 October, 2004) (Amended 2 December, 2004) (Amended 10 August, 2005)**

For our primary outcome variable, we will compare the risk of having at least one malaria episode ***as detected by passive case detection*** between 12-24 months in the three cohorts. We have chosen our primary outcome variable and follow-up period (12-24 months) in order to duplicate the parameters from the two previous clinical trials upon which this study is based [1, 2]. We anticipate that Cohort 2, with exposure to blood stage antigens blocked during the latter half of infancy, will have a statistically significantly higher risk of at least one episode of malaria between 12-24 months of age than will Cohorts 1 and 3, and that by the same measure, Cohorts 1 and 3 will be similar to each other in terms of risk. Because we are aware that an affect on clinical outcome could be seen even after year two, we will also include follow-up during year three consisting of home visits every 3 months. Children will continue to have full access to both the ~~Ilha Josina~~ ***Maragra*** Health Center and Manhiça Hospital for care. In addition, children will be followed long-term for mortality through the demographic surveillance system (DSS) already in place. **(Amended 15 October, 2004) (Amended 2 December, 2004)**

We also propose to obtain samples for both antibody and cell mediated immunology studies at multiple time points during the first two years of life ~~(0, 5, 10, 12, 15, 20 and 25 months of age)~~ ***(~~0;~~ 2,5; 5,5; 10,5; 15 and 24 months of age)*** from each cohort to (i) describe the acquisition of immune responses with age and exposure, (ii) compare the type and quality of immune responses between infants from the three different groups and (iii) explore the association between any measured immune response and the prospective risk of malaria in the second year of life. **(Amended 2 December, 2004) (Amended 11 July, 2005)**

In addition, host genetic factors associated with favorable intermediate or clinical malaria outcomes will be examined with respect to the timing of exposure to malarial parasites. **(Amended 11 July, 2005)**

D) RESEARCH DESIGN AND METHODS

i. Study Site and Population Characteristics

Study site: The study will be conducted in the ~~village~~ ***neighborhood*** of ~~Ilha Josina~~ ***Maragra***, ~~located 40 km north of~~ ***in*** Manhiça, ***and in Manhiça,*** in the District of Manhiça (Maputo Province) in Southern Mozambique (25º 24' south latitude, 32º 48' east longitude). Residents of ~~Ilha Josina~~ ***Maragra and Manhiça*** are included in the area monitored by the DSS which has been in place since mid 1996

encompassing approximately 60 000 inhabitants, and which greatly facilitates the execution of studies in the area. Each home in the area is visited twice a year by field workers who track data such as number residing in the home, births, deaths, and relocations. The ~~Ilha Josina~~ ***Maragra*** health post is ***used by the surrounding population*** ~~the only health facility in the area~~***, and*** it includes an outpatients department for adults and children with a basic laboratory to determine parasitaemia and haematocrit and a maternity and EPI services. ***The Manhiça hospital is the referral district hospital, where the patients from the Maragra health post are transferred when needed.*** CISM runs a morbidity surveillance system at the health post that is identical to that running at the Manhiça hospital. A standardized questionnaire is filled out for each child attending the outpatients department, where the sociodemographic characteristics and the signs and symptoms presented by the child are recorded. **(Amended 15 October, 2004) (Amended 10 August, 2005)**

Population characterisitics: People of the area are mainly Xironga and Xichangana and their languages are often termed as Ronga and Changana. The people are mostly subsistence farmers living in compounds separated by garden plots and grazing land. Houses are simple, with walls typically made of cane with thatched or corrugate roofs. Illiteracy is high (24% of male and 47% of female). Approximately 66% of men and 49% of women have primary education; 9% of men and 4% of women have secondary education; and less than 1% of both men and women have gone beyond their secondary education. Socio-economic status is unlikely to be a confounding factor for our study since all individuals reside in the same location and have very similar standards of living. There are ~~approximately 1,445 newborns per year in the entire region covered by the DSS and~~ approximately 400 newborns per year ~~in~~ ***born at*** the ***Maragra health post maternity*** ~~Ilha Josina study region~~. The infant mortality rate is 78.5 per 1,000 live births. The mortality rate in children under 5 years of age is 130 per 1,000 children. Acute respiratory illnesses and malnutrition are the most important causes of disease and death in children under 5 in this region Although there are two distinct seasons in this region; a warm season between November and April when most of the rains concentrate (annual rainfall during 1998 was 1,100 mm) and a cool and dry season during the rest of the year. **(Amended 15 October, 2004)**

Malaria-related indices: Malaria transmission in ~~Ilha Josina~~ ***Maragra and Manhiça*** is stable. ~~Although average entomologic inoculation rate (EIR) for Ilha Josina are not yet available, the EIR in nearby~~***. The average entomologic inoculation rate (EIR) for*** Manhiça is estimated at ~~15~~ ***38*** infective bites per person per year ~~(Aranda C *et al*, unpublished)~~. ~~CISM investigators are in the process of determining the EIR in Ilha Josina and preliminary data indicates it will be much greater than that in Manhiça.~~ **(Amended 15 October, 2004) (Amended 10 August, 2005)**

Malaria morbidity and mortality data: A survey conducted at the Manhiça Hospital indicated that malaria alone accounted for half of the outpatient consultations and admissions. The overall mortality rate in the pediatric ward was 4%. Although malaria had a relatively low case fatality rate (2,3%), it accounted for half of all deaths. The rest of mortality was accounted for by nutritional disorders, lower respiratory tract infections, meningitis, and septicemia. Of note, a recent screening at Manhiça Hospital of approximately 350 samples from healthy pregnant women and from children documented to have anemia have failed to show any evidence of Haemoglobin S in the population.

HIV Voluntary Counseling and Testing Center (VCT center) and HIV infection data: HIV infection is becoming an increasing problem in the area. A VCT center is set up at the Manhiça Hospital ***and at the Maragra Health Post*** for ~~pregnant women~~ ***individuals*** who wish to get tested for HIV. They receive counseling by a trained counselor and are ***tested for HIV. All testing is confidential and results are available a few minutes after testing. All HIV positive clients are referred to Day***

Hospitals, community services for continued clinical and psychological support. HIV positive people are enrolled in the Health system anonymously, followed only by their unique code number. If patients meet the national criteria and the compliance requirements for initiating HAART (highly active antiretroviral therapy) they are included to receive HAART.
Pregnant women who are HIV-positive are currently offered to receive treatment with Nevirapine (NVP) during delivery, to decrease the risk of vertical HIV transmission. ***However HAART will now be implemented through Day Hospitals, and the recommendation is to administer Combivir (3TC and AZT) to pregnant HIV-positive women starting the 32nd week of gestation throughout the 1st week after delivery. A single dose of NVP is administered to the mother prior to labor/delivery and to the infant within 72 hours after birth. If the women has CD4<350 and meets adherence criteria for HAART, these women will not receive NVP during labour but the infant will receive the dose of NVP within 72 hours after birth.*** The prevalence of HIV infection among the pregnant women who attend the VCT center in Manhiça is 20%. ***Guidelines regarding available HIV/AIDS treatment in Mozambique are constantly evolving. The current protocol will take into account changes and integrate national guidelines modifications and administer new treatments for adults and children as they are approved by the Mozambican government.*** ~~Although there are no data from Ilha Josina, as there is not a VCT center, we expect a similar or lower prevalence. For the purposes of the study a counselor from the Manhiça VCT center will displace to Ilha Josina at least once a week to attend the pregnant women in the area who wish to get tested. If necessary the pregnant women will be referred to Manhiça hospital.~~ **(Amended 15 October, 2004) (Amended 2 December, 2004)**

Pediatric malaria episodes in the town of Manhiça: An age-stratified study was carried out in Manhiça in 1997 (F. Saute, in press) in a total of 1,071 children less than 1 year of age and 895 children between 1-10 years of age who were followed by active case detection with a weekly home visit. The highest incidences of malaria episodes (fever plus any degree of parasitemia) were observed between October and April (rainy season). Infants experienced the highest attack-rate, which decreased steadily thereafter with increasing age. In infancy, the highest attack rates (0.7 -0.9 attacks per child per 100 person-weeks-at risk [PWR]) were observed between the ages of 6-11 months of age. There were no clinical cases in the infants 0-2 months of age. The risk of malaria appeared to rise rather dramatically after about the age of 2 months. In the older children, the peak incidences were observed in children 1-4 years of age (0.7-0.8 attacks per 100 PWR).

INCIDENCE OF MALARIA EPISODES BY AGE IN MANHICA, MOZAMBIQUE

Age group	Episodes	PWAR	Rate per 100 PWAR	95% CI	
0 < 2m	0	1130	0	.	.
2m < 6m	35	9491	0.4	0.3	0.5
6m < 8m	46	6134	0.7	0.6	1
8m < 1y	117	12963	0.9	0.8	1.1
1y < 2y	61	8913	0.7	0.5	0.9
2y < 3y	104	12465	0.8	0.7	1
3y < 4y	83	11484	0.7	0.6	0.9
4y < 5y	51	10003	0.5	0.4	0.7
5y < 6y	49	8773	0.6	0.4	0.7

**6y < 7y**	**39**	**6429**	**0.6**	**0.4**	**0.8**
**7y < 8y**	**7**	**4129**	**0.2**	**0.1**	**0.4**
**8y < 9y**	**14**	**3914**	**0.4**	**0.2**	**0.6**
**9y < 10y**	**13**	**3754**	**0.3**	**0.2**	**0.6**
**10y < 11y**	**7**	**2880**	**0.2**	**0.1**	**0.5**

PWAR: Persons Week at risk
Chi-squared test for unequal rates = 25630.2 (13 df, p = 0.000)
Chi-squared test for linear trend= 15.7 (1 df, p = 0.000)

~~**Pediatric malaria episodes in Ilha Josina (our study site):** Data from a prospective study in 438 children enrolled between the ages of 6 and 18 months (described in Section B, Preliminary Studies) indicated that **87%** of the children were positive for malaria at a single blood draw performed during the dry season; 97% were infected with *P. falciparum,* 1.2 % were infected with *P. malariae* and 0.4% were infected with *P. ovale*. The rest (1.2%) were mixed infections. The average parasite density in the positive samples was 4,277 parasites/µl. 38% of the 240 samples with parasitemia came from children who had fever (axillary temperature ≥ 37.5º C).~~

~~***Hematologic indices:*** the initial cross-sampling survey of children 6-18 months (dry season) indicated that for 290 children, 19% (55) had a hematocrit (Hct) of ≥ 33% (no anemia); 64% (185) had a Hct between 25% and 33% (moderate anemia); 35 children (12%) had a Hct between 15% and 25% (severe anemia); and 15 children (5%) had a Hct < 15% (very severe anemia).~~
~~***Parasitemia:*** All infections during the entire follow-up period (4 samples, each sample collected two weeks apart) were with *P. falciparum.* Incidences for children aged 12-18 months (n = 92):~~

~~- 61% of the 92 children had at least one episode of parasitemia with or without fever during the 2 month follow-up period (the 8 weeks when the 4 cross-samplings were done).~~
~~- The incidence of parasitemia with or without fever was estimated as 3.3 episodes per child per year.~~

~~***Clinical episodes*:**~~

~~- 10 of 92 children between 12 and 18 months had at least one episode of clinical malaria (fever documented as axillary temperature ≥ 37.5º C plus parasitemia of any density) during the 2 month follow-up period.~~
~~- The incidence of clinical episodes was estimated as 0.48 episodes per child per year.~~

(Amended 15 October, 2004)

ii. Recruitment and Enrollment

In order to meet our calculated total sample size of ***117*** ~~***98***~~ ~~77~~ infants per cohort (see section v below) ***we will recruit a) infants aged less than 2 months, identified in the community, born from HIV-negative mothers and b) HIV-negative pregnant women, identified when they attend the VCT center at Maragra or Manhiça, to subsequently enroll their newborn infants.*** **(Amended 2 December, 2004) (Amended 10 August, 2005) (Amended 12 September, 2006)**

a) Infants aged less than 2 months

A list of infants aged less than 2 months, whose mothers are alive, living in Maragra will be produced from the census database. The mothers of these infants will be visited at home by a field worker and will be invited to participate in the study. They will be informed that prior to participating it is necessary to attend the mother and child health clinic (MCH) at Maragra to

check that both mother and infant are healthy. At the MCH clinic the infant will be weighed and measured and his/her road-to-health card will be checked to make sure the infant has received the EPI vaccines at birth. If he/she has not received them they will be administered to him/her. Afterwards, the mother will be informed that if she is interested in participating in the study she has to attend the VCT center first, as only infants of HIV-negative mothers are eligible to participate.
If she has never attended the VCT center before and is interested in getting tested for HIV she will be invited to attend the VCT center at the antenatal clinic. At the VCT center, a trained counselor will tell her about the study and will ask her to sign an informed consent to accept being tested for HIV (see details of informed consent process below). After the informed consent has been signed, the mother will undergo all standard procedures, which include counseling and testing for HIV by a trained counselor, ensuring confidentiality. If the woman is HIV-negative and is interested to participate, the counselor will again tell her about the risks and benefits of the study and will then ask her to sign a second informed consent, where she agrees to enroll her infant in the study. If the woman is HIV-positive the counselor will explain that she cannot participate in the study but will counsel her and refer her to the Day Hospital to receive standard treatment, according to national guidelines (see above).
If the mother had already attended the VCT center during the last three months of pregnancy and is interested in participating in the study she will also be invited to attend the VCT center. At the VCT center she will be told about the study and will be asked to sign the first informed consent, where she agrees to disclose the results of her previous HIV test. If the woman is HIV-negative and is interested to participate, the counselor will again tell her about the risks and benefits of the study and will then ask her to sign a second informed consent, where she agrees to enroll her infant in the study. If the woman is HIV-positive the counselor will explain that she cannot participate and she will continue the standard treatment.
(Amended 2 December, 2004)

b) Pregnant women

~~we~~ ***We*** will enroll ~~306~~ ***~~366~~*** HIV-negative pregnant women, under the assumption that the infants of approximately ~~25%~~ ***20%*** of these women will not be included, either because of drop-out of the mothers, or because the infant is not full-term and/or healthy at birth. Pregnant women will be identified ***when they attend the VCT center at Maragra or Manhiça.*** ~~in the community.~~ Only women who have attended the VCT center and are HIV-negative will be invited to participate. At the VCT center women will undergo the standard procedures of the VCT center, which include counseling by a trained counselor before and after performing the test. If the woman is HIV-negative and is interested to participate, she will be told about the risks and benefits of the study ***by the counselor*** and will then sign an informed consent. (A thumbprint may be used if the mother is illiterate; additional details of the informed consent process are defined below).~~ If the mother is HIV-positive she will not be invited to participate in the study, and will be followed up at the VCT center following standard procedures.~~
Each enrolled pregnant woman will be followed by a field worker ~~residing in Ilha Josina~~. Women will be asked to give birth at the maternity if at all possible. ***We anticipate that most*** ~~Some~~ deliveries will take place at the maternity of ~~Ilha Josina~~ ***Maragra or Manhiça*** while ~~some~~ ***only a small percentage*** are expected to be home deliveries. Upon delivery of a healthy, full-term infant, we will again discuss the study with the mother if she still agrees to participate. If the delivery takes place at the maternity an assigned health care worker will collect a blood sample from the mother~~, from the infant,~~ and from the cord as well as a small sample of placental tissue, as detailed in section vii. If the delivery takes place at home it will be attempted to keep the placenta, but no blood samples from the placenta or the cord will be collected. A few days later a blood sample will be taken from the mother ~~and the child~~.

If the pregnant woman/mother is HIV-positive she will not be invited to participate in the study, and will be followed up at the Day Hospital following standard procedures, according to national guidelines, which includes HAART (see above for details).
The investigators will not have access to any of the VCT center records and will receive no information at all on the HIV-status of women who are not enrolled in the study.

(Amended 15 October, 2004) (Amended 2 December, 2004) (Amended 11 July, 2005) (Amended 10 August, 2005)

iii. Experimental Cohorts
Infants who meet the criteria to participate in the study will be assigned to one of the 3 cohorts by block randomization, in order to adjust for seasonality of malaria infection. Thus, for every 6 infants, 2 will be randomized to each of the 3 cohorts. Enrollment of infants will continue over a one year period. Cohort 1 will receive chemoprophylaxis with SP+AS from ~~2 to 5~~ ***2,5 to 5,5*** months of age (doses at months ~~2, 3 & 4~~ ***2,5; 3,5; 4,5***) and placebo from ~~5 to 10~~ ***5,5 to 10,5*** months of age. Cohort 2 will receive placebo from ~~2 to 5~~ ***2,5 to 5,5*** months of age and chemoprophylaxis from ~~5 -10~~ ***5,5 to 10,5*** months of age (doses at months ~~5, 6, 7, 8 & 9~~ ***5,5; 6,5; 7,5; 8,5; 9,5***), and Cohort 3 will receive placebo from ~~2 to 10~~ ***2,5 to 10,5*** months of age. Placebo tablets will look exactly the same as the SP and AS tablets, with the same shape, size and colour. The treatment or placebo will be packed in individually numbered envelopes/bags. Doses of SP+AS/placebo will be administered on a monthly basis by a health assistant according to bodyweight (SP: single dose of 25 mg/kg Sulphadoxine and 1.25 mg/kg Pyrimethamine; AS: 4 mg/kg/day for three days). Tablets will be crushed and mixed with water on a tablespoon and administration will be directly observed. Infants will be observed for 30 minutes and a repeat dose will be given if vomiting occurs within that time. ***Infants from all three cohorts will be invited to the health post on month 2, 3, 4 and 9 to receive the EPI vaccines and the investigators will make sure they receive them.*** Subjects will be followed by active case detection as well as passive case detection until ***10,5 months of age and by passive case detection and montly home visits from 10,5 to*** ~~25~~ ***24*** months of age (see section v). **(Amended 2 December, 2004) (Amended 11 July, 2005)**

Rationale for the 3 cohorts:
The three cohorts are proposed in order to address the **central hypothesis:** Exposure to Pf erythrocytic stage antigens in early infancy does not contribute to the development of naturally acquired immunity to malaria but exposure after 5 months of age does contribute to the development of naturally acquired immunity. Specifically, the three cohorts are needed to address the following:

1- The comparison of Cohort 1 (prophylaxis ~~2-5~~ *2,5-5,5* months) and Cohort 2 (prophylaxis ~~5-10~~ *5,5-10,5* months) is the key comparison. During the first 10 months of life, we assume that both Cohorts 1 and 2 will have experienced exposure to Pf blood stage parasites, and therefore Pf erythrocytic stage antigens, since each have been left unprotected by prophylaxis during several months. We recognize that infants in the first 5 months of life are likely to have a greater degree of protection due to maternal antibodies than those who are 5-10 months of age, although there is some controversy with regard to this [72]. Nevertheless, previous data from our study site indicate that there is essentially no malaria in the first two months of life but that after 2 months, the age-specific incidence of clinical episodes of malaria increases to levels near that of the second year of life. We also recognize that younger infants may be kept more covered by their mothers and thus experience less exposure to mosquitoes. Thus, the rate of malaria acquisition in the two cohorts may not be the same. Because this will be an uncontrolled variable that may affect the outcome measures of the

study, the effects of the number of episodes of parasitemia will be included in the statistical model used for analysis (see below). **(Amended 2 December, 2004)**

2- By including Cohort 3, we are attempting to replicate the original observations that led to the formulation of the central [1, 2] – that preventing exposure during the first few months of life (Cohort 1) does not modify the rate at which the infants acquire immunity to malaria as compared to the Control group (Cohort 3). We believe it is necessary to include the Cohort 3 given that we are working in an area of different endemicity from the Tanzanian studies. Cohort 3 is needed in order to measure baseline incidence of parasitemia and clinical malaria episodes and furthermore, it is needed to provide control samples for the immunological assays performed on Cohorts 1 and 2.

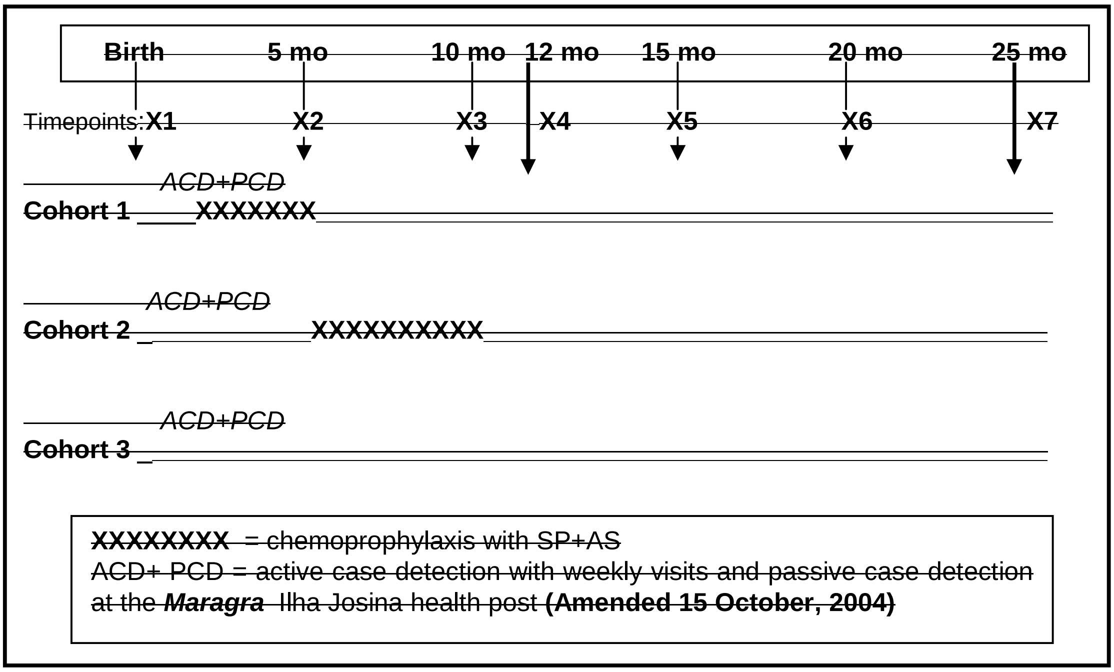

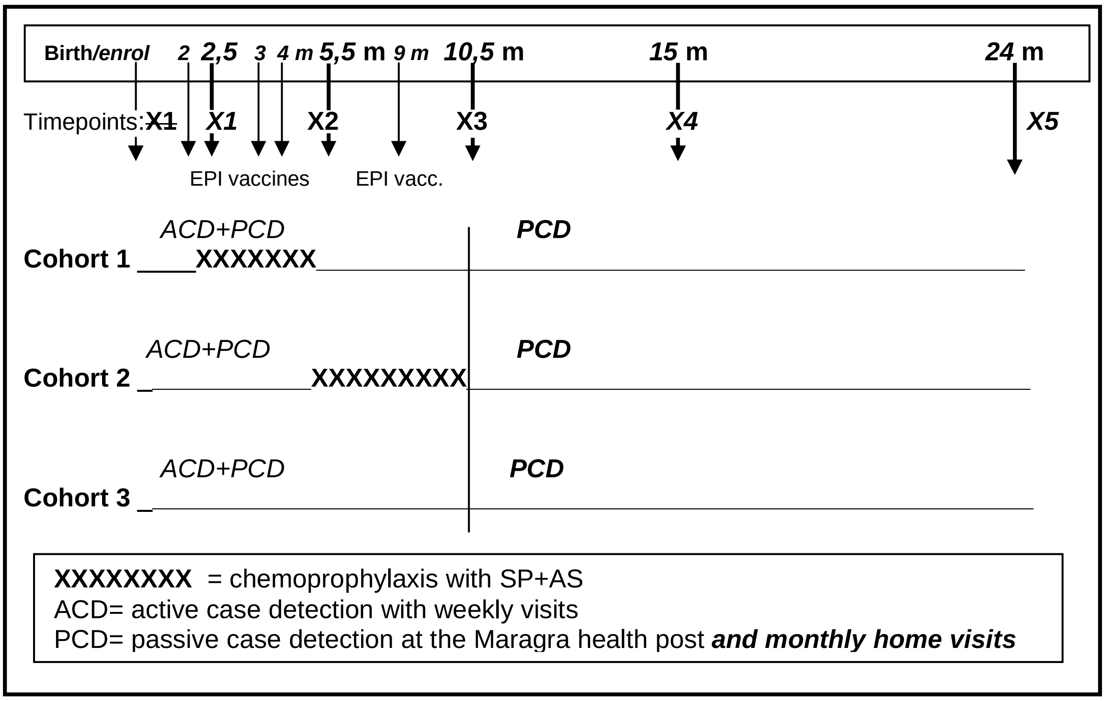

(Amended 2 December, 2004) (Amended 11 July, 2005)

iv. Case Definition and Detection

Case definition: Any fever upon exam (axillary temperature ≥ 37.5^{o} C) or history of fever within the prior 24 hours plus the presence of Pf asexual stage parasites of any density will constitute a malaria episode.

Active case detection: Active case detection will consist of a weekly visit by the field worker to the home of the infant ***from enrollment until 10,5 months of age***. During this visit an axillary temperature will be taken by electronic digital thermometer and the caretaker(s) will be questioned about any history of fever. In the case of a documented fever (axillary temperature ≥ 37.5^{o} C) or history of fever in the previous 24 hours, thick and thin blood smears will be collected, stained, and read according to quality-controlled procedures [9]. If there is no fever or history of fever, no sample will be taken. ***The purpose of this active case detection follow-up is to detect the first encounter with the parasite that produces clinical malaria during the first 10 months of life, the period of the intervention. The respiratory rate of the child will also be recorded, and if increased (>=60 breaths/min in children younger than 2 months and >=50 in children aged 2-12 months), ~~the child will be taken to the health post~~ the mother/guardian will be advised to take the child to the health post if it persists.*** During these weekly visits, the field worker will also inquire as to whether the child has been hospitalized for any reason or if any skin rashes (potentially indicative of adverse reaction to SP) or other signs/symptoms have developed. **(Amended 2 December, 2004) (Amended 11 July, 2005)**

Passive case detection: Caretakers will be asked to bring infants into the ~~Ilha Josina~~ ***Maragra*** health post ***or the Manhiça health center***, where a morbidity surveillance system is functioning, whenever the child is ill. We propose to only enroll families who live within close access to the ~~Ilha Josina~~ ***Maragra or Manhiça*** health post so that they will all have access to the health facility if they wish to seek care for their children. **(Amended 15 October, 2004) (Amended 10 August, 2005)**

Monthy surveillance home visits: Home visits will be performed monthly from month 10.5 to month 24 of age. Parents/guardians will be asked about the health status of the child and whether he/she has been admitted to hospital since the previous visit. These visits will ensure that safety information on serious adverse events and deaths is captured. **(Amended 11 July, 2005)**

Treatment and follow-up: Any child with uncomplicated malaria will be treated as per standard of care in this area, currently with SP and Amodiaquine, the national first line antimalarial treatment. Children who have severe malaria will be transported by study vehicle to the Manhiça Hospital and referred to the intensive care unit for further care. As per standard of care at the Manhiça Hospital, they will have additional admission labs, including a CBC, glucose, blood culture, and lumbar puncture (if indicated) and will be treated with IV quinine and other support as needed, including intravenous hydration, anti-bacterials, or transfusion. After completing therapy, children will be followed up at routine weekly home visits. Those infants who fail therapy with SP plus Amodiaquine will be treated with a second-line treatment. For those children who have failed second line treatment, treatment will be given with oral quinine. One month after the ~~infection~~ ***first episode of malaria***, the child will have another blood draw for immunological studies.
(Amended 11 July, 2005)

v. Study Duration

A child's participation will continue until the child is 25 months of age unless the child is withdrawn, cannot tolerate SP+AS, or the family moves out of the study area. We anticipate a drop-out rate of 15% by the end of the second year and have adjusted our sample size accordingly.

vi. Sample Collection
Field workers will be assigned to conduct the weekly active case detection. They will initially follow the pregnant women and then, upon enrollment of the infants, will be assigned a group of infants from each cohort. In addition, there will be staff available 24 hours a day, 7 days a week at the ~~Ilha Josina~~ ***Maragra*** Health Center ***and Manhiça health center*** to evaluate children who present with fever and/or symptoms of malaria. Microscopy and determination of Hematocrit can be performed at the ~~Ilha Josina~~ ***Maragra*** Health Center. Those samples requiring further work-up will be transported at 4 ºC in coordinated transports from ~~Ilha Josina~~ ***Maragra*** to Manhiça.
(Amended 15 October, 2004) (Amended 10 August, 2005)

The timepoints for collection of samples will be:

1. ~~Birth, 5, 10, 12, 15, 20 and 25 months of age~~ ***Birth ~~or enrolment~~*** - ***Cross sectional cord blood samples*** for parasitological***, ~~host genetics, full blood count~~ full blood count (FBC)*** and immunological studies.

2. 2,5 months of age – Cross sectional samples for ~~haematocrit~~ FBC, filter paper, host genetics and immunological studies.

~~2.~~ 3. 5,5; 10,5 and 15 months of age – Cross sectional samples for immunological studies and FBC. If the child has fever, blood will also be taken to determine parasitaemia and haematocrit.

~~3.~~ 4. 24 months of age – Cross sectional samples for parasitological (blood smear and filter paper), ~~complete blood count~~ ~~haematocrit~~ FBC and immunological studies.

~~2~~ ***~~4.~~ 5.*** Time of first infection and one month later - ***Acute and convalescent*** samples for parasitological ~~and immunological~~ ***and immunological*** studies will be drawn at the time of the first documented clinical episode and one month later.

~~3~~ ***~~5.~~ 6.*** All subsequent clinical episodes until ~~25~~ ***24*** months of age - ***Acute samples*** for parasitological studies only.
(Amended 2 December, 2004) (Amended 11 July, 2005) (Amended 10 August, 2005)

Collection of samples at birth will be as follows:

Maternal: ~~5 ml~~ ***10 mL*** venous blood for ~~Hct~~ ***FBC***, thick and thin blood smear for detection of parasitemia, ~~and~~ ***immunological studies, including*** measurement of maternal antibodies, ***and host genetics of the mother (same genotypes measured in offspring will be measured in the mother)***. In addition, tissue and blood samples of the placenta will be collected [73] to determine whether there is placental sequestration and to evaluate the genotypes of any sequestering parasites. A biopsy of 2.5-cm^2 will be cut from the maternal surface in an off-center position (in an area without evidence of infarction), halfway between the umbilical cord and the edge of the placenta, and placed in 25 ml of 10% neutral buffered formalin. The remaining placental specimens will be kept at 4°C, embedded in paraffin wax using standard techniques, and a 4 µm thick section will be stained with hematoxylin and eosin, or Giemsa and perodic acid-Schiff for standard histological evaluation and quantification of parasites and malaria pigment. The placental blood sample will be collected onto filter paper and used for PCR diagnosis of placental parasitemia and genotyping.

(Amended 10 August, 2005)

Newborns *recluted at birth*: Neonatal blood will be collected from ~~(i) heelprick and (ii)~~ cord blood where possible, to measure the ~~Hct~~ ***FBC***, parasitemia (thick and thin blood smear), and ~~baseline~~ immunology, including levels of maternal antibodies to *P. falciparum*~~, and to carry out host and parasite genotyping~~. Collection of cord blood will allow us to obtain a larger sample volume for ~~genetic and~~ immunological testing, and for archiving purposes. Importantly, it will help us to clarify whether there has been *in utero* infection with *P. falciparum*, since actual exposure is more closely reflected from the cord than from the newborns' peripheral blood (S. Rogerson, personal communication). The umbilical cord will be cleaned thoroughly with saline soaked gauze to wash off the blood on the surface and thus avoid contamination with maternal blood. Blood from fetal circulation in the cord will be collected from inside by venipuncture, using standard techniques. The possibility of materno-fetal transfusion caused by placental trauma at delivery, with its risks of congenital malaria, will be considered if the mother has proven parasitemia in her peripheral blood and/or placenta as documented by positive blood smears or histology, and accounted for in the analyses. ~~In addition to the cord blood, 0.5-1 ml of peripheral blood will be drawn by heel prick within 24 hours of birth, and will serve as the core specimen at t=0 for immunological analysis.~~ **(Amended 11 July, 2005) (Amended 10 August, 2005)**

Infants aged 0-2 months recruited after birth: Blood will be collected at 2,5 months of age from heel or fingerprick to measure the Hct~~, parasitaemia (thick and thin blood smear)~~ and baseline immunology ~~and to carry out host and parasite genotyping~~. **(Amended 2 December, 2004) (Amended 11 July, 2005)**

vii. Data Recorded

1. Clinical and parasitological data

a) Clinical malaria: Episodes of clinical malaria will be recorded from the information collected at weekly home visits or with cross-sampling at ~~5, 10, 12, 15, 20 and 25~~ ***2,5; 5,5; 10,5; 15 and 24*** months if the child meets the case definition for malaria at that time, or if child presents to the health post with malaria. **(Amended 2 December, 2004) (Amended 11 July, 2005)**

b) Parasitemia and *P. falciparum* genotypes: presence of parasites and density of parasitemia. If positive, parasites will be genotyped using the MSP-1 and MSP-2 polymorphic markers (see section xi).

Finger prick blood samples will be obtained at cross-sampling at ~~5, 10, 12, 15, 20 and 25~~ ***24*** months of age, or if the child has fever (axillary temperature ≥ 37.5^{o} C) either at the weekly home visit, at presentation to the health center ***or at cross-sectionals at 2,5; 5,5; 10,5 and 15 months of age***. ~~Slides will be labeled with a laboratory unique identification number (brady number) and stored in standard microscope slide boxes with a desiccant for at least 5 years.~~ Thick and thin blood films will be prepared and stained in duplicate by standard Giemsa technique and read by microscopists at either ~~Ilha Josina~~ ***Maragra*** Health Center (for samples obtained from infants and children meeting the case definition of clinical malaria) or at CISM (for the cross-sectional samples drawn at ~~0 , 5, 10, 12, 15, and 20~~ ~~***and***~~ ***24*** months of age). ***A drop of blood will also be collected on filter paper. Slides and filter papers will be labeled with a laboratory unique identification number (brady number).*** One slide will be read and the second slide will be kept solely as a back up, in case the first slide is lost or damaged. Asexual stage parasites and white blood cells in 200 high power fields (HPF) will be

counted on thick smear, and parasitemia will be reported as the number of parasites per 200 white blood cells. A total of 200 HPF will be examined before a slide can be declared negative for malaria parasites. All positive smears will be identified to species by examination of the thick and thin smear. Presence of gametocytes will also be recorded. Each slide will be read independently by two microscopists with expert qualifications. If discrepancies arise, a third microscopist will re-read the slide. This result will be considered final for the purposes of the final analysis. The first reading, which will be available within the following few hours after blood sampling, will be used for subject handling (treatment). ***Slides will be stored in standard microscope slide boxes with a desiccant for at least 5 years.***
(Amended 15 October, 2004) (Amended 2 December, 2004) (Amended 11 July, 2005)

c) Hematocrit (Hct): A portion of the finger/heelprick blood will be collected into a microcapillary ~~at cross-sectional sampling (0, 5, 10, 12, 15, 20 and 25 months) or~~ ***~~at cross sectional samplings at months 2,5 and 24 or~~*** for evaluation of a clinical episode for measurement of Hct, along with the smears. Infants found to have a Hct < 25% at any point during the study will be treated as per standard of care. **(Amended 2 December, 2004) (Amended 11 July, 2005) (Amended 10 August, 2005)**

~~d) Full blood count (FBC): A portion of the finger/heelprick blood will be collected into a microtainer at cross sectional samplings at months 0 and 24.~~
d) Full blood count (FBC): A portion of the finger/heelprick blood will be collected into a microtainer at all cross sectional samplings.
(Amended 2 December, 2004) (Amended 11 July, 2005) (Amended 10 August, 2005)

~~e) d)~~ e) Weight and length: Children will be weighed and measured at the health post during the cross-sectional samplings. **(Amended 2 December, 2004) (Amended 11 July, 2005) (Amended 10 August, 2005)**

2. Immunological data

a) Serology:
- Anti-PfEMP-1 antibodies
- Antibodies against asexual stage Pf antigens (MSP-1, AMA-1, EBA-175), ***anti-hemozoin*** and anti-GPI ***(ELISA and Functional Neutralization Assay)***

- Antibodies against asexual stages of *P. falciparum*: IgG, IgM, IgG1, IgG2, IgG3 and IgG4

b) Cellular:
- Pf Antigen-specific levels of IFN-γ and IL-10 cytokines in the blood
- Levels of IFN-γ, TNFα, IL-2, IL-10, IL-5, IL-12 and IL-4 cytokines in plasma

c) *Host genetics*: *Polymorphisms in the following genes*
- ***Hematological: HgbS, HgbAF and α-thalassemia***
- ***Specific candidate genes: ICAM-1, CD36, iNOS, CR1, MBP and TNFα***
- ***Th1/Th1 pathway genes: IL-1, IL-1R antagonist, IL-10, CD14, IL-4, IL-4Rα, IL-13, TLR2, TLR4, IL-12B IFN$\gamma$and IFNγR1***
- ***Other genes that may influence malaria infection***

d) Oxidative stress:
- ***Se, Cu, Zn and Fe***

- Glutathion, Glutathion peroxidase, Catalase and Superoxide dismutase (SOD)
- Thiols, AOPPs, HNE plasma protein adducts
- Lipoperoxides in RBC ghosts, hemichromes in ghosts, HNE-ghost protein adducts

(Amended 11 July, 2005) (Amended 10 August, 2005)

viii. Outcome Variables

1. Clinical outcome variables

a) Primary clinical outcome variable

The experience of having at least one episode of clinical malaria in the second year of life ***detected by passive case detection.*** **(Amended 2 December, 2004)**

b) Secondary clinical outcome variables

1. Fever (axillary temperature $\geq 37.5^{o}$ C or a history of fever in the previous 24 hours)
2. Hct during first, second and subsequent malaria infections, and during the ***24 months*** cross-sectional surveys.
3. Density of parasitemia during first, second and subsequent malaria infections, and during the ***24 months*** cross-sectional surveys.
4. Time to first clinical episode during the second year of life ***detected by passive case detection***.
5. Number of clinical episodes during the first year of life ***detected by active or passive case detection***.
6. Number of clinical episodes during the second year of life ***detected by passive case detection***.
(Amended 2 December, 2004)

2. Immunological outcome variables

a) Primary immunological outcome variables:

1. Anti-*Pf*EMP1 antibodies in peripheral blood, as measured at ~~0, 5, 10, 12, 15, 20 and 25~~ ***2.5, 5.5, 10.5, 15 and 24*** months of age. The outcome variable to be compared between the cohorts will be:
 -Antibody units converted from median fluorescence intensity [31]

2. ~~Level~~ ***Magnitude and frequency*** of cytokines (IFN-γ, IL-10) in peripheral blood in response to *in vitro* stimulation with Pf parasite antigens, as measured at ~~0, 5, 10, 12, 15, 20 and 25~~ ***2.5, 5.5, 10.5, 15 and 24*** months of age

3. Ratio of Th1 (IFN-γ, TNFα, IL-12 and IL-2) to Th2 (IL-10, IL-5, IL-4) cytokines in plasma, as measured at ~~0, 5, 10, 12, 15, 20 and 25~~ ***2.5, 5.5, 10.5, 15 and 24*** months of age.

4. Anti-GPI antibodies in peripheral blood as measured at 2.5, 5.5, 10.5, 15 and 24 months of age. The outcome variables to be compared between the cohorts will be:
 -Antibody units converted from ELISA titre
 -Antibody units converted from Functional Neutralization Assay.

5. Host genetics:

-Presence of homozygote or heterozygote for each particular SNP.

-Presence or absence of nominated haplotype for each gene.

(Amended 11 July, 2005) (Amended 10 August, 2005)

b) Secondary immunological outcome variables:

1. Total IgG and IgM IFAT titers against Pf RBC parasite antigens.

2. Ratio of cytophilic-to-non-cytophilic (IgG1+IgG3)/(IgG2+IgG4) IgG isotypes against Pf erythrocytic stage antigens, as measured by IFAT and ELISA.

3. Parasite strains at presentation (determine whether recurrent infections with same parasite strains (implies no/minimal induction of homologous immunity), or new infections with different strains (potential induction of immunity against strains to which the individual has been previously exposed).

4. Ratio of IgM, IgG1, IgG2, IgG3 + IgG4 isotypes against Pf GPI and hemozoin toxins as measured by ELISA.

5. Oxidative stress:

-Quantification of the antioxidant potential measured as enzymatic activity of antioxidant enzymes SOD, catalase and glutathione peroxidase.

-Quantification of the oxidative challenge of the host measured as level of glutathione in RBCs, hemichromes, lipoperoxides and HNE protein adducts in the RBC membrane, thiols, advanced oxidative protein products and HNE protein adducts in plasma.

(Amended 11 July, 2005) (Amended 10 August, 2005)

ix. Sample Size

Population Available: There are approximately 400 infants born yearly ~~in the catchment area of the Ilha Josina~~ ***at the maternity of the Maragra*** Health Center. Based on previous experience, we estimate that most mothers will be interested in participating in the study. We expect that ~~less than~~ ***around*** 20% of the pregnant women will be HIV-positive and that ~~25%~~ ***20%*** of the enrolled HIV-negative pregnant women will not enroll their infants due either to drop-out or to birth of an unhealthy or premature infant. We expect a drop-out rate of approximately ~~15%~~ ***20%*** during the two years each child participates in the study. Infants will be enrolled on a continuous basis as they are born, and enrollment will continue for one year (thus the study will be executed over a three year period). **(Amended 15 October, 2004)**

Sample size calculations: All sample size calculations are based on $\alpha = 0.05$ (two-tailed) and $\beta = 0.20$. With **n = ~~65~~ 78** per cohort, we have 80% power (using a two-tailed test) to detect a relative risk of 2 in Cohort 2 as compared to Cohort 1 for at least one clinical malaria episode in the second year of life and to detect of relative risk of 2 in Cohort 2 as compared to Cohort 3***, assuming an incidence of clinical malaria of 0.4 episodes per person-year at risk***. Because *a priori* we intend to make 3 comparisons (Cohort 1 to Cohort 2, Cohort 1 to Cohort 3, and Cohort 2 to Cohort 3), adjustment of the

p value using the Dunn-Sidak method is necessary. Thus, we consider significant a p-value lower than 0.017. We expect to see similar risks between C1 and C3. We will have 80% power to determine that the relative risk is not greater than 2 between the Cohorts 1 and 3. However, detecting a smaller difference would require much larger sample sizes (i.e. ~~2074~~ ***2413*** per cohort to detect a difference of 15%) and is not practical for the purposes of this study.
(Amended 15 October, 2004)

We anticipate a *~~15%~~* ***20%*** *loss to follow-up* and thus our **sample size** will be increased accordingly to **n = ~~77~~ *98* per cohort**. ***We will first enroll all infants aged 0-2 months from the Maragra area who are eligible for the study and whose mothers have signed an informed consent. We will then start recruiting pregnant women and enrolling their eligible infants as they are born. The number of pregnant women to be enrolled will depend on the number of infants 0-2 months of age recluted in the community.*** Because we expect that approximately ~~25%~~ ***20%*** of the pregnant women initially enrolled will not ultimately be included in the study (due to drop-out or delivery of a baby who is not full-term and healthy) we will ~~enroll 103~~ ***~~122 HIV-negative~~*** ~~mothers per cohort or a total of 309~~ ***~~366~~*** ***increase the number of HIV-negative*** pregnant women ***to be enrolled accordingly***. Given that our previous data indicates that there are at least 400 births per year in ~~Ilha Josina~~ ***Maragra***, we anticipate no problems in enrolling the required number of volunteers over a one year period. **(Amended 15 October, 2004) (Amended 2 December, 2004)**

The incidence of clinical malaria in study participants during the first year of the study was lower than expected. The sample size of the study was thus increased to n=350 total participants in order to increase the power of the study. **(Amended 12 September, 2006)**

x. Immunological Studies

1. Distribution of blood samples and prioritization

1-1.5 ml Whole blood

~~Heparin: 0.5ml cytokine intracellular staining/FACs~~

~~0.25 ml IFN-γ (Th1)~~

~~0.25 ml IL-10 (Th2)~~

EDTA: ~~0.5-1ml~~ ***1-1.5 mL*** to separate, cryopreserve ***and send to collaborators***

Plasmas:

Flow cytometry: anti-PfEMP1
ELISA: anti-MSP1, AMA-1, EBA-175, GPI
Cytometric bead array or equivalent
(IFN-γ, IL-10, TNF-α, IL-5, IL-4, IL-2, IL-12)
IFAT: IgG isotypes, IgM
Oxidative stress markers
Functional Neutralization Assay (GPI, hemozoin)
Archiving at CISM

~~Packed~~ red *and white* blood cells

Flow cytometry
Genotyping host ~~(only at birth)~~, Hgb AF
Genotyping parasite (if parasitemic)
Oxidative stress markers
Cytokines, Archiving at CISM

(Amended 11 July, 2005) (Amended 10 August, 2005)

Samples collected will be aliquoted at the CISM laboratory and frozen. Part of the samples will be assayed for immunological studies at the CISM and part will be sent in batches to the following collaborators to be processed at their laboratories:

-Centre de Salut Internacional, Hospital Clínic, Barcelona, Spain: frozen plasma samples will be sent to them, to be assayed for P. falciparum antibodies and extracellular cytokines.

-Blood Research Laboratories, University of Oxford, UK: frozen plasma samples will be sent to them, to be assayed for anti-haemozoin antibodies and biochemical oxidative stress markers

- Dept. Genetics, Biology and Biochemistry, Università di Torino: frozen plasma and pellet samples will be sent to them, to be assayed for oxidative stress markers

- Immunogenetics Research Group, Univ. Western Australia, Perth, Australia: frozen pellet (PBMCs and RBCs) will be sent to them to determine host genetic factors.

- The Walter and Eliza Hall Institute of Medical Research, Parkville, Australia: frozen plasma samples will be sent to them to be assayed for anti-GPI antibodies.

The remaining aliquots will be stored at CISM to be assayed for determinations contemplated in this protocol.
(Amended 10 August, 2005)

Based on previous experience, it is anticipated that a 1-1.5 ml volume of peripheral blood will be collected at each of the specified time points. The investigators on this proposal have considerable experience in collecting 1 ml volumes of blood via fingerprick from infants [51, 52, 53, 55] and no problems are anticipated in this regard. However, if for some unanticipated reason, problems are encountered, with permission of the mother of guardian, a 1 ml blood sample will be collected by venipuncture, by a qualified and experienced worker. However, in the event of lost or unavailable full volume, the prioritization of sample volume is as follows:

Whole blood:

1) separation of plasma and parasitized red blood cells (pRBC)
2) IFN-γ intracellular staining/FACs with crude whole parasite extracts, ***proteins and/or antigens***
3) IL-10 intracellular staining/FACs with crude whole parasite extracts, ***proteins and/or antigens***

(Amended 11 July, 2005)

Plasma:

1) Flow cytometry: anti-PfEMP1
2) ELISA: MSP1, AMA-1, EBA-175, GPI, hemozoin
~~2~~ ***3***) Cytometric bead array or equivalent (IFN-γ, IL-10, TNF-α, IL-5, IL-4, IL-2, IL-12)
~~3~~ ***4***) Blood stage IFAT: total IgG
5) FNA: GPI, hemozoin
~~4~~ ***6***) Blood stage IFAT: IgG isotypes
~~5~~ ***7***) Blood stage IFAT: total IgM
~~6~~ ***8***) Archiving at CISM

Parasitized red blood cells (pRBC):

1) Flow cytometry: anti-PfEMP1
2) Parasite genotyping
3) Archiving at CISM

Unparasitized red *and white* blood cells ~~(RBC)~~

1) Oxidative stress markers
~~1~~ ***2***) Host genotyping
~~2~~ ***3***) Archiving at CISM

(Amended 11 July, 2005)

2. Antibody Immunoassays

Detection of antibodies to variant antigens on the surface of PRBC by flow cytometry (FACS): This method has been shown to correlate well with the traditional agglutination assay [74,75]. Purified cultures of P. falciparum-infected erythrocytes will be stained with ethidium bromide and mixed with human serum previously adsorbed with uninfected erythrocytes, as described previously [31]. Antibodies to variant antigens on the surface of PRBC will be detected by FACS after incubation with a goat anti-human IgG and a FITC-conjugated rabbit anti-goat IgG. Results obtained as mean fluorescence index will be transformed to antibody units.

Asexual stages IFAT: Ring forms, trophozoites and schizonts of Pf will be prepared from infected human blood obtained from *in vitro* cultivation of (a) field isolate(s) ***from the Manhiça area or from laboratory-established strains*** ~~from Ilha Josina~~. Concentrated PRBC suspensions will be enumerated and diluted to deliver approximately 1000 pRBC per 100 μl dropped into a standard IFA slide depression chamber. Air-dried and acetone-fixed slides will be stored at –70°C until immediately prior to use. Plasma diluted 1:100, 1:500, 1:1000, 1:5000, and 1:10,000 will be tested among children in duplicate, along with positive and negative control plasma (from an adult with lifelong exposure to malaria, and a malaria-naïve American or European, respectively). A separate assay will be

conducted for each of the following on each study subject: IgG, IgG1, IgG2, IgG3, IgG4 and IgM utilizing the appropriate goat anti-human antibody.
(Amended 15 October, 2004) (Amended 11 July, 2005)

Asexual stages antigens by ELISA: Antibody responses to a panel of recombinant merozoite surface proteins will be measured [82]. Plasma diluted 1:500 will be tested among subjects in triplicate, along with positive and negative controls. A separate assay will be conducted for each antibody isotype: IgG, IgG1, IgG2, IgG3, IgG4 or IgM utilizing the appropriate horseradish peroxidase-conjugated polyclonal sheep anti-human antibody. Specific reactivity of a serum will be calculated by subtracting optical density (OD) values for the buffer controls from the value obtained for the recombinant protein to obtain specific OD values. These values will then be used as a continuous variable for statistical analysis, or converted to a binary variable (positive vs. negative) using a cut-off defined for each antigen as the mean + 2 standard deviations of 50 negative control serum samples. We will also attempt to measure antibodies in these samples using 'Luminex' fluorescent bead-based high-throughput technology.

Malaria toxins by ELISA and Functional Neutralization Assay: We will measure antibody responses to hemozoin and to compositionally pure native GPI, chemically synthetic GPI and synthetic epitopes. **(Amended 11 July, 2005)**

~~***Assessment of antioxidant potential:***~~

~~***- Plasma: Quantification of Se, Cu, Zn and Fe, known co-factors of relevant anti-oxidant enzymes (done in Oxford).***~~
~~***- Erythrocytes: Glutathion, Glutathion peroxidase, Catalase and Superoxide dismutase as measure of anti-oxidant host response will be quantified in fresh lysed erythrocytes by spectrophotometry (done at CISM and in Italy).***~~

~~***Assessment of oxidative damage (done in Italy):***~~

~~***- Plasma: Thiols, AOPPs, HNE plasma protein adducts will be measured by spectrophotometry***~~
~~***- Erythrocytes (ghosts): lipoperoxides in RBC ghosts , hemichromes in ghosts, HNE-ghost protein adducts will be quantified by luminol enhanced chemiluminescence and flow cytometry***~~

(Amended 11 July, 2005) (Amended 10 August, 2005)

3. Cellular Immunoassays

Since it is likely that cellular protection is mediated by complex interactions between cytokines and chemokines, we are planning to look at a representative panel of Th1 and Th2 cytokines. We will focus on IFN-γ, IL-10, TNF-α, IL-5, IL-4, IL-12, but depending on the availability of blood we also intend to assess IL-12, IL-6, TGF-β and MIG. We will use two complementary approaches: (i) detection of total cytokine levels secreted in the plasma, and (ii) detection of malaria antigen-specific cytokines contained within blood lymphocytes.

Multiplexed bead assays by flow cytometry (FACS) or equivalent: Human Th1/Th2 Cytokine Kit (Cytometric bead array, BD Biosciences) or equivalent. This microparticle-based flow cytometry assay allows simultaneous measurement of six cytokines (IFN-γ, IL-10, TNF-α, IL-5, IL-4 and IL-2) secreted in a single sample of plasma. There is potential that further cytokines may be available in the near

future. It requires very small quantities of sample (5-50 µl), making it very useful for studies in infants. This method generates data comparable to ELISA based assays but in a "multiplexed" fashion, and with more sensitivity (9-15 pg/ml for these cytokines) and reproducibility [76]. Concentration of unknown cytokines is calculated for the CBA as with any sandwich format assay, i.e. through the use of known standards and plotting unknowns against a standard curve, and results can be easily analyzed by linear regression. Forward vs. side scatter gating is employed to exclude any sample particles other than the polystyrene beads (7.5 µm). Data is displayed as two-color dot plots (FL-2 vs. FL-3) such that the six discrete FL-3 microparticle die intensities are distributed along the Y-axis. A wide variety of preset configurations enable the user to set up standard dilution series, generate calibration curves with either 4-parameter logistic or log-log curve fit models, and subsequently compare unknowns. The data output from a CBA is not restricted to numbers from a curve: the BDA dot plot display generated via a flow cytometer creates unique profiles or signatures for each cytokine that provide with additional information at a glance.

P. falciparum **antigen:** We propose to use crude whole parasite extracts prepared from ~~Ilha Josina~~ isolates ***from the Manhiça area*** collected during previous studies ***or from laboratory-established strains***, in order to cover the entire spectrum of parasite antigens expressed during the asexual stages of the cycle. If, for some unanticipated reason, we experience difficulties with the whole parasite extract (for example, high nonspecific background reactivity) we will use selected pools ***of recombinant proteins and*** MHC class I and class II peptides representing a group of well characterized pre-erythrocytic and erythrocytic Pf antigens ***such as***~~:~~ CSP, LSA-1, SSP-2, MSP-1 and MSP-3. The parasite extract will be tested for endotoxin levels [77]. Currently there is a lot of effort by the groups in Oxford, Kenya and elsewhere (C. Newbold, K. Marsh, J. Langhorne, personal communication) to define what are the important immunogenic regions of PfEMP-1. If such data becomes available during the execution of the proposed studies, we will also include in our evaluations synthetic peptides that represent those regions in order to more fully characterize anti-PfEMP-1 specific immune responses. **(Amended 15 October, 2004) (Amended 11 July, 2005)**

~~**Intracellular cytokine staining and FACS analysis (BD FastImmune Cytokine System):** We propose to use the commercially available FastImmune™ CD4 Intracellular Cytokine Detection Kits (BD Biosciences) to detect antigen-specific CD4+ and CD8+ T cell cytokine responses in whole blood; this FastImmune Cytokine System has been optimized to detect human cytokines in small sample volumes with speed (results in hours not days), and high throughput. More specifically, using the protocol recommended by the manufacturer, we will measure the cytokine expression in small volumes (0.25 ml per assay) of whole blood collected in sodium heparin, and processed in polypropylene tubes with minimal sample manipulation and maintaining the *in vivo* environment. We and others have shown that fresh (<24 hours) whole blood yields the same results as fresh PBMCs in RPMI, and that there is a good reproducibility in assays with fresh *vs*. frozen samples: Results from intracellular staining assays carried out according to the recommended procedure are expressed as frequency (% cytokine positive CD4+ or CD8+ T cells) and intensity (mean fluorescence intensity). This assay has similar sensitivity to real time PCR for detection of cytokine mRNA, but may not be as sensitive as the ELIspot assay; the requirement for substantial amounts of PBMCs precludes the use of the ELIspot assay in our target population of infants. In addition to quantitation of cytokine levels, this FastImmune method allows concomitant detection of multiple parameters including phenotype, activation status, cell proliferation and cell cycle status at a single cell from a single sample, depending on the cell markers stained. This information will allow us to define the maturity of the T cells and further characterize the immune status of the infant.~~

Cytokines: we will measure the cytokine expression in the supernatant collected from fresh PBMCs that have been previously stimulated. The PBMCs will then be used to quantify mRNA to determine profiles of cytokine gene expression.
(Amended 10 August, 2005)

4. Molecular biology procedures

Parasite genotyping and multiplicity of infection using MSP-1 and MSP-2 polymorphic markers: The polymorphism of *MSP-1* and *MSP-2* genes are routinely used as markers of parasite genetic diversity in the field [79] and type-specific oligonucleotide primers which identify distinct allelic forms of *MSP-1* and of *MSP-2* and the presence of mixed infections have been designed. An aliquot of 20-100 μl cryopreserved RBCs ***or blood from filter paper*** will be used for extraction of *P. falciparum* DNA by the quick boiling method [80] ***or equivalent***. Then, a hot-start nested PCR reaction which amplifies the polymorphic block 2 of *MSP-1* will be carried out. Primers O1 and O2 will be used in the outer reaction, as described previously [81] ***or equivalent***. The nested amplification will be carried out using 1-2 μl of the previous reaction and 3 sets of block 2 type-specific primers to distinguish the three known types of block 2 of *MSP-1*, K1, MAD20 and RO33, and to detect size differences among alleles of each type, as described [82]. Dimorphic regions of *MSP-2* will be typed by the dimorphic-form specific (DIFS) PCR method [83] ***or by equivalent methods***. Briefly, a combination of 3 primers consisting of two 5' type-specific primers and a 3' common primer will distinguish between IC1 and FC27 alleles of *MSP-2* (primers difs A, B and C) by differences in the size of bands in a hot-start single PCR.
(Amended 11 July, 2005)

Host genotyping and Haemoglobin AF: Because of the potential for confounding, we propose to ~~do host genotyping~~ ***examine a series of genotypes that have been selected as being of most potential importance to the risk of malaria infection in early life. The genes of interest can be considered in three general groups:***

1. Known hematological genetic factors (including sickle cell (HgbS) [84] and Hgb AF detected by haemoglobin electrophoresis and α-thalassemia [85]) detected by a PCR based approach.
2. Variations in candidate genes known to be important in inflammatory or immunological responses to infection with malaria (**ICAM-1and CD36, MBP, iNOS, TNFα)**
3. Genes or related genes involved in Th1/Th2 immune responses (IL1, CD14, IL-4, IL-4Rα, IL-13, TLR2, TLR4, IL-1β, IL-6, IL-12B, IFNγreceptor 1, IFNγ)

~~for sickle cell (HgbS) [84], α-thalassemia [85] and other polymorphisms of genes relevant to the immune response. The presence or absence of Haemoglobin AF will also be determined. Based on previous data (see Section C) we do not anticipate, however, that subjects enrolled in this study will have genetic markers which could adversely affect the outcome of the proposed study. Determination of HgbS and HgbAF will be done by hemoglobin electrophoresis and α-thalassemia screening will be done by PCR-RFLP. If new laboratory techniques become available before the start of the study to detect the mentioned polymorphisms, these methods will be incorporated and used.~~

Due to recent advances in DNA amplification methodologies, blood specimens as small as one ml are now sufficient for multiple, accurate DNA analyses [112].
(Amended 11 July, 2005)

5. Biochemical assays

Assessment of antioxidant potential:

- Plasma: Quantification of Se, Cu, Zn and Fe, known co-factors of relevant anti-oxidant enzymes.

- Erythrocytes: Glutathion, Glutathion peroxidase, Catalase and Superoxide dismutase as measure of anti-oxidant host response will be quantified in fresh lysed erythrocytes by spectrophotometry.

Assessment of oxidative damage:

- Plasma: Thiols, AOPPs, HNE plasma protein adducts will be measured by spectrophotometry

- Erythrocytes (ghosts): lipoperoxides in RBC ghosts , hemichromes in ghosts, HNE-ghost protein adducts will be quantified by luminol enhanced chemiluminescence and flow cytometry

(Amended 10 August, 2005)

xi. Data Analysis and Results Interpretation

1. Clinical data

a) Overall Analytical Plan

The primary aim of the study is to determine the effect of timing of exposure to Pf erythrocytic stage antigens during infancy (0 to 5 vs 5 to 10 months of age) on subsequent morbidity due to clinical malaria. The primary outcome variable was chosen as the presence or absence of at least one episode of clinical malaria during the second year of life ***as detected by passive case detection***, because current epidemiological data suggests that this outcome is a sensitive, inverse indicator of NAI; when used as the primary outcome variable in studies detailed earlier in this proposal (see background and rationale sections) significant differences were detected between cohorts receiving differing prophylaxis [1, 2]. In the study site, an incidence of ***0.4*** ~~0.48~~ episodes of clinical malaria per person year at risk (Aponte, personal communication). Thus, as described in the sample size section, each of the three cohorts will be compared to the others in pairwise fashion, with the p value for significance reduced to 0.017 to adjust for the three comparisons. **(Amended 15 October, 2004) (Amended 2 December, 2004)**

However, other variables could also affect the likelihood of experiencing at least one clinical episode during the second year of life, such as number of clinical episodes experienced during the first year of life, regardless of the age at which they occurred. In addition, we believe that immunological responses during the first year of life may potentially explain the differences in susceptibility to clinical episodes during the second year (secondary aim), although other factors, such as host genotype (e.g., presence or absence of sickle cell trait, α-thalassemia or others), the presence of haemoglobin AF, parasite genotype, and possibly maternal immune responses, could also affect the primary outcome variable. Because we do not know which of these many factors will be most closely associated with the outcome, we propose a logistic regression analysis to make this assessment. All of these factors will be used in a multiple logistic regression model, and those with significant associations will be identified. Although it is by no means assured, we hope that one of the immunological measurements (see below) will show a significant association and thus will serve as a positive or negative correlate with NAI. A rationale has been provided regarding the concentration of antibody responses to PfEMP-1 as a likely candidate.

A similar logistic analysis will be conducted with a second important clinical outcome: whether or not each participant experiences a Hct < 25% during the second year of life, as measured during the ~~12, 15, 20 and 25~~ ***24*** month cross-sectional survey~~s~~. Although it is perhaps less clear how the immunological responses we are measuring might impact on Hct, it is nevertheless a primary indicator of morbidity and also a primary target for interventions such as anti-morbidity malaria vaccines or prophylaxis regimens, and is proposed here as a secondary outcome. **(Amended 2 December, 2004)**

b) Overall Interpretation of Results

Our prediction: We hypothesize that the relative risk of having at least one clinical episode of malaria in the second year of life will be 2.0 or greater in Cohort 2 as compared to Cohort 1 and 3. If this is true, it will support our hypothesis that the infant immune system is able to mount protective responses provided that exposure occurs following month 5, but cannot do so if exposure occurs earlier but is absent between months 5 to 10. This finding would have important implications for future vaccine trials, for which the timing of administration of malaria vaccines would then be judged to be critical if they are to induce protection against blood stage infection.

Another expected finding of this study is that Cohort 1, and to a lesser extent Cohort 2, will have reduced frequencies of parasitemia and clinical episodes of malaria during the first year of life, due to the protective effects of SP + AS administration. A key strategy for public health planners is how to achieve this protective effect without causing a rebound parasitemia during the second year of life. Ultimately, it may be important to repeat studies such as this in several geographic locations, in order to assess how a variety of transmission intensities, parasite genotype frequencies, host populations, vector populations, etc., may influence the relationship between the administration of prophylaxis, in particular its intensity and timing, and lasting gains in child health.

c) Additional Analyses of Clinical and Parasitological Data: Cross sectional and Prospective.

The data recorded during weekly visits by research team members and during trips to the health center initiated by the participants' mothers/guardians will provide a relatively detailed accounting of the number of clinical episodes of malaria throughout the study. Data collected regarding episodes will include vital signs (in particular, axillary temperature ***and respiratory rate***), Hct, and parasite genotype. ~~In addition, cross-sectional prevalence surveys will be conducted at several time points, allowing a relative assessment of the prevalence and intensity of parasitemia among the three cohorts.~~ Cross sectional surveys will also provide data ~~on anemia,~~ on the developing immune response and on the presence or absence of Hgb AF (see below) ***and the 24 months cross-sectional survey will also provide data on anaemia and parasitaemia***. **(Amended 2 December, 2004)**

We will look for differences among cohorts in all of these measures at defined cross-sectional timepoints, and intervening intervals where appropriate, during the course of the first two years of life. ~~For example, we will compare the time to first parasitemia in the three cohorts, following the 10 and 12 month cross-sectional surveys.~~ We will also assess the relationship between these measures and the primary outcome variables (clinical episodes and severe anemia), and thus their ability to predict clinical outcomes during the second year of life. **(Amended 2 December, 2004)**

2. Immunological data

a) Analytical plan: cross-sectional and prospective

In order to assess the development of protective immunity in infants, we have chosen to measure two different immunological parameters as markers of acquired immunity to malaria: an antibody response

(flow cytometry) and a cellular response (cytokines). Specifically, we will measure the type and quality of immune responses at ~~7~~ ***5*** cross-sectional time points~~, and at the time of clinical episode (acute and convalescent)~~, and will compare these immunological outcomes with the clinical outcome(s) for each cohort and between cohorts. **(Amended 2 December, 2004)**

***Our prediction*:** We hypothesize that the level of antibodies will be statistically significantly lower in Cohort 2 as compared to either Cohort 1 or Cohort 3 at the 10 month old cross-samplings and that this reduction will correlate with an increased relative risk of one or more clinical episodes of malaria in the second year of life.

Proposed analysis*:* Immunological endpoints will be based upon levels of antibodies (intensity) and the ratio of Th1/Th2 cytokines at each of the cross-sectional time points. For the first analysis, values for antibodies and cytokine ratios for each cohort will be compared at each cross-sectional timepoint. For the second analysis, values for antibodies and cytokine ratios at each of the timepoints will be correlated with risk of clinical outcome(s) for each cohort and between cohorts.

Immunological data will be analyzed in two different ways:

- Cross-sectionally the key analysis will be comparison of immunological markers between cohorts, not individuals, to show differences in the development of acquired immunity to malaria attributable to age of first exposure to Pf antigens. The key timepoint for comparison is considered to be 10 months. We will explore the profile of immune responses and how markers change with age. Specifically, we predict the following outcomes:

X1; t= ***2,5 month*** ~~0~~ ~~***or enrollment***~~: No difference between the 3 cohorts

X2, t=~~5~~ ***5,5*** month: No difference between the 3 cohorts

X3, t=~~10~~ *10,5* month (*key comparison*): Cohort 2 will have significantly lower levels of anti-PfEMP-1 antibodies and/or Th1/Th2 cytokine ratios than cohort 1 and 3. Cohort 1 = cohort 3.

~~**X4**, t=12 month: same as X3.~~

~~**X5**~~ ***X4***, t=15 month: same as X3.

~~**X6**, t= 20 month: No difference between the 3 cohorts.~~

~~**X7**~~ ***X5***, t= ~~25~~ ***24*** month: same as X6. We expect the immune responses to equalize between cohorts by 20-25 months.

(Amended 2 December, 2004) (Amended 11 July, 2005)

- Prospectively: To look for correlates of immune responses and clinical outcome. Specifically, acquisition of immune response at the level of the individual subject (as opposed to cohort) in cohort 1 vs. 2, and correlation with how that individual responds to infection and the relative risk of clinical disease. We will evaluate whether lack of exposure at different ages (0-5 months, or 5-10 months) during first 2 years of life results in differences of antibody and/or cytokine markers between the different cohorts.

Acute samples: we will define the genotype of the infecting parasite(s) to determine whether infants are susceptible to parasite strains to which they have previously been exposed (presence or absence of strain-specific immunity). ***Parasites will be cryopreserved to be used for antibody recognition.*** **(Amended 10 August, 2005)**

~~**Convalescent sample**: we will investigate the effect of exposure to Pf erythrocytic antigens on subsequent antibody and cytokine responses evaluated as described above. This will be related to immune responses detected at the time of cross-sectional sampling either side of the clinical episode. The cross-sectional comparison is considered to be the primary immunological outcome but data obtained with the acute and convalescent samples will enhance our capacity to test our hypothesis.~~
(Amended 2 December, 2004)

Convalescent sample: we will investigate the effect of exposure to Pf erythrocytic antigens on subsequent antibody and cytokine responses evaluated as described above. This will be related to immune responses detected at the time of cross-sectional sampling either side of the clinical episode.
The cross-sectional comparison is considered to be the primary immunological outcome but data obtained with the acute and convalescent samples after the first episode of malaria will enhance our capacity to test our hypothesis.

(Amended 11 July, 2005)

b) Analysis and interpretation of antibody immunoassays

Primary outcome: **Anti-*Pf*EMP1 antibody** by flow cytometry in peripheral blood:
The variable outcome measured is:
-Antibody units converted from median fluorescence intensity [31]

Secondary outcomes:
IFA titers against *P. falciparum* infected erythrocytes
Titer $\log_2$(titer/10)+1 with reference to last dilution causing positive fluorescence as compared with non-specific background staining. In addition, we will determine the cytophilic-to-non-cytophilic (IgG1+IgG3)/(IgG2+IgG4) ratio in those plasma samples with detectable IgG antibody levels.

Anti-GPI and anti-hemozoin antibodies by ELISA and FNA in peripheral blood:
The outcome variables measured are:
-Antibody units converted from ELISA titre (titer $\log_2$(titer/10)+1 with reference to last dilution causing positive ELISA as compared with non-specific background and the ratio of IgM, IgG1, IgG2, IgG3 + IgG4 isotypes as measured in the same way)
-Antibody units converted from Functional Neutralization Assay.
(Amended 11 July, 2005)

c) Analysis and interpretation of cellular immunoassays

- The **cytokine intracellular staining** of whole blood will provide information about Pf antigen specific expression of IFN-γ and IL-10 in peripheral blood, the phenotype of the lymphocyte population expressing each cytokine (produced by CD4 or CD8 T cells), as well as the status of activation (CD69 marker) of the cells. Data will be acquired by flow cytometry and analyzed using the CellQuest™ software. Results will be expressed as frequency (%) of cytokine positive CD4+ or CD8+ T cells, and mean fluorescence intensity. For statistical analysis, data on the mean frequency of cytokine expression will be compared between cohorts by t-test or regression analysis; $p<0.05$ will be considered statistically significant.
- The concentration of unknown cytokines (IFN-γ, IL-10, TNF-α, IL-5, IL-4 and IL-2 and IL-12) in plasma is calculated in the Cytometric Bead Array (CBA) or equivalent via extrapolation from a curve of known standards. Standard curves are plotted [cytokine calibrator concentration vs. FL-2 mean fluorescence intensity] using a 4-parameter logistic curve fitting model. Cytokine concentrations (pg/ml) are determined from these standard curves. If the plasma has a cytokine concentration below the detection limit for the assay, a value of 0 will be assigned. All data will be presented as mean ± standard error. In addition, cytokine ratios will be calculated to determine the Th1 vs. Th2 balance of cytokines.

Results will be analyzed using the manufacturer's software. Data will be analyzed by a general linear model analysis of variance (ANOVA) with pre-planned comparisons to generate two-tailored p-values. Fisher least significant-difference tests will be used to make appropriate post ANOVA comparisons.

d) Analysis and interpretation of immunogenetic data

Genetic data will be analyzed with respect to both intermediate and clinical outcomes. For each genotype examined, plasma specific antibody levels will be compared between homozygotes and heterzygotes within each of the three cohorts (to detect genetic influences within each study group) and between each of the cohorts (to detect genetic influences on differences in outcome caused by varying timing of anti-malarial treatment). In addition, to provide data to contribute to understanding the mechanism of action of NAI, for the genes for IFN-γ, IL-10, TNF-α, IL-4, and IL-12, plasma levels of each of these genes products will be compared between homozygotes and heterzygotes within each cohort and between each of the three cohorts (to detect genetic influences on differences in gene output (and hence serum levels) caused by varying timing of anti-malarial treatment). Similar analyses will be undertaken for other intermediate variables including hematological variables and intracellular staining assays. Genotype will also be analyzed with respect to clinical outcomes at each of the time points of data collection. In these analyses, appropriate correction will be made for multiple analyses.
(Amended 11 July, 2005)

e) Analysis and interpretation of oxidative stress data
Parameters for anti-oxidant potential of the host and for oxidative stress will be compared between cohorts in the cross-sectionals to analyse differences in the development of immune responses to malaria in correlation to risk of clinical malaria attributable to age of first exposure to P. falciparum infection
(Amended 11 July, 2005)

xii. Potential Difficulties

~~**1.** The location of the study site (40 km from Manhiça) poses logistical difficulties, namely the efficient transport of children (i.e., those requiring admission to the Manhiça Hospital), study personnel, equipment, and samples between Ilha Josina and Manhiça. A large phase IIb trial with the RTS,S candidate malaria vaccine has been recently conducted in Ilha Josina by CISM. Therefore the team already has experience in dealing with all these logistical problems and there are trained field and health workers based there. We believe that the benefits of working with this population in Ilha Josina outweigh the logistic difficulties. We will be increasing the capabilities of the Ilha Josina Health Center and will also have continuously accessible personnel on call in Ilha Josina.~~ **(Amended 15 October, 2004)**

2. Reliance of caretakers for passive case detection. Prior to study start we will ask all parents/guardians of study participants to take their child to the health center whenever he/she feels unwell. During the previous ***trials*** ~~vaccine trial~~ conducted ***by CISM*** ~~in Ilha Josina~~ the mothers/guardians responded well and attended the health ***facilities*** ~~facility~~ frequently. **(Amended 15 October, 2004)**

3. The volumes of blood which can be collected from newborn infants will limit the number and breadth of immunological assays which can be performed, but we have assigned priorities which we believe will allow us to meet our objectives. We anticipate that difficulties may be minimized by employing well-trained personnel experienced in working with newborns and infants.

4. We may not be able to adequately control for the effect of *in utero* exposure on T cell responses. We are collecting a medical history on the mother concerning previous births, and malaria infections. We are also collecting maternal blood, placental samples and cord blood in an attempt to gauge the extent of maternal infection at delivery. ***Nevertheless these maternal factors should be equally distributed between the three cohorts, given that infants will be randomly allocated to these.*** **(Amended 2 December, 2004)**

5. We do not know the specifics of decay of maternal antibodies in the population at ~~Ilha Josina~~ ***Manhiça*** and this could potentially confound our immunologic results. However, we are using data from Tanzania on the kinetics of maternal antibody decay in infants [49, 86] as a guide. Furthermore, the data obtained by CISM investigators indicates that there is a very low or non-existent incidence of clinical malaria episodes in infants less than 2 months in this area (see details included under Study Site), presumably due at least in part to the protective role of maternal antibodies, while the age-incidence of malaria episodes rises rather dramatically after 2 months of age; these data indicate that the presence of maternal antibodies after 2 months of age is not likely to be a major confounder in our proposed studies. **(Amended 15 October, 2004)**

6. Parasite diversity in the region of ~~Ilha Josina~~ ***Manhiça***. We will do parasite genotyping with each clinical episode of malaria. We also have data from previous studies on the most prevalent *P. falciparum* strains circulating in the community. **(Amended 15 October, 2004)**

7. Transmission rate. We have assumed this to be stable, although there may well be some seasonal variations. We will be using block randomisation with open enrolment over a one year period to address this possibility.

8. ~~Genetic factors and other factors of susceptibility and resistance. We will assess for the presence of HgbS, Hgb AF and α-thalassemia in the population, factors that we think could have a profound impact upon host morbidity and mortality. However, we have limited our evaluation of host genetic factors because of our small sample size and blood volumes.~~ ***Host genetic factors: We will assess both intermediate and disease phenotypes for associations with genotypes in specific genes that we have selected for study. The relatively small population being studied will reduce the chance of finding significant associations for alleles with a low population frequency.*** **(Amended 11 July, 2005)**

9. ~~Many~~ ***It is possible that some*** deliveries ~~will~~ take place at home and not in the maternity. In these cases it will not be possible to take samples or blood from the placenta or cord blood, thus these data will be missing. Peripheral blood from the mother ~~and child~~ will be obtained a few days after delivery. **(Amended 15 October, 2004) (Amended 10 August, 2005)**

10. For those infants aged 0-2 months enrolled after birth the data from the parasitological and immunological status at birth will be missing. Peripheral blood from the child will be obtained ~~when they are enrolled~~ at the 2,5 months cross-sectional sampling. **(Amended 2 December, 2004) (Amended 11 July, 2005)**

E. HUMAN SUBJECTS RESEARCH

Protection of Human Subjects

1. Risks to the Subjects

a) Human Subjects Involvement and Characteristics

Volunteer Selection:
a) Infants aged 0-2 months, identified in the community

The mothers of infants aged 0-2 months living in Maragra or Manhiça will be invited to participate in the study. Only women who visit the VCT center first and are HIV-negative will be eligible for the study. At the VCT center an informed consent will be obtained prior to doing the HIV-test or disclosing the results from a previous visit to the VCT center. If the woman is HIV-negative she will be informed about the risks and benefits of enrolling their infant in the study. If the woman is interested in participating a second informed consent will be obtained to enrol her infant in the study. After the informed consent has been obtained, one of the investigators will obtain a history and physical exam of the child and, if all inclusion criteria are met and there are no exclusion criteria, the child will be enrolled and randomized to either Cohort 1, 2 and 3.
(Amended 2 December, 2004) (Amended 10 August, 2005)

b) Pregnant women who attend the VCT center at the antenatal clinic in Maragra or Manhiça
Potential participants will be identified when they attend the VCT center at the antenatal clinic in Maragra or Manhiça. After the woman has undergone all standard procedures at the VCT center and the HIV result is known, the counselor will only invite HIV-negative women to participate in the study.
Healthy women, HIV-negative, residing in ~~the village of Ilha Josina~~ ***Maragra or Manhiça***, who are interested in participating in the study, will be informed about the risks and benefits of enrolling themselves and their infant in the study. ~~Only women who have visited the VCT center first and have been tested for HIV infection will be considered for participation. If the woman is HIV-negative, after~~ ***If the woman is interested in participating she will be asked to sign an informed consent. After*** completion of the informed consent process, and only after dated informed consent has been obtained, one of the investigators will obtain a history and physical exam of the pregnant female and if all inclusion criteria are met and the woman has no exclusion criteria, she will be enrolled. Upon delivery of a single healthy full-term infant, the mother will be questioned again about her wish to participate and if she still agrees to participate, the infant will be examined and enrolled. Upon enrollment, the infant will be randomized to either Cohort 1, 2, or 3.
The investigators will not have access to any of the VCT center records and will receive no information at all on the HIV-status of women who are not enrolled in the study.
(Amended 15 October, 2004) (Amended 2 December, 2004) (Amended 10 August, 2005)

- Inclusion criteria:

For pregnant females:

- Healthy females less than 50 years of age, who are obviously pregnant (no pelvic exams to be performed).

- Females who test negative for HIV ***and attend the VCT center at the Maragra or Manhiça antenatal clinic during the last*** ~~three~~ ***months of pregnancy***.
- Subjects willing to provide a single blood draw and placental tissue to the investigators at the time of delivery.
- Subjects willing to enroll their healthy full-term infants after delivery to participate in the cohort study.
- Those able and willing to give informed consent for themselves and their infant, are permanent residents of ~~Ilha Josina~~ ***Maragra or Manhiça***, and expect to be living in the area with their infant for at least 2 years.

For newborn infants:

- Full-term healthy infant, weighting greater than 1.5 kg.
- Mother was able and willing to give informed consent for participation of the infant in the study.

For infants aged 0-2 months recluted in the community:

- ***Mother is alive***
- ***Age <2 months old***
- ***Mother is HIV-negative and has attended the VCT center at the Maragra or Manhiça antenatal clinic.***
- ***Mother was able and willing to give informed consent for participation of the infant in the study, is permanent resident of Maragra or Manhiça, and expects to be living in the area with her infant for at least 2 years.***
- ***Have correctly received the EPI vaccines administered at birth.***

(Amended 15 October, 2004) (Amended 2 December, 2004) (Amended 10 August, 2005)

- Exclusion criteria:

For pregnant women ***or mothers of infants aged 0-2 months****:*

- Plan to leave ~~Ilha Josina~~ ***Maragra or Manhiça*** in less than 2 years from the start of the study.
-Women not willing to get tested for HIV infection at the VCT center.
-Test positive for HIV.
- Not willing to provide informed consent.
- Cannot understand either Portuguese or Changana (consent forms are written in these languages).

For newborn infants:

- Any obvious congenital malformation.
- Any signs of cerebral asphyxia.
- Any obvious neonatal infection.
- Prematurity
-Low birth weight (≤1.5 kg)
-Twins

For infants aged 0-2 months:

-Any obvious major chronic or acute disease or malformation
-Mother is dead
-Twins

(Amended 15 October, 2004) (Amended 2 December, 2004) (Amended 10 August, 2005)

b) Source Material
Source materials will include standardized questionnaires for collection of data at all study timepoints. Data collected during the visits to the ***Maragra or Manhiça*** health center will be collected through the standardized outpatients questionnaire used in Ilha Josina ***Maragra*** and Manhiça as part of the hospital surveillance system. Samples include a single blood sample from the mother after delivery that will be used to determine level of parasitemia and level of circulating antibodies. A placental sample (and a blood sample from the placental tissue) obtained post-delivery will be used to determine maternal level of infection at birth. Repeated 1-1.5 ml blood samples will be drawn from infants in order to determine genetic (at birth only), parasitologic, and immunologic indices. ***In infants who are recluted after birth, aged 0-2 months, no samples will be available from birth, thus and blood samples will be drawn at enrolment 2.5 months of age to determine genetic, parasitologic and immunological indices and during the cross-sectional surveys and active and passive case detection.***
(Amended 15 October, 2004) (Amended 2 December, 2004) (Amended 11 July, 2005) (Amended 10 August, 2005)

c) Potential Risks

The potential risks to the infant include (i) risks associated with prophylaxis, (ii) risks associated with malaria, (iii) risks associated with repeated blood draws.

(i) Risks associated with prophylaxis: The infants will receive prophylaxis either from birth to 5 months, from 5 months to 10 months, or not at all. The primary risk associated with S-P is that of mild to severe allergic skin rash in about 1 in 20,000 individuals. There is also a rare risk of granulocytopenia and thrombocytopenia. Artemisinin derivatives have so far been shown to be safe and well tolerated with a very low risk of severe adverse reactions [87, 88]. There is also a potential risk of increased risk of malaria episodes in the subsequent months after prophylaxis is stopped. However, it is not clear whether the overall risk of uncomplicated malaria at age 4 is higher and there is evidence that the cumulative rate of severe malaria and particularly of severe anaemia at age 4 is equal or lower among those who received chemoprophylaxis during the first year than among those who did not receive it (See section A above). SP was given to 701 infants in Ifakara, Tanzania [2] at 2, 3 and 9 months and there were no reported adverse events that were thought due to the drug or which prompted discontinuance of the drug. No episodes of skin rash secondary to SP were observed. SP+AS was given to 60 children and AS+Amodiaquine to 61 children in Manhiça, during a randomized trial of drug efficacy, and no severe adverse reactions attributable to the treatment were reported [89]. ***Furthermore, SP+AS prophylaxis and the EPI vaccines will be administered with a 15 days interval, a sufficient time lag to avoid any possible interactions.*** **(Amended 2 December, 2004)**

(ii) Risks associated with malaria: Malaria is an important cause of mortality in this area and there is a risk in this study that some children may die of the infection. The study site is heavily endemic, and infection is virtually certain. However, the infants and toddlers would face these risks whether the research team was present or not. The treatment of uncomplicated or complicated malaria in subjects of this research protocol applies only to drugs licensed for this use in Mozambique. The first-line treatment for malaria in this study for uncomplicated malaria will be oral SP plus Amodiaquine, the first line treatment in the country. Second and third line drugs for treatment failure will be Co-Artem or Quinine. In the case of severe malaria, the child will be transported to the Manhica Hospital and treated with intravenous quinine with medical support as per routine Thus, the risk and discomfort

endured by subjects is no more or less than that to be expected during the routine management of their infections. **(Amended 15 October, 2004)**

3) Risks associated with repeated blood draws: The subjects of this work will experience discomfort associated with the sticking of a finger, toe, or heel with a standard lancet. The procedure is painful, but only for an instant. While there is risk of infection at the site of puncture, that risk is slight, and any such infections will be monitored for and treated.

2. Adequacy of Protection Against Risks

a) Recruitment and Informed Consent:
The protocol, including the consent forms, will have to be approved by the IRBs of the Hospital Clínic in Barcelona (Comitè Ètic d'Investigació Clínica, CEIC) and the National Mozambican Ethical Committee. Implementation of the principles of the current edition of the Declaration of Helsinki have also been carefully followed.

Mothers of infants aged 0-2 months identified in the community will be asked to sign two informed consents. Because the investigators will not know the HIV status of these women, they will be referred to the VCT center at the antenatal clinic, the only place where confidentiality on the HIV results can be ensured. Once there they will be informed that being HIV-negative is a requirement to participate in the study and will be told about the benefits of being tested for HIV and the benefits of the antiretroviral treatment if the test is positive. They will receive the standard counseling at the VCT center and, if they agree to get tested, will be asked to sign an informed consent. This informed consent will not contain the name of the mother, but only the VCT center registration number and will be thumbprinted by the mother. After obtaining the informed consent, only HIV-negative women will be invited to participate and HIV-positive women will be counseled and referred to the Day Hospital as per standard of care. The HIV-negative women will then receive information about the risks and benefits of enrolling their infants in the study and, if interested, will be asked to sign a second informed consent (see below).

Pregnant women who attend the VCT center and finally enroll their infants in the study will only be asked to sign one informed consent. These women attend the VCT center voluntarily and not because the investigators have invited them to do so. At the VCT center, after they have been tested for HIV according to standard procedues, the counselor will only invite HIV-negative women to participate in the study and, if interested, will start the informed consent process to enroll their infants in the study (equivalent to the second informed consent of mother of infants aged 0-2 months).
(Amended 2 December, 2004)

Both oral and written modes will be used to provide information to pregnant women ***and mothers of infants aged 0-2 months old*** who are interested in the study. ~~At the initiation of the study, the informed consent process will be started,~~ ***Because only HIV-negative mothers can enroll their infants in the study, and investigators cannot have access to the HIV-status of the potential participants, the informed consent process will always be performed by a trained VCT center counselor and will take place at the VCT center of the Maragra or Manhiça antenatal clinic, to ensure confidentiality***. ~~A field worker~~ ***The VCT center counselor***, specifically trained, will review the informed consent form with the interested woman. Consent forms will be written in Changana and Portuguese, since the target population consists of people for whom Changana and/or Portuguese

are the primary languages. If a potential subject only speaks/reads a language other than Portuguese or Changana, he/she will be disqualified from participation because of inability to provide him/her with the adequate information necessary for informed consent. **(Amended 2 December, 2004)** **(Amended 10 August, 2005)**

Every attempt has been made to keep the consent form as brief as possible while providing full information about the risks and benefits of the study. The consent form is designed to provide general clinical trial education, information about one's rights as a study participant, study material and potential risks and benefits of the study in a form that is thorough while remaining easily understood. Ample time will be given for consideration of the consent forms and for any questions directed to the investigator before the consent is signed. ***Before signing the consent, the counselor will make sure that the woman has clearly understood the main issues of the study. All women will be asked to answer several questions on the most crucial aspects of the study, and if they do not answer correctly the counselor will clarify and explain these issues again. The informed consent will not be signed until the crucial questions have been answered correctly.*** For those women who are illiterate and unable to provide a signature, a thumbprint will be utilized as an alternative. In all cases, informed consent may be documented only by the dated signature (or thumbprint) of the volunteer and by ***the signature of the counselor*** ~~signatures of a witness and of the field worker~~. This signature (or thumbprint) provides documentation that the information about the study presented to the volunteer has been comprehended. Two identical copies of the consent form will be signed/thumbprinted. One of the copies will be provided to the volunteer and the other copy will be kept on file by the principal investigator.
(Amended 2 December, 2004)

b) Protection Against Risk
Blood draws from the infants will consist solely of fingerpricks (1-1.5 ml) and should not subject the child to any significant blood loss. Infants will be monitored by weekly active case detection from the start of the study. Any symptoms suggestive of potential side effects from the prophylaxis (for example skin rash) or signs or symptoms suggestive of malaria (including fever or history of fever) will be reported. In addition, parents will be carefully instructed about how to observe their infants for any signs or symptoms indicative of reaction to the prophylaxis or of infection and continuous access to health care will be available for all participating infants.

3. Potential Benefits of the Proposed Research to the Subjects and Others

a) Potential benefits to mother and infant
All infants will receive unlimited access to outpatient clinical care at the ~~Ilha Josina~~ ***Maragra*** Health Centre or at the Manhiça Hospital, as well as prompt malaria diagnosis and treatment. Infants will be monitored to minimize risk of complicated or severe disease caused by malaria, in a setting where the risk of this outcome is high. If the infant becomes severely ill, as a result of malaria or any other disease, accident, or injury, the CISM team will direct every resource at their disposal to evacuate that child from ~~Ilha Josina~~ ***Maragra*** to the Manhiça Hospital or to the Maputo Hospital if necessary. Mothers will be educated in malaria, malaria chemotherapy, and malaria prevention utilizing CISM technical staff and village-level gatherings. The benefits of receiving prophylaxis during the first year of life (Cohorts 1 and 2) are not clearly understood, but we do expect that infants will have a decreased risk of malaria during the specific time period when they are receiving prophylaxis. ***Furthermore the investigators will ensure that all participants receive the EPI vaccines at 2, 3, 4 and 9 months of age. Infants will be invited to the health post to receive these vaccines and***

road-to-health cards will be checked during the active case detection visits to make sure they have received them.
(Amended 15 October, 2004) (Amended 2 December, 2004)

b) Indirect Benefits
The study participants (mothers and infants) represent people who suffer the greatest in the face of endemic malaria. It is specifically this group of people who will benefit by the availability of a vaccine that prevents malaria. That is what drives this research protocol: developing an *in vitro* assay that greatly accelerates the development and deployment of a vaccine that prevents malaria.

4. Importance of the knowledge to be gained

We believe that the potential risks to the subjects in this proposal as described above are reasonable in relation to the importance of the knowledge that reasonably may be expected to result. The data resulting from this study should help us to understand the development of immune responses associated with the early stages of the NAI and the impact of age of first exposure on these responses. These studies should have an important influence on vaccination and other malaria-preventing strategies. **The most important implication of this work is with regard to administration of a malaria vaccine or of other strategies to prevent malaria and the design of trials.** For example, a better understanding of whether (and if so, why) young infants have difficulty in responding Pf antigens may help to clarify why in the SPf66 infant study [4] there was difficulty in inducing immune responses through vaccination in this population. Our results should lead to further studies evaluating the optimal timing during infancy for immunization against malaria as well as strategies to optimize infant immune responses with vaccination.

BIBLIOGRAPHY

1. Menendez C, Kahigwa E, Hirt R, Vounatsou P, Aponte JJ, Font F, Acosta CJ, Schellenberg DM, Galindo CM, Kimario J, Urassa H, Brabin B, Smith TA, Kitua AY, Tanner M, Alonso PL. Randomised placebo-controlled trial of iron supplementation and malaria chemoprophylaxis for prevention of severe anemia and malaria in Tanzanian infants. *Lancet* 1997; 350:844-50.

2. Schellenberg D, Menendez C, Kahigwa E, Aponte J, Vidal J, Tanner M, Mshinda H, Alonso PL. Intermittent treatment for malaria and anaemia control at time of routine vaccinations in Tanzanian infants: a randomised placebo-controlled trial. *Lancet* 2001; 357: 1471-7.

3. Alonso PL, Smith T, Schellenberg JRMA, Masanja H, Mwankusye S, Urassa H, Deazevedo IB, Chongela J, Kobero S, Menendez C, Hurt N, Thomas MC, Lyimo E, Weiss NA, Hayes R, Kitua AY, Lopez MC, Kilama WL, Teuscher T, Tanner M. Randomised trial of efficacy of SPf66 vaccine against *Plasmodium falciparum* malaria in children in southern Tanzania. *Lancet* 1994; 344, 1175-81.

4. Acosta CJ, Galindo CM, Schellenberg D, Aponte JJ, Kahigwa E, Urassa H, Schellenberg JR, Masanja H, Hayes R, Kitua AY, Lwilla F,Mshinda H, Menendez C, Tanner M, Alonso PL. Evaluation of the SPf66 vaccine for malaria control when delivered through the EPI scheme in Tanzania. *Trop Med Int Health* 1999; 4:368-76.

5. Hellgren U, Kihamia CM, Bergqvist Y, Lebbad M, Premji Z, Rombo. Standard and reduced doses of sulfadoxine-pyrimethamine for treatment of *Plasmodium falciparum* in Tanzania, with determination of drug concentrations and susceptibility *in vitro*. Trans R Soc Trop Med Hyg 1990; 84:469-72.

6. McGregor IA. Malarial immunity: current trends and prospects. Ann Trop Med Parasitol. 1987; 81(5):647-56.

7. McGregor IA, Gilles HM, Walters JH, Davies AH, Pearson FA. Effects of heavy and repeated malarial infections on Gambian infants and children; effects of erythrocyte parasitization. *British Med J* 1956; 2:686-92.

8. Baird JK. Host age as a determinant of naturally acquired immunity to *P. falciparum. Parasitology Today* 1995; 11:105-11.

9. Baird JK. Age-dependent characteristics of protection vs. susceptibility to *P. falciparum*. *Ann Trop Med Parasitol* 1998; 92:367-90.

10. Baird JK, Masbar S, Basri H, Tirtokusumo S, Subianto B, Hoffman SL. Age-dependent susceptibility to severe disease with primary exposure to *P. falciparum*. *J Infect Dis* 1998; 178:592-5.

11. Baird JK, Jones TR, Danudirgo EW, Annis BA, Bangs MJ, Basri H, Purnomo, Masbar S. Age-dependent acquired protection against *P. falciparum* in people having two years exposure to hyperendemic malaria. *Am J Trop Med Hyg* 1991; 45:65-76.

12. Baird JK, Purnomo, Basri H, Bangs MJ, Andersen EM, Jones TR, Masbar S, Harjosuwarno S, Subianto B, Arbani PR. Age specific prevalence of *P. falciparum* among six populations with limited histories of exposure to endemic malaria. *Am J Trop Med Hyg* 1993;49:707-19.

13. Oeuvray C, Bouharoun-Tayoun H, Grasmasse H, Bottius E, Kaidoh T, Aikawa M, Filgueira MC, Tartar A, Druilhe P. Merozoite surface protein-3: a malaria protein inducing antibodies that promote *Plasmodium falciparum* killing by cooperation with blood monocytes. *Blood* 1994; 84:1594-1602.

14. Cohen S, McGregor IA, Carrington S. Gamma globulin and acquired immunity to human malaria. *Nature* 1961; 192:733-7.

15. McGregor IA, Carrington S, Cohen S. Treatment of East African *P. falciparum* malaria with West African human gamma-globulin. *Trans Roy Soc Trop Med Hyg* 1963; 57:170-5.

16. Bouharoun-Tayoun H, Attanath P, Sabchareon A, Chongsuphajaisiddhi T, Druilhe P. Antibodies that protect humans against *P. falciparum* blood stages do not on their own inhibit parasite growth and invasion *in vitro*, but act in cooperation with monocytes. *J Exp Med* 1990; 172:1633-41.

17. Sabchareon A, Burnouf T, Ouattara D, Attanath P, Bouharoun Tayoun H, Chantavanich P, Foucault C, Chongsuphajaisiddhi T, Druilhe P. Parasitologic and clinical human response to immunoglobulin administration in falciparum malaria. *Am J Trop Med Hyg* 1991: 45:297-308.

18. Al-Yaman F, Genton B, Kramer KJ, Chang SP, Hui GS, Baisor M, Alpers MP. Assessment of the role of naturally acquired antibody levels to *Plasmodium falciparum* merozoite surface protein-1 in protecting Papua New Guinean children from malaria morbidity. *Am J Trop Med Hyg* 1996; 54:443-8.
19. Riley EM, Allen SJ, Wheeler JG, Blackman MJ, Bennett S, Takacs B, Schonfeld HJ, Holder AA, Greenwood BM. Naturally acquired cellular and humoral immune responses to the major merozoite surface protein (MSP1) of *P. falciparum* are associated with reduced malaria morbidity. *Parasite Immunol* 1992; 14:321-37.

20. Riley EM, Morris-Jones S, Blackman MJ, Greenwood BM, Holder AA. A longitudinal study of naturally acquired cellular and humoral immune responses to a merozoite surface protein (MSP1) of *P. falciparum* in an area of seasonal malaria transmission. *Parasite Immunol* 1993; 15:513-24.

21. Hill AVS, Elvin J, Willis AC, Aidoo M, Allsopp CEM, Gotch FM, Gao XM, Takiguchi M, Greenwood BM, Townsend ARM, McMichael AJ, Whittle HC. Molecular analysis of the association of HLA-B53 and resistance to severe malaria. *Nature* 1992; 360:434-9.

22. Hoffman SL, Oster CN, Mason C, Beier JC, Sherwood JA, Ballou WR, Mugambi M, Chulay JD. Human lymphocyte proliferative response to a sporozoite T cell epitope correlates with resistance to falciparum malaria. *J Immunol* 1989; 142: 1299-303.

23. Molineaux L, Cornille-Brogger R, Mathews HM, Storey J. Longitudinal serological study of malaria in infants in the West African savanna. *Bull World Health Org* 1978; 56:573-8.

24. Cornille-Brogger R, Mathews HM, Storey J, Ashkar TS, Brogger S, Molineaux L. Changing patterns in the humoral immune response to malaria before, during, and after the application of control measures: a longitudinal study in the West African savanna. *Bull World Health Org* 1978; 56:579-600.

25. Bradley-Moore AM, Greenwood BM, Bradley AK, Bartlett A, Bidwell DE, Voller A, Kirkwood BR, Gilles HM. Malaria chemoprophylaxis with chloroquine in young Nigerian children: Parts I-IV. *Annals of Tropical Med Parasitol* 1985; 79: 549-95.

26. Greenwood BM, Bradley AK, Greenwood AM, Byass P, Jammeh K, Marsh K, Tulloch S, Oldfield FSJ, Hayes R. Mortality and morbidity from malaria among children in a rural area of The Gambia, West Africa. Trans R Soc Trop Med Hyg 1987; 81:478-486.

27. Marsh K, Otoo L, Hayes RJ, Carson DC, Greenwood BM. Antibodies to blood stage antigens of *Plasmodium falciparum* in rural Gambians and their relation to protection against infection. *Trans R Soc Trop Med Hyg* 1989; 83:293-303.

28. Marsh K, Howard RJ. Antigens induced on erythrocytes by *P. falciparum*: expression of diverse and conserved determinants. S*cience* 1986; 231:150-3.

29. Bull PC, Loew BS, Kortok M, Molyneux CS, Newbold CI, Marsh K. Parasite antigens on the infected red cell surface are targets for naturally acquired immunity to malaria*. Nat Med* 1998; 4:358-60.

30. Giha HA, Staalsoe T, Dodoo D, Roper C, Satti GM, Arnot DE, Hviid L, Theander TG. Antibodies to variable Plasmodium falciparum-infected erythrocyte surface antigens are associated with protection from novel malaria infections.
Immunol Lett. 2000 Feb 1;71(2):117-26.

31. Dodoo D, Staalsoe T, Giha H, Kurtzhals JA, Akanmori BD, Koram K, Dunyo S, Nkrumah FK, Hviid L, Theander TG. Antibodies to variant antigens on the surface of infecyted erythrocytes are associated with protection from malaria in Ghanaian children. *Infect Immun* 2001; 69:3713-8.

32. Forsyth KP, Philip G, Smith T, Kum E, Southwell B, Brown GV. Diversity of antigens expressed on the surface of erythrocytes infected with mature *P. falciparum* parasites in Papua New Guinea. *Am J Trop Med Hyg* 1989;41:259-65.

33. Rogerson SJ, Beck HP, Al-Yaman F, Currie B, Alpers MP, Brown GV. Disruption of erythrocyte rosettes and agglutination of erythrocytes infected with Plasmodium falciparum by the sera of Papua New Guineans. *Trans R Soc Trop Med Hyg.* 1996 Jan-Feb;90(1):80-4.

34. Riggione F, Pulido M, Noya O. Plasmodium falciparum: surface modifications of infected erythrocytes from clinical isolates. Evidence of antigenic diversity using Venezuelan human malarial sera. *Parasitol Res.* 1996;82(6):490-6.

35. Newbold CI, Pinches R, Roberts DJ, Marsh K. *Plasmodium falciparum*: the human agglutinating antibody-response to the infected red-cell surface is predominantly variant specific. *Exp Parasitol* 1992; 75:281-92.

36. John CC, Sumba PO, Ouma JH, Nahlen BL, King CL, Kazura JW. Cytokine responses to *Plasmodium falciparum* liver-stage antigen 1 vary in rainy and dry seasons in highland Kenya. *Infect Immun* 2000; 68:5198-204.

37. Luty AJ, Lell B, Schmidt-Ott R, Lehman LG, Luckner D, Greve B, Matousek P, Herbich K, Schmid D, Migot-Nabias F, Deloron P, Nussenzweig RS, Kremsner PG. Interferon-gamma responses are associated with resistance to reinfection with *P. falciparum* in young African children. *J Infect Dis* 1999; 179:980-8.

38. Grau GE, Taylor TE, Molyneux ME, Wirima JJ, Vassalli P, Hommel M, Lambert PH. Tumor necrosis factor and disease severity in children with falciparum malaria. *New Eng J Med* 1989; 320:1586-91.

39. Bouharoun-Tayoun H, Oeuvray C, Lunel F, Druilhe P. Mechanisms underlying the monocyte-mediated antibody-dependent killing of *P. falciparum* asexual blood stages. *J Exp Med* 1995; 182:409-18.

40. Troye-Blomberg M, Riley EM, Kabilan L, Holmberg M, Perlmann H, Andersson U, Heusser CH, Perlmann P. Production by activated human T-cells of interleukin-4 but not interferon-gamma is associated with elevated levels of serum antibodies to activating malaria antigens. *Proc Nat Acad Sci USA* 1990; 87:5484-8.

41. Winkler S, Willheim M, Baier K, Schmid D Aichelburg A, Graninger W, Kremsner PG. Reciprocal regulation of Th1- and Th2- cytokine-producing T cells during clearance of parasitemia in *P. falciparum* malaria. *Inf Imm* 1998; 66:6040-4.

42. Kurtzhals JAL, Adabayeri V, Goka BQ, Akanmori BD, Oliver-Commey JO, Nkhrumah FK, Behr C, Hviid L. Low plasma concentrations of interleukin 10 in severe malarial anaemia compared with cerebral and uncomplicated malaria. *Lancet* 1998; 351: 1768-72

43. Plebanski M, Flanagan KL, Lee EA, Reece WH, Hart K, Gelder C, Gillespie G, Pinder M, Hill AV. Interleukin 10-mediated immunosuppression by a variant CD4 T cell epitope of *Plasmodium falciparum. Immunity* 1999; 10:651-60.

44. Luty AJ, Perkins DJ, Lell B, Schmidt-Ott R, Lehman LG, Luckner D, Greve B, Matousek P, Herbich K, Schmid D, Weinberg JB, Kremsner PG. Low Interleukin-12 activity in severe *P. falciprum* malaria. *Inf Imm* 2000; 68:3909-15.

45. Perkins DJ, Weinberg JB, Kremsner PG. Reduced Interleukin-12 and transforming growth factor– beta in severe childhood malaria: relationship of cytokine balance with disease severity. *J Infect Dis* 2001; 182:988-92.

46. Binka FN, Kubaje A, Adjuik M, Williams LA, Lengeler C, Maude GH, Armah GE, Kajihara B, Adiamah JH, Smith PG. Impact of permethrin impregnated bednets on child mortality in Kassena-Nankana District, Ghana: a randomized controlled trial. *Trop Med Int Health* 1996; 1:147-54.

47. Siegrist C-A. Neonatal and early life vaccinology. *Vaccine* 2001; 19:3331-46.

48. D'Alessandro U, Leach A, Drakeley CJ, Bennett S, Olaleye BO, Fegan GW, Jawara M, Langerock P, George MO, Targett GAT, Greenwood BM. Efficacy trial of malaria vaccine SPf66 in Gambian infants. *Lancet* 1995; 346:462-7.

49. Galindo CM, Acosta CJ, Schellenberg D, Aponte JJ, Roca A, Oettli A, Urassa H, Armstrong Schellenberg J, Kahigwa E, Ascaso C, Mshinda H, Lwilla F, Vidal J, Menendez C, Tanner M, Alonso PL. Humoral immune responses during a malaria vaccine trial in Tanzanian infants. *Parasite Immunol* 2000; 22:437-43

50. Schellenberg D, Menendez C, Kahigwa E, Font F, Galindo C, Acosta C, Schellenberg JA, Aponte JJ, Kimario J, Urassa H, Mshinda H, Tanner M, Alonso P. African children with malaria in an area of intense *Plasmodium falciparum* transmission: features on admission to the hospital and risk factors. for death *Am J Trop Med Hyg* 1999; 61:431-438.

51. Alonso PL, Saute F, Aponte JJ, Gomez-Olive FX, Nhacolo A, Thomson R, Macete E, Abacassomo F, Ventura PJ, Bosch S, Menendez C, Dgedge M. *Indepth Monograph: Manhiça DSS, Mozambique* 2001. Vol 1 Part C, Chapter 15, 295-308.

52. Menendez C, Ordi J, Ismail MR, Ventura PJ, Aponte JJ, Kahigwa E, Font F, Alonso PL. The impact of placental malaria on gestational age and birth weight. *J Infect Dis* 2000; 181:1740-5.

53. Ismail M, Ordi J, Menendez C, Ventura PJ, Aponte JJ, Kahigwa E, Hirt R, Cardesa A, Alonso PL. Placental pathology in malaria: A histological, immunohisto-chemical, and quantitative study. *Hum Pathol* 2000; 31:85-93.

54. Mayor AG, Gomez-Olive X, Aponte JJ, Casimiro S, Mabunda S, Dgedge M, Barreto A, Alonso PL. Prevalence of the K76T mutation in the putative Plasmodium falciparum chloroquine resistance transporter (pfcrt) gene and its relation to chloroquine resistance in Mozambique. *J Infect Dis*. 2001 May 1;183(9):1413-6. Epub 2001 Mar 30.

55. Saute F, Menendez C, Mayor A, Aponte J, Gomez-Olive X, Dgedge M, Alonso PL. Malaria in pregnancy in rural Mozambique: the role of parity, submicroscopic and multiple *Plasmodium falciparum* infections. *Trop Med Int Health* 2002; 7(1):19-28.

56. Malik A, Egan JE, Houghton RA, Sadoff JC, Hoffman SL. Human cytotoxic T lymphocytes against *Plasmodium falciparum* circumsporozoite protein *Proc Natl Acad Sci USA* 1991; 88:3300-4.

57. Doolan DL, Hoffman SL, Southwood S, Wentworth PA, Sidney J., Chesnut RW, Keogh E, Appella E, Nutman TB, Lal AA, Gordon DM, Oloo A, and Sette A. Degenerate cytotoxic T cell epitopes from *P. falciparum* restricted by multiple HLA-A and HLA-B supertype alleles. *Immunity* 1997; 7:97-112.

58. Doolan DL, Southwood S, Chesnut R, Appella E, Higashimoto YI, Maewal A, Gomez E, Sidney J, Richards A, Gramzinski RA, Hoffman SL, Sette A. HLA-DR-promiscuous T cell epitopes from *Plasmodium falciparum* restricted by multiple HLA Class II alleles. *J Immunol* 2000; 165:1123-37.

59. Doolan DL, Houghten RA, Good MF. Assessment of human cytotoxic T cell activity using synthetic peptides: potential for field application. *Peptide Research* 1991; 4:125-31.

59. Hoffman SL, Isenbarger D, Long GW, Sedegah M, Szarfman A, Waters L, Hollingdale MR, van der Miede PH, Finbloom DS, Ballou WR. Sporozoite vaccine induces genetically restricted T cell elimination of malaria from hepatocytes *Science* 1989; 244:1078-81

60. Doolan DL, Houghten RA, Good MF. Location of human cytotoxic T cell epitopes within a polymorphic domain of the *Plasmodium falciparum* circumsporozoite protein. *Int Immunol* 1991; 3:511-6.

61. Sedegah M, Sim BKL, Mason C, Nutman T, Malik A, Roberts C, Johnson A, Ochola J, Koech D, Were B, Hoffman SL. Naturally acquired CD8+ cytotoxic T lymphocytes against the *Plasmodium falciparum* circumsporozoite protein. *J Immunol* 1992; 149:966-971

62. Doolan DL, Khamboonruang C, Beck H-P, Good MF. Cytotoxic T lymphocyte low-responsiveness to the *Plasmodium falciparum* circumsporozoite protein in naturally-exposed endemic populations: analysis of human CTL response to all known variants. *Int Immunol* 1993; 5:37-46.

63. Wizel B, Houghten R, Church P, Tine JA, Lanar DE, Gordon DM, Ballou WR, Sette A, Hoffman SL. HLA-A2-restricted cytotoxic T lymphocyte responses to multiple *Plasmodium falciparum* sporozoite surface protein 2 epitopes in sporozoite-immunized volunteers. *J Immunol* 1998; 155:766-75.

64. Wizel B, Houghten RA, Parker K, Coligan JE, Church P, Gordon DM, Ballou WR, Hoffman SL. Irradiated sporozoite vaccine induces HLA-B8-restricted cytotoxic T lymphocyte responses against two overlapping epitopes of the *Plasmodium falciparum* surface sporozoite protein 2. *J Exp Med* 1995; 182: 1435-45.

65. Wang R, Doolan DL, Le TP, Hedstrom RC, Coonan KM, Charoenvit Y, Jones TR, Hobart P, Margalith M, Nj. J, Weiss WR, Sedegah M, de Taisne C, Norman J, Hoffman, SL.. Malaria DNA vaccine induces antigen specific genetically restricted cytotoxic T lymphocytes in humans. *Science* 1998; 282:476-80.

66. Wang R, Epstein J, Baraceros FM, Gorak EJ, Charoenvit Y, Carucci DJ, Hedstrom RC, Rahardjo N, Gay T, Hobart P, Stout R, Jones TR, Richie TL, Parker SE, Doolan DL, Norman J, Hoffman SL. Induction of CD4(+) T cell-dependent CD8(+) type 1 responses in humans by a malaria DNA vaccine. *Proc Natl Acad Sci U S A* 2001; 98:10817-22.

67. Sedegah M, Jones TR, Kaur M, Hedstrom RC, Hobart P, Tine JA, Hoffman SL. Boosting with recombinant vaccinia increases immunogenicity and protective efficacy of malaria DNA vaccine. *Proc Nat Acad Sci USA* 1998; 95:7648-53.

68. Kumar A, Weiss W, Tine JA, Hoffman SL, Rogers WO. ELISPOT assay for detection of peptide specific interferon-gamma secreting cells in rhesus macaques.
J Immunol Methods. 2001 Jan 1;247(1-2):49-60.

69. Weiss WR, Ishii KJ, Hedstrom RC, Sedegah M, Ichino M, Barnhart K, Klinman DM, Hoffman SL. A plasmid encoding murine granulocyte-macrophage colony-stimulating factor increases protection conferred by a malaria DNA vaccine. *J Immunol* 1998; 161:2325-32

70. Brice GT, Graber NL, Hoffman SL, Doolan DL. Expression of the chemokine MIG is a sensitive and predictive marker for antigen-specific, genetically restricted IFN-gamma production and IFN-gamma-secreting cells.
J Immunol Methods. 2001 Nov 1;257(1-2):55-69.

71. Haynes JD and Moch K. Automated synchronization of *P. falciparum* parasites by culture in a temperature-cycling incubator. *Meth Mol Med* 2002; In press.

72. Riley EM, Wagner GE, Ofori MF, Wheeler JG, Akanmori BD, Tetteh K, McGuinness D, Bennett S, Nkrumah FK, Anders RF, Koram KA. Lack of association between maternal antibody and protection of African infants from malaria infection. *Infect Immun* 2000; 68:5856-63.

73. Menendez C, Alonso PL, Kinteh A, M'Boge B, Francis N, Greenwood BM. The contribution of Gambian traditional birth attendants to field research. *J Trop Med Hyg* 1993; 96:175-8.

74. Bull PC, Loew BS, Kortok M, Marsh K. Antibody recognition of *Plasmodium falciparum* erythrocyte surface antigens in Kenya: evidence for rare and prevalent variants. *Infect Immun* 1999; 67: 733-9.

75. Bull PC, Kortok M, Kai O, Ndungu F, Ross A, Lowe BS, Newbold CI, Marsh K. *Plasmodium falciparum*-infected erythrocytes: Agglutination by diverse Kenyan plasma is associated with severe disease and young host age. *J Inf Dis* 2000; 182:252-9.

76. Cook EB, Stahl JL, Lowe L, Chen R, Morgan E, Wilson J, Varro R, Chan A, Graziano FM, Barney NP. Simultaneous measurement of six cytokines in a single sample of human tears using microparticle-based flow cytometry: allergics vs. non-allergics. *J Immunol Methods* 2001; 254:109-18.

77. Rowe JA, Scragg IG, Kwiatkowski D, Ferguson DJ, Carucci DJ, Newbold CI. Implications of mycoplasma contamination in *Plasmodium falciparum* cultures and methods for its detection and eradication. *Mol Biochem Parasitol* 1998; 92:177-80.

78. Snounou G, Zhu X, Siripoon N, Jarra W, Thaithong S, Brown KN, Viriyakosol S. Biased distribution of msp1 and msp2 allelic variants in Plasmodium falciparum populations in Thailand. Trans R Soc Trop Med Hyg. 1999 Jul-Aug;93(4):369-74.

80. Foley M, Ranford-Cartwright LC, Babiker HA. Rapid and simple method for isolating malaria DNA from fingerprick samples of blood. *Mol Biochem Parasitol* 1992; 53:241-4.

81. Ranford-Cartwright LC, Balfe P, Carter R, Walliker D. Frequency of cross-fertilization in the human malaria parasite *Plasmodium falciparum*. *Parasitology* 1993; 107:11-18.

82. Cavanagh DR, McBride JS. Antigenicity of recombinant proteins derived from *Plasmodium falciparum* merozoite surface protein 1. *Mol Biochem Parasitol* 1997; 85:197-211.

83. Reeder JC, Marshall VM. A simple method for typing *Plasmodium falciparum* merozoite surface antigens 1 and 2 (*MSA-1* and *MSA-2*) using a dimorphic-form specific polymerase chain reaction. *Mol Biochem Parasitol* 1994; 68:329-32.

84. Hill AVS, Allsopp CEM, Kwiatkowski D, Anstey NM, Twumasi P, Rowe PA, Bennett S, Brewster D, McMichael AJ, Greenwood BM. Common West African HLA antigens are associated with protection from severe malaria. *Nature* 1991; 352:595-600.

85. Williams TN, Maitland K, Bennett S, Ganczakowski M, Peto TE, Newbold CI, Bowden DK, Weatherall DJ, Clegg JB. High incidence of malaria in alpha-thalassaemic children. *Nature* 1996; 383:522-5.

86. Kitua AY, Urassa H, Wechsler M, Smith T, Vounatsou P, Weiss NA, Alonso PL, Tanner M. Antibodies against *Plasmodium falciparum* vaccine candidates in infants in an area of intense and perennial transmission: relationships with clinical malaria and with entomological inoculation rates. *Parasite immunol* 1999; 21:307-16.

87. Gordi T, Lepist EI. Artemisinin derivatives: toxic for laboratory animals, safe for humans? Toxicol Lett. 2004; 147(2):99-107

88. Adjuik M, Babiker A, Garner P, Olliaro P, Taylor W, White N; International Artemisinin Study Group. Artesunate combinations for treatment of malaria: meta-analysis. Lancet. 2004; 363(9402):9-17.

89. Abacassamo F, Enosse S, Aponte JJ, Gomez-Olive FX, Quinto L, Mabunda S, Barreto A, Magnussen P, Ronn AM, Thompson R, Alonso PL. Efficacy of chloroquine, amodiaquine, sulphadoxine-pyrimethamine and combination therapy with artesunate in Mozambican children with non-complicated malaria. Trop Med Int Health. 2004;9(2): 200-8.

90. Schofield, L., and Hackett, F. 1993. Signal transduction in host cells by a glycosylphosphatidylinositol toxin of malaria parasites. J. Exp. Med. 177:145-153
91. Schofield, L., Novakovic, S., Gerold, P., Schwarz, R.T., McConville, M.J., and Tachado, S.D. 1996. Glycosylphosphatidylinositol toxin of Plasmodium upregulates intercellular adhesion molecule-1, vascular cell adhesion molecule-1, and E-selectin expression in vascular endothelial cells and increases leukocyte and parasite cytoadherence via tyrosine kinase-dependent signal transduction. J. Immunol. 156:1886-1896.
92. Tachado, S.D., Gerold, P., Schwarz, R., Novakovic, S., McConville, M., and Schofield, L. 1997. Signal transduction in macrophages by glycosylphosphatidylinositols of Plasmodium, Trypanosoma and Leishmania: Activation of protein tyrosine kinases and protein kinase C by inositolglycan and diacylglycerol moieties. Proc. Natl. Acad. Sci. USA 94:4022-4027.
93. Almeida, I.C., Camargo, M.M., Procopio, D.O., Silva, L.S., Mehlert, A., Travassos, L.R., Gazzinelli, R.T., and Ferguson, M.A. 2000. Highly purified glycosylphosphatidylinositols from Trypanosoma cruzi are potent proinflammatory agents. EMBO J. 19:1476-1485.
94. Camargo, M.M., Almeida, I.C., Pereira, M.E., Ferguson, M.A., Travassos, L.R., and Gazzinelli, R.T. 1997. Glycosylphosphatidylinositol-anchored mucin-like glycoproteins isolated from Trypanosoma cruzi trypomastigotes initiate the synthesis of proinflammatory cytokines by macrophages. J. Immunol. 158:5890-5901.
95. Camargo, M.M., Andrade, A.C., Almeida, I.C., Travassos, L.R., and Gazzinelli, R.T. 1997. Glycoconjugates isolated from Trypanosoma cruzi but not from Leishmania species membranes trigger nitric oxide synthesis as well as microbicidal activity in IFN-g-primed macrophages. J. Immunol. 159:6131-6139.
96. Magez, S., Stijlemans, B., Radwanska, M., Pays, E., Ferguson, M.A., and DeBaetselier, P. 1998. The glycosyl-inositol-phosphate and dimyristoylglycerol moieties of the glycosylphosphatidylinositol anchor of the trypanosome variant-specific surface glycoprotein are distinct macrophage-activating factors. J. Immunol. 160:1949-1956.
97. Schofield, L., Hewitt, M.C., Evans, K., Siomos, M.A., and Seeberger, P.H. 2002. Synthetic GPI as a candidate anti-toxic vaccine in a model of malaria. Nature 418:785-789.
98. Fernandez-Reyes, D., et al., A high frequency African coding polymorphism in the N-terminal domain of ICAM-1 predisposing to cerebral malaria in Kenya. Hum Mol Genet, 1997. 6(8): p. 1357-60.
99. Aitman, T.J., et al., Malaria susceptibility and CD36 mutation. Nature, 2000. 405(6790): p. 1015-6.
100. Pain, A., et al., A non-sense mutation in Cd36 gene is associated with protection from severe malaria. Lancet, 2001. 357(9267): p. 1502-3.
101. Burgner, D., et al., Nucleotide and haplotypic diversity of the NOS2A promoter region and its relationship to cerebral malaria. Hum Genet, 2003. 112(4): p. 379-86.
102. Xiang, L., et al., Quantitative alleles of CR1: coding sequence analysis and comparison of haplotypes in two ethnic groups. J Immunol, 1999. 163(9): p. 4939-45.
103. Luty, A.J., J.F. Kun, and P.G. Kremsner, Mannose-binding lectin plasma levels and gene polymorphisms in Plasmodium falciparum malaria. J Infect Dis, 1998. 178(4): p. 1221-4.
104. McGuire, W., et al., Variation in the TNF-alpha promoter region associated with susceptibility to cerebral malaria. Nature, 1994. 371(6497): p. 508-10.
105. Prescott, S.L., et al., Development of allergen-specific T-cell memory in atopic and normal children

Raised serum IgE associated with reduced responsiveness to DPT vaccination during infancy Transplacental priming of the human immune system to environmental allergens: universal skewing of initial T cell responses toward the Th2 cytokine profile TH2-polarized immunological memory to inhalant allergens in atopics is established during infancy and early childhood. Lancet, 1999. 353(9148): p. 196-200.

106. Rowe, J., et al., Antigen-specific responses to diphtheria-tetanus-acellular pertussis vaccine in human infants are initially Th2 polarized. Infect Immun, 2000. 68(7): p. 3873-7.

107. Verra, F., et al., IL4-589C/T polymorphism and IgE levels in severe malaria. Acta Trop, 2004. 90(2): p. 205-9.

108. Ohashi, J., et al., A single-nucleotide substitution from C to T at position -1055 in the IL-13 promoter is associated with protection from severe malaria in Thailand. Genes Immun, 2003. 4(7): p. 528-31.

109. Morahan, G., et al., Association of IL12B promoter polymorphism with severity of atopic and non-atopic asthma in children. Lancet, 2002. 360(9331): p. 455-9.

110. Ohashi, J., et al., Lack of association between interleukin-10 gene promoter polymorphism, -1082G/A, and severe malaria in Thailand. Southeast Asian J Trop Med Public Health, 2002. 33 Suppl 3: p. 5-7.

111. Henri, S., et al., Description of three new polymorphisms in the intronic and 3'UTR regions of the human interferon gamma gene. Genes Immun, 2002. 3(1): p. 1-4.

112. Pask, R., et al., Investigating the utility of combining phi29 whole genome amplification and highly multiplexed single nucleotide polymorphism BeadArray genotyping. BMC Biotechnol, 2004. 4(1): p. 15.

(Amended 11 July, 2005)
